# Adapting the facial action coding system for chimpanzees (*Pan troglodytes*) to bonobos (*Pan paniscus*): the ChimpFACS extension for bonobos

**DOI:** 10.7717/peerj.19484

**Published:** 2025-06-13

**Authors:** Catia Correia-Caeiro, Paul Henrik Kuchenbuch, Linda S. Oña, Franziska Wegdell, Maël Leroux, André Schuele, Jared Taglialatela, Simon Townsend, Martin Surbeck, Bridget M. Waller, Katja Liebal

**Affiliations:** 1 Human Biology & Primate Cognition, Institute of Biology, Universität Leipzig, Leipzig, Germany; 2 Department of Comparative Cultural Psychology, Max Planck Institute for Evolutionary Anthropology, Leipzig, Germany; 3 Independent Researcher, Berlin, Germany; 4 Department of Evolutionary Anthropology, University of Zurich, Zurich, Switzerland; 5 Institute for the Interdisciplinary Study of Language Evolution, University of Zurich, Zurich, Switzerland; 6 Éthologie Animale et Humaine (EthoS), CNRS, University of Rennes, Normandie University, Rennes, France; 7 Berlin Zoo, Berlin, Germany; 8 Ape Cognition and Conservation Initiative, Des Moines, IA, United States of America; 9 Department of Ecology, Evolution, and Organismal Biology, Kennesaw State University, Kennesaw, GA, United States of America; 10 Department of Human Evolutionary Biology, Harvard University, Cambridge, MA, United States of America; 11 Department of Human Behavior, Ecology and Culture, Max Planck Institute for Evolutionary Anthropology, Leipzig, Germany; 12 Department of Psychology, Nottingham Trent University, Nottingham, United Kingdom

**Keywords:** FACS, Bonobos, Facial expressions, Facial movements, Homology, Face, Anatomy, Communication, Observational tool, Methodology

## Abstract

The Facial Action Coding System (FACS) is a widely recognised coding scheme for analysing human facial behaviour, providing an objective method to quantify discrete movements associated with facial muscles, known as Action Units (AUs), and reducing subjective bias. FACS has been adapted for nine other taxa, including apes, macaques, and domestic animals, but not yet bonobos. To carry out cross species studies of facial behaviours within and beyond apes, it is essential to include bonobos. Hence, we aimed at adapting FACS for bonobos. We followed a similar methodology as in previous FACS adaptations: first, we examined the facial muscular plan of bonobos from previously published dissections. Given the similarity between bonobo and chimpanzee musculature, we tested if ChimpFACS for chimpanzees could be applied to bonobos. Second, we used ChimpFACS to analyse spontaneous facial behaviour in bonobos through videos recorded in various contexts. Third, we noted any differences in appearance changes between the AUs included in ChimpFACS and the AUs observed in bonobos. Our findings showed that bonobos exhibit all the facial movements observed in chimpanzees, and thus ChimpFACS can reliably be applied to bonobos. Bonobos presented a diverse repertoire of 28 facial movements (22 AUs, three Action Descriptors, and three Ear Action Descriptors). Although the range of facial movement is lower than in humans, bonobo’s potential for facial movement is comparable to that of chimpanzees, underscoring the significance of this behaviour modality during social interactions for both species. The ChimpFACS Extension for bonobos is an objective coding scheme for measuring facial movements in bonobos, designed to be used in conjunction with ChimpFACS. This coding scheme extension will allow us to better understand bonobos’ behaviour and communication, with practical applications for assessing their welfare, particularly in human care. It also provides a framework for comparing primate species, contributing to insights into the origin and evolution of facial emotion and communication.

## Introduction

### The Facial Action Coding Systems for humans and other animals

The Facial Action Coding System (FACS) has been widely used in human facial behaviour research since Ekman and colleagues introduced it as a training manual (Ekman & Friesen, 1978; Ekman, Friesen & Hager, 2002a; Ekman, Friesen & Hager, 2002b). FACS is a standardised observational coding scheme designed to classify and describe human facial movements by linking them to their corresponding underlying musculature. When a facial muscle contracts, portions of the skin move, resulting in visible appearance changes on the face. These movements, driven by mimetic muscles, are categorised as Action Units (AUs), each identified by a numerical code and a descriptive label. For example, “AU16” represents the “Lower Lip Depressor”, which is coded when the depressor labii inferioris muscle pulls the lower lip downwards. To account for broader movements involving non-mimetic muscles, the human FACS manual also describes Action Descriptors (ADs), which capture movements like tongue actions or head and eye positions that can influence the visual changes associated with AUs. Therefore, FACS categorises discrete subunits of movement (*i.e.,* AUs) rather than global facial behaviours with multiple AUs (Calder et al., 2000). This approach minimises subjective interpretations of facial behaviours, such as emotion labels. FACS also accommodates individual variability in facial morphology (*e.g.*, differences in face shape, size, or permanent wrinkles) by utilizing consistent facial landmarks across individuals and establishing minimum criteria for identifying each AU. Determining a neutral face for each individual is particularly valuable for coding video data and essential for analysing still images, as it helps distinguish individual-specific features and prevents false indicators during the coding process (Vick et al., 2007).

Following the same methodology as used for the human system (Ekman, Friesen & Hager, 2002a), FACS has been adapted to be applied in several other primate species: chimpanzees (ChimpFACS, Vick et al., 2007), rhesus (Parr et al., 2010), Barbary (Julle-Danière et al., 2015), Japanese (Correia-Caeiro, Holmes & Miyabe-Nishiwaki, 2021), and crested (Clark et al., 2020) macaques (MaqFACS), hylobatids (GibbonFACS, Waller et al., 2012), orangutans (OrangFACS, Correia-Caeiro et al., 2013), gorillas (GorillaFACS, Correia-Caeiro et al., 2025), and common marmosets (CalliFACS, Correia-Caeiro et al., 2022). It has also been adapted to three domesticated species: dogs (DogFACS, Waller et al., 2013), horses (EquiFACS, Wathan et al., 2015), and cats (CatFACS, Correia-Caeiro, Burrows & Waller, 2017). The adaptation of FACS for other species is based on the examination of muscular homologies (*e.g.*, Burrows et al., 2006; Burrows et al., 2011; Burrows, Waller & Parr, 2009) while considering differences in facial morphology between species. Using AnimalFACS to measure facial movements has the following advantages: (1) the coding is independent of emotion labels and it is more observer-independent; (2) it provides a clear and common terminology that is used across species; (3) as the system is anatomically based, homologies can be recognised at the anatomical and behavioural level at once; (4) the bottom-up approach allows for the recognition of more details in facial behaviour.

The development of all these AnimalFACS has not only allowed new insights into the objective and standardised study of animal communication within each species, but also created a robust framework for inter-specific comparative and evolutionary perspectives on facial communication and emotional processes (Waller, Julle-Daniere & Micheletta, 2020; Kret et al., 2020; Kavanagh et al., 2022). For example, by applying AnimalFACS, novel insights have been found into how dogs and cats communicate with humans (Waller et al., 2013; Correia-Caeiro, Burrows & Waller, 2017; Humphrey et al., 2020) and react facially in emotional contexts (Correia-Caeiro, Guo & Mills, 2017; Bennett, Gourkow & Mills, 2017; Bremhorst et al., 2019; Llewelyn & Kiddie, 2022). In non-human primates (NHP), a growing number of studies applying AnimalFACS have been instrumental in understanding the complexities of communication and emotion in a variety of species. Some examples of these findings revealed that orangutans (Waller, Correia-Caeiro & Davila-Ross, 2015) and gibbons (Scheider et al., 2016) use their play faces flexibly, and in line with some intentionally criteria used for gestures (Leavens, Hopkins & Thomas, 2004; Liebal et al., 2014); that chimpanzees produce the same play face configuration independently of the vocalisations (Davila-Ross et al., 2015); that the same facial behaviour in crested macaques (Silent-Bared Teeth) has different meanings depending on which AUs are included in the display (Clark et al., 2020); that hylobatids pair-bonding is related to facial behaviour (Florkiewicz, Skollar & Reichard, 2018); and that species previously thought to have less facial mobility, such as common marmosets, have a similar potential for facial movements as other NHP (Correia-Caeiro et al., 2022). One of the first studies directly comparing the facial morphology and complexity of facial repertoires in chimpanzees and gibbons found that chimpanzees have a larger and more complex facial signal repertoire compared to gibbons (Florkiewicz et al., 2024). This finding supports the socio-ecological complexity hypothesis, which entails that social needs shape the evolution of facial repertoires (Florkiewicz et al., 2024). In addition to socio-ecological factors, there is some evidence that individual factors such as species, age, or sex of the sender and receiver may affect the AUs displayed (Crepaldi et al., 2024). These studies demonstrate the highly complex and dynamic nature of facial behaviour and highlight the role of FACS for the functional understanding of facial behaviour. This facial behaviour complexity often takes place within dynamic social interactions (*e.g.*, with multiple senders and receivers that display multiple AUs over time), which adds even more complexity to these behaviours and its interpretation. To try to tackle some of this complexity, the production of AUs has recently been examined with NetFACS, an R package which uses network analysis applied to rich FACS databases to understand communicative functions (Mielke et al., 2022; Rincon et al., 2023).

### Facial behaviour of bonobos

Bonobos are generally agreed to be a species with high facial mobility (De Waal, 1988; Bard, Gaspar & Vick, 2011), producing facial behaviours in varied contexts such as play (Palagi, 2006; Palagi, 2008; Palagi & Paoli, 2007; Bertini et al., 2022), sex (Palagi et al., 2020), social tension (Vlaeyen et al., 2022), and decision-making (Rosati & Hare, 2013), among others (De Waal, 1988; Gaspar, Esteves & Arriaga, 2014). However, these studies mostly investigated facial behaviours functionality, whilst little was said about the morphological aspects of each facial behaviour, *i.e.,* what each facial behaviour actually looks like or if there are variations in form within context. This is important to consider since there is considerable variation in how researchers have described facial behaviours in this species, with facial ethograms including between 5 to 46 holistic facial behaviours (De Waal, 1988; Pollick & De Waal, 2007; Bard, Gaspar & Vick, 2011). This large range previously reported may be related to a priori selectivity bias of facial behaviours where researchers focus only on some relevant facial behaviours for the discussion, or may be due to variation or disagreement of what each facial behaviour looks like (or what constitutes a display).

Studies on bonobo behaviour have attributed a variety of labels to observed facial behaviours in this species; for example, “relaxed face”, “stare face”, “pouting of the lips”, “grin face” (Patterson, 1979), “silent teeth-baring”, “tense mouth”, “silent pout”, “duck face”, “play face”, and “funny faces” (De Waal, 1988). Other than these generic terms, Patterson (Patterson, 1979) did not clearly define each facial behaviour or describe their appearance in detail, but only briefly compared them with displays described in chimpanzees. De Waal’s study (De Waal, 1988) included a more detailed description of each facial behaviour component along with contextual information and potential social function. However, in both studies, the application of categorical labels that are generic, emotional, or contextually-bound does not aid in identification of the actual facial behaviour, making it difficult to translate across studies, and may even bias the research questions. Furthermore, it excludes the possibility of small morphological variations in the same category of facial behaviour, which leads to functional and contextual use variation, in both humans (Ekman & Friesen, 1982) and NHP (Clark et al., 2020).

Comparisons of facial behaviour between humans, chimpanzees, and bonobos have been made by either directly applying the human FACS to bonobo and chimpanzee facial behaviour (Gaspar, 2001; Dobson, 2009a; Dobson, 2009b) or by creating a similar sub-unitary coding system based only on appearance changes but not AUs nor its underlying muscle basis, and lacking inter-rater reliability (Gaspar, 2001; Bard, Gaspar & Vick, 2011). For example, during sexual excitement or “happiness” contexts, all three species were noted to display similar core AUs (*e.g.*, AU6—Cheek Raiser + 12—Lip Corner Puller + 25—Lips Part + 26—Jaw Drop in joy/play, AU43—Eye Closure + AU26—Jaw Drop in sexual excitement) (Gaspar, Esteves & Arriaga, 2014). Despite a couple of idiosyncratic AUs found in each species, such as AU15—Lip Corner Depressor only found in chimpanzees or AU20—Lip Stretcher only found in humans (Dobson, 2009a; Dobson, 2009b), species-specific patterns were mostly found in combination and frequency of AUs (Bard, Gaspar & Vick, 2011). However, without species-specific FACS (*i.e.,* including examination of anatomical, morphological, and behavioural differences for each species independently, and inter-rater reliability), it is hard to understand the validity of these assessments (Bard, Gaspar & Vick, 2011). An over-estimation of how many AUs a species is able to display seems to happen if the human FACS is directly used for other species, since studies have reported AUs in apes that the species-specific FACS did not find (*e.g.*, chimpanzee studies with human FACS present 18 core AUs *vs.* 12 core AUs with ChimpFACS, Gaspar, 2006; Vick et al., 2007; Dobson, 2009a; Dobson, 2009b; Bard, Gaspar & Vick, 2011). This over-estimation might be due to a variety of reasons, such as not accounting for the muscular basis of the movements (Bard, Gaspar & Vick, 2011), including non-communicative contexts such as mastication (Dobson, 2009a; Dobson, 2009a), overlooking morphological differences between species that affect minimum criteria to code each AU, coding false indicators, disregarding independence and reliability of each AU, or relying only on longitudinal intra-rater reliability. For example, AU15—Lip Corner Depressor has been reported in chimpanzees and AU23—Lip Tightener in several primate species (Dobson, 2009a; Dobson, 2009b), but both movements have never been reliably coded as an independent AU and are therefore not included in the corresponding species-specific FACS (Vick et al., 2007; Parr et al., 2010; Waller et al., 2012; Correia-Caeiro et al., 2025).

Another study applied FACS and ChimpFACS in a cross-species study including bonobos to examine play faces in mother-infant interactions, revealing both a prototypical play face in all species examined at 6 months old and diverging types within this facial behaviour in terms of AUs (Lembeck, 2015; Liebal, Schneider & Errson-Lembeck, 2019). Whilst this study was the first cross-species application of FACS to infants and contributed new developmental insights of facial behaviour, it is not known to what extent FACS can be applied to infant NHP, as there might be some anatomical or morphological differences. A BabyFACS adaptation from the human (adult) FACS to infants and young children found some differences in displayed AUs, such as the AU3—Brow Knitting and Knotting which in the adult FACS is coded as AU4—Brow Lowerer, as appearance changes vary between infants and adults likely due to large facial morphological differences (Oster, 2005).

For a more detailed description of the issues with the lack of a species-specific FACS for bonobos see Kuchenbuch (2010).

### Why do bonobos need a FACS?

Despite facial anatomical and morphological similarities being assumed between chimpanzees and bonobos, and subsequent direct applications of FACS/ChimpFACS having been done for bonobos (Dobson, 2009a; Dobson, 2009b; Bard, Gaspar & Vick, 2011), it is important to consider potential differences between the two species, including at an anatomical, morphological, and behavioural level. The potential differences should then be highlighted in the appearance changes lists, minimum criteria, and photo/video examples of the new species. And more importantly, as all FACS are based on training and certification of users that reach a minimum reliability level (70%) with the developers of each system, it is crucial to test the inter-rater reliability of FACS application for each species separately, in this case bonobos. Given this information and the potential issues of over- or under-estimation of AUs arising from direct application of FACS/ChimpFACS directly to other species (see previous section), it is crucial to ensure that the original FACS methodology is followed (to maintain comparative power of the systems) and that a new system or extension is developed for each new species (to maintain inter-rater reliability of the systems).

Additional reasons underscore the need for a FACS for bonobos. As evidenced by the studies in the previous section, bonobos are a highly social species that seem to present high facial mobility, although they are considerably less studied than chimpanzees in general, but also in relation to their facial communication (Furuichi, 2019, see also Table SI1 in Text SI1 for a rough estimation of number of studies in the seven great ape species).

Bonobos are different from chimpanzees behaviourally and are regarded as showing some unusual social behaviour within the primate order (Savage-Rumbaugh & Wilkerson, 1978; Wrangham, 1993). In the wild, both bonobos and chimpanzees live in fission—fusion communities ranging from ten to over a hundred individuals (30–80 individuals on average, (Lehmann & Boesch, 2004; IUCN & ICCN, 2012). However, unlike chimpanzees, the social system of bonobos is characterised by a co-dominance between the sexes, which is rare within the primate taxa (Surbeck & Hohmann, 2013). In addition, bonobo communities are tolerant towards each other (Samuni, Langergraber & Surbeck, 2022) and even cooperate (Samuni & Surbeck, 2023) which brought about a new perspective on the discussion of the behaviour of human ancestors (Stanford, 1998; De Waal & Lanting, 2023). Comparative studies of bonobos and chimpanzees using FACS will be able to assess predictions related to these particular socio-ecological variations between species. For example, FACS can aid in testing the association between facial repertoires or mobility with socio-ecological variables, such as group size or complexity (Florkiewicz et al., 2024), and species tolerance (Rincon et al., 2023).

Despite bonobos and chimpanzees differing in behaviour and socio-ecological variables, they are phylogenetically closely related to each other and to humans. Given these particular characteristics, a FACS approach to bonobo facial behaviour may bring new information to the debate on the origins of emotion and communication in humans (Barrett, Gendron & Huang, 2009; Kret, Massen & de Waal, 2022; Majeed, 2022).

Hence, here we present an extension to the ChimpFACS (The Chimpanzee Facial Action Coding System) for bonobos. The current work is intended to be used as an observational coding scheme to measure facial movements in bonobos. Due to chimpanzees and bonobos having very similar facial musculature in terms of number, size, and placement of muscles, and because FACS is based on the facial musculature, the ChimpFACS can likely be used to code facial movements in both species. However, bonobos show some differences from chimpanzees in their facial landmarks (see “Facial morphology in bonobos” section below), which are crucial to identify AUs. Hence, other than the study of the anatomy, it is necessary to confirm and validate that the ChimpFACS is indeed suitable for application to bonobos. In addition, due to behavioural or ecological variation between species, there may also be small differences in the appearance changes of AUs. Hence, having a unique set of video examples of bonobo AUs will aid ChimpFACS certified users in AU identification specifically in bonobos, rather than just in chimpanzees.

The aims of this work are to: (1) define the anatomical and functional plan of the bonobo facial muscles using homologies with the human and the chimpanzee facial musculature; (2) test the application of ChimpFACS and identify examples of the corresponding facial movements of bonobos through spontaneous facial movements analysis and categorisation into AUs, (3) develop an extension to the ChimpFACS manual for bonobos (*i.e.,* the results of the current work) to aid users in identifying AUs in bonobos, and (4) create a standard reference, together with ChimpFACS, for coding AUs in future research on bonobos.

## Methodology

### Ethical statements

This work was approved by the Ethics Advisory Board of Leipzig University (Ref. 2023.04.06_eb_191). All work undertaken for this manuscript was purely observational. Furthermore, some of the videos analysed in this work were collected by FW during a previous unrelated field project (research permit number: MIN.RSIT/SG-RSIT/182/180/029/2023). This field project was granted ethical permission for video recording at the Kokolopori Bonobo Reserve by the Institutional Animal Care and Use Committee at the Faculty of Arts and Sciences at Harvard University, the Ministry of Research of the Democratic Republic of the Congo, and is in line with the ethical guidelines of the Department of Primatology at the Max-Planck-Institute for Evolutionary Anthropology. The field video data collection was conducted non-invasively and adhered to the best practice guidelines for health monitoring and disease control in great ape populations (Gilardi et al., 2015).

### Adapting FACS to bonobos

The methodology used in initial adaptations from the human FACS to other animal species (*i.e.,* chimpanzee (*Pan troglodytes*) and rhesus macaques (*Macaca mulatta*), (Vick et al., 2007; Parr et al., 2010)), employed a three-step process: the first step consisted of examining the facial muscular plan of the target species through dissections; the second step was to perform intramuscular electrical stimulation to verify the link between muscle contractions and appearance changes; finally, in the third step, video analysis of spontaneous behaviour of individuals was undertaken to identify all potential facial movements of the target species.

However, the subsequent FACS adaptations to a new species involved slight changes in this three-step methodology. The intramuscular stimulation was not performed due to lack of availability of individuals, technical issues, and/or associated ethical concerns with an invasive procedure. Furthermore, the validity of a FACS approach based on facial muscle function had been demonstrated already for three species (humans and chimpanzees, Waller et al., 2006, and rhesus macaques, Waller et al., 2008). Dissections were not performed for Barbary (*M. sylvanus*) (Julle-Danière et al., 2015), nor Japanese macaques (*M. fuscata*) (Correia-Caeiro, Holmes & Miyabe-Nishiwaki, 2021) due to lack of significant variation in facial muscles in the genus *Macaca* (Burrows, Waller & Micheletta, 2016). Classic (Miller, 1952) and recent (Burrows et al., 2006; Diogo, Molnar & Wood, 2017; Diogo et al., 2017) dissections have been published on the facial musculature of a large sample of bonobos (*Pan paniscus*, which found no differences between chimpanzee and bonobo facial muscles) with only slight differences with human facial muscles, so additional dissections are not needed for FACS development in this case. Given that chimpanzees already have a dedicated FACS—the ChimpFACS (Vick et al., 2007), we instead can test the application of the ChimpFACS to bonobos.

Thus, in the current work, we perform the third step of a FACS adaptation, in which we analysed videos of spontaneous facial behaviours frame-by-frame to identify examples of independent facial movements in bonobos using the ChimpFACS, along with a list of appearance changes based on facial landmarks comparisons (Figs. S1–S3), and the minimum coding criteria for each movement (see ‘Results’). These movements were then linked to the underlying musculature through functional homology with chimpanzees and humans. Whilst identifying the AU examples, we also noted morphological differences between the appearance changes of the two species. Finally, we looked for potential additional movements in bonobos not included in the ChimpFACS, using the functional homologies of human facial muscles. The work here reported was initially developed as the diploma thesis of one of the co-authors (PK, Kuchenbuch, 2010), and has been here expanded by including a larger sample of individuals, more examples of AUs, and an inter-rater reliability assessment of ChimpFACS application to bonobos.

### Facial morphology in bonobos

Since facial morphology is unique to each species, facial landmarks and other anatomical reference points are important for identifying facial movements when using FACS. These differences are important to be described since some of these may impact the identification of AUs and future FACS coders should become familiar with the details in the facial features of both species. Hence, we compiled this information in Text SI2, including a description of bonobo facial morphology, highlighting key differences between chimpanzees and bonobos, female and male bonobos, and potential individual variations.

### Analysis of bonobo facial movements

The third step for adapting the ChimpFACS for bonobos consisted of analysing video recordings of spontaneous facial movements of bonobos with the aim of (1) identifying the facial movements (AUs and ADs) bonobos can potentially display with the aid of the ChimpFACS, (2) finding at least one clear example of each facial movement in bonobos, and (3) extracting brief video clips to demonstrate these movements (provided in this manuscript as Videos SI). Although ear movements were not included in the ChimpFACS, here these movements were also considered and Ear Action Descriptors (EADs) were described following nomenclature from the MaqFACS (Parr et al., 2010).

A sample of 509 videos (Mean duration ± SD: 477 ± 0.002s) were analysed (by PK and CCC) frame-by-frame, totalling approximately 55 h of videos. The videos were selected according to FACS visibility criteria of the head and face (*e.g.*, lighting, proximity, video quality), and included variable frame rate (29–60 FPS). This sample featured approximately 241 individuals (it was not always possible to identify individuals in the videos) in a variety of populations, both in human care (*i.e.,* housed in zoos, research facilities, and sanctuaries—see Table SI2 in Text SI3, comprising approximately 43 h of video), and from wild sites (wild sites information in Text SI4, comprising approximately 12 h of video). The videos also included a variety of contexts (including potentially affiliative, agonistic, and socially neutral contexts: *e.g.*, grooming, play, aggression, genital rubbing, human interaction, for more information on contexts see Table SI3 in Text SI5). This sample aimed at trying to capture as much diversity of populations, individuals, conditions, and contexts to ensure maximum opportunity for AUs display. However, we did not systematically compare differences between these variables, as this can only be done once the system is developed.

All videos were collected for other ethically approved research projects unrelated to the present work, or sourced from online public databases (*e.g.*, YouTube.com, all with a Creative Commons Licence or approval from the video owners). Still images were extracted from videos in some instances, downloaded from public databases (*e.g.*, Pixabay.com, Unsplash.com), or belonged to other projects by the co-authors (PK, FW, SWT, MS, ML) to illustrate particular facial features or aid in the identification of appearance changes.

We recognise that it is possible that movements displayed in very specific contexts (*e.g.*, birthing) or that are rare may be missing from our video sample. However, if additional movements are found in the future, they can be added to the ChimpFACS Extension for bonobos through the AnimalFACS platform (http://www.animalFACS.com).

### Classification of facial movements into Action Units, Action Descriptors, and Ear Action Descriptors

Similarly to what has been done in previous AnimalFACS, the ChimpFACS Extension for bonobos describes here the facial movements observed in spontaneous facial behaviour of bonobos. Each of the movements observed was classified into AUs/ADs/EADs according to codes used in ChimpFACS, HumanFACS, or MaqFACS, following functional muscular homologies wherever possible. All the AUs found in bonobos are presented in Table S1 along with the corresponding muscles. Additionally, Table S2 lists ADs and EADSs found in bonobos. Detailed description of ADs and EADs in bonobos can be found in Text SI6.

In order to test the application of ChimpFACS to bonobos, we performed inter-rater reliability on a set of additional videos (full description in Text SI7).

### How to use the ChimpFACS Extension for bonobos as a coding scheme

This work aimed to test if the ChimpFACS could be applied to bonobos and thus serves as a *ChimpFACS Extension for bonobos*. It includes only changes to the AU/AD/EADs found in bonobos in comparison to chimpanzees, along with a list of appearance changes that follow each muscular movement on the face of bonobo. Each movement is illustrated by still images and short video-clips included as Videos SI. For each AU, minimum criteria are set for bonobos, in which the presence of specific visible appearance change(s) are a condition required to code an AU.

Despite FACS being based on muscular activity and appearance changes, it is necessary to account for individual differences in permanent morphological facial features present in a neutral face, such as permanent wrinkles, amount of hair, or shape of brows, which may vary between individuals. These individual differences in the neutral face may mislead observers into falsely detecting facial activity when it is not present (*i.e.,* false indicators, Vick et al., 2007). Therefore, the neutral face of each individual should be identified and used as a baseline for coding an AU or AU combinations.

As with all FACS any person interested can become a certified ChimpFACS coder, as due to the objective nature of FACS, no experience with FACS or the target species is needed (*e.g.*, Wathan et al., 2015). However, before taking the ChimpFACS certification test to code bonobos, it is recommended that both the ChimpFACS and this Extension for bonobos are studied together. This includes in-depth understanding of the anatomical information, the appearance changes, and the minimum criteria for both species as described for each AU/AD/EAD (see ChimpFACS (Vick et al., 2007), results section, and Text SI6), and illustrated in the Videos SI. Therefore, the information here reported for bonobos must be used as an additional resource to ChimpFACS, and only after training and certification in ChimpFACS (manual and test available at http://www.AnimalFACS.com). ChimpFACS also includes a glossary with anatomical terms and definitions, which can be consulted if needed for bonobos.

## Results

Since our results are to be used as an extension for ChimpFACS, we report here only differences in appearance changes between chimpanzees and bonobos for each AU, as well as additional movements we observed in bonobos, not included in the ChimpFACS. In Table S1, we compile the previously published information on the presence/absence of AUs and its underlying facial muscles for humans (Ekman, Friesen & Hager, 2002a) and chimpanzees (Vick et al., 2007) in comparison with what we found for bonobos. During the extension of the ChimpFACS for chimpanzees to bonobos, we found 28 facial movements in bonobos: 22 AUs, three ADs, and three EADs (Tables S1 and S2). As in other FACS, most of these Actions may be displayed asymmetrically, although we did not systematically sample this in our examples. The coding of asymmetries in FACS is usually noted by adding L (for left hemiface Action) or R (for right hemiface Action) to the code (*e.g.*, AU16L).

Both the information from Table S1 and the AUs descriptions that follow result from the following points: (a) application of the ChimpFACS to bonobos footage to find examples of each AU, while noting morphological differences in appearance changes; (b) identification of additional movements in bonobos (not previously included in the ChimpFACS); and (c) using ChimpFACS together with the ChimpFACS Extension for bonobos, *i.e.,* the information generated by point (a) and point (b), verify if the AUs from ChimpFACS and its Extension can be reliably coded in bonobos, while improving the description of each AU for bonobos.

Detailed results of the inter-rater coding reliability (overall and for each AU) can be found in Text SI7 and Table S3.

### Action Units

As in the original ChimpFACS, we report each AU found in bonobos, with a numerical code, a descriptive name, and a brief comparison of the anatomical features between humans, chimpanzees, and bonobos. In ChimpFACS, the information is structured in the following way:

**A.** **Proposed muscular basis:** muscle(s) that produces the AU in chimpanzees (Table S1). As the recent dissections indicated bonobos to have all the same muscles, we will omit this section here, although we may comment on the muscle actions if needed.

**B.** **Appearance changes:** this is a list of multiple and redundant cues (*e.g.*, face feature movement of shape change, movement direction, and formation or deepening of wrinkles, in relation to facial landmarks, Figs. S1–S3) that help to identify when an AU occurs. Here, we will comment on any differences and provide additional appearance changes if needed. Video (see Videos SI) and photo examples are also presented illustrating different appearance changes;

**C.** **Minimum criteria** to code an AU: this section details the visible appearance change(s) that, when present, are *sufficient* to code an AU; here we will only present this section for bonobos if it differs from chimpanzees;

**D.** **Subtle differences between AUs:** wherever necessary, a comparison of similar AUs that can be confused or that share some appearance changes, if different from what was reported in ChimpFACS.

### Upper face Action Units

#### AU1+2—Brow Raiser

The raising of the browridge is brought about by the contraction of the frontalis muscle in chimpanzees, which is also the case here. The description of appearance changes for AU1+2 in the ChimpFACS can be applied to bonobos without major modifications: the brows are pulled up in their entirety, the wrinkles on the forehead deepen and the skin under the browridge becomes visible. The human FACS AU1—Inner Brow Raiser and AU2—Outer Brow Raiser do not seem to act independently in chimpanzees nor bonobos. In all of the AU1+2 observed in bonobos, the inner and outer sections of the brow were raised together (*e.g.*, Figs. S5–S6, Videos S1–S6).

AU1+2 may be more difficult to detect in some bonobos than in chimpanzees, as the browridge is less prominent, so that less skin will be moving over it. In addition, like in many other AUs, the black skin colour of bonobos may render it more difficult to see the changes in lines and wrinkles.

#### AU41—Glabella Lowerer

Humans can produce AU4—Brow Lowerer by the contraction of three different muscles: procerus, depressor supercilii, and corrugator supercilii. The procerus pulls the medial end of the eyebrow downward, the depressor supercilii pulls the eyebrow and the skin above the orbit downward, and the corrugator supercilii pulls the eyebrows towards the midline and downwards. No such independent movement was described for chimpanzees in the ChimpFACS, and it was not seen either in bonobos in the initial ChimpFACS adaptation study (Kuchenbuch, 2010). In both works, AU4 was only observed with AU9—Nose Wrinkler. However, this single movement of lowering the brows has been described in the literature in bonobos (often with an emotional label). De Waal (1988) and Gaspar (2006) both use the word “frown” for certain appearances of bonobo faces. One photograph in de Waal’s article (Fig. 1A in De Waal, 1988) shows an individual with the inner brow area appearing to be drawn inward and downward, but there is no neutral picture to compare this appearance to, which makes it difficult to identify an AU4 with certainty. The face of one of the individuals observed in Kuchenbuch’s (2010) study has an appearance similar to human frowning in its neutral state, but no individual showed a singular movement in accord with the minimum criterion for an AU4, *i.e.,* downwards movement of the brow region. In the non-FACS literature, this “frowning” is generally described as a medial and downward movement of the inner brow area, just like it is observed in humans. Using the human FACS approach, this would likely include at the very least the lowering of the medial part of the browridge caused by the procerus muscle, *i.e.,* an AU41—Glabella Lowerer. However, as explained above, caution is needed to not code a false indicator due to permanent features of an individual’s face in a neutral state. AU41—Glabella Lowerer has also been identified in another primate taxa, the *Macaca* genus and thus included in the MaqFACS and its extensions (Parr et al., 2010; Julle-Danière et al., 2015; Clark et al., 2020; Correia-Caeiro, Holmes & Miyabe-Nishiwaki, 2021).

In the larger dataset with bonobos used in the current work, we clearly identified several examples of an independent movement in which the browridge was lowered. However, it is unclear if corrugation is widely present (only observed in one individual once). Hence, despite the three muscles producing AU4 in humans being present in bonobos (Diogo, Molnar & Wood, 2017; Diogo et al., 2017), and in bonobos these muscles producing some of the same appearance changes seen in humans, without corrugation present as one of the appearance changes of this movement, AU4—Brow Lowerer cannot be coded. Corrugation of the brows, *i.e.,* brows coming closer together and creating wrinkles on the glabella, has not yet been found in any other AnimalFACS, and thus seems to be unique to humans. Therefore, in bonobos, AU41—Glabella Lowerer is coded for downwards browridge movements, which we describe next with the corresponding sections A–D (*e.g.*, Videos S7–S10).

The glabella area (*i.e.,* area between the brows) differs slightly between humans and bonobos. While in humans the glabella is a flat area of smooth skin between the brows, in bonobos the glabella area usually has permanent wrinkles, and may sit deeper than the rest of the browridge or the salient arches of the browridge. In humans, the glabella contrasts with the surrounding areas due to being a (usually) hairless area between two strips of hair, while in bonobos either the whole browridge may be covered in hair or may be fully naked (see Fig. S4 for examples of variation in this feature), which may make identifying AU41 more challenging in bonobos.

**A.** **Proposed muscular basis:** procerus.

**B. Appearance changes:**

1. The browridge moves downwards, with either the browridge moving down or the skin of the browridge sliding down and inwards.
2. The eye aperture may be narrowed.
3. In more intense movements, the underbrow region may become less visible, the eye cover fold may disappear from view, and the root of the nose may also be covered.
4. If hair is present above or on the browridge, it will move downwards.
5. In very intense movements, the root of the nose might be completely covered with the glabella.
6. In some individuals, wrinkles may appear or may be deepened on the glabella and root of the nose.

**C.** **Minimum criteria**: downwards movement of the browridge, glabella, or brow arches.

**D.** **Subtle differences between AUs:** even though AU1+2 and AU41 act in opposite directions, in humans they can be coded simultaneously, impacting each other’s appearance changes and creating new appearance changes. In contrast, in bonobos they are mutually exclusive, *i.e.,* they cannot be coded simultaneously, and hence do not share any appearance changes. In bonobos, either an AU1+2 is acting whenever the browridge goes upwards from neutral, or an AU41 is acting whenever the browridge goes downwards from neutral. However, these two movements are sometimes observed in succession, in which AU1+2 is immediately followed by AU41 (or vice-versa), sometimes without a clear temporal return to neutral (*e.g.*, Videos S11–S13). In addition, the return of AU41 to neutral might be difficult to distinguish from a weak AU1+2, and the return of AU1+2 to neutral might be confused with a weak AU41. However, the release of AU1+2 should not be coded as AU41, and vice-versa. To define when to code one or the other, comparison with the neutral browridge for each individual may be necessary, as well as frame-by-frame analysis of the succession of movements (Fig. S7). In still frames, weak occurrences of this movement might be hard to code in absence of a neutral picture of the same individual.

#### AU5—Upper Lid Raiser

The action of this AU opens the eyes widely by raising the upper lid beyond the neutral open position. The raising of the upper lid is brought about by the contraction of the levator palpebrae superioris muscle in humans and chimpanzees, but this muscle was not described for bonobos as the extra-ocular musculature was not examined in the recent dissections (Diogo et al., 2017). In some species, the orbicularis oculi muscle may also act to further increase the opening of the eye.

In any case, this AU was reported to be present in chimpanzees and bonobos, with the same basic appearance in both species (Kuchenbuch, 2010). The eye aperture is widened, exposing more of the upper part of the eyeball, while part of the upper eyelid itself is concealed under the browridge. In some bonobos, as the browridge may be smaller, the eyes are less overshadowed than in chimpanzees, so that the upper eyelid may be more visible. This makes this AU slightly easier to discern in individuals with smaller browridges. If AU1+2 is acting, this may also expose more of the eye region and facilitate the identification of AU5 (Fig. S8). The influence of head angle and eye movements on the identification of AU5 is as important in bonobos as in chimpanzees; changes in head angle can lead the observer to erroneously assume that the upper eyelid has been raised, and the same can happen if the individual is looking up. Figure S9 illustrates these effects, showing a combination of AU5, looking up, and an upward head movement.

However, both in the ChimpFACS and the initial work done in bonobos, AU5 was not observed in isolation, and all examples are accompanied by either AU41 or eye/head movements. Similarly, we did not identify this movement in our current sample as an independent movement (*e.g.*, temporally distinct from other AUs). Hence, this is another AU that may be hard to code depending on individuals or video conditions.

#### AU6—Cheek Raiser and AU7—Lid Tightener

In chimpanzees, AU6—Cheek Raiser is produced by the contraction of the orbicularis oculi (pars orbitalis) muscle, and it has the same muscular basis in bonobos. This muscle action pulls the skin from around the eyes towards the centre of the eye. Narrowing of the eye aperture, as described for chimpanzees, is also seen in bonobos. The medial section of the browridge can be seen to be pulled downwards, which also happens in stronger actions in chimpanzees. In some movements, beyond the lowering of the brows, the skin of the browridge and frontal region is seen sliding over the browridge and downwards towards the eyes (*e.g.*, Videos S14–S15). AU6 can be easily identified in bonobos from these cues alone. However, it has less conspicuous appearance changes on the corners of the eyes than in chimpanzees: certain wrinkles lying laterally of the eye region seem to be shallower in bonobos than in chimpanzees, which makes it harder to see any deepening of these or increase of bagging beneath the eyes, as described in the ChimpFACS. The black skin colour may also make these appearance changes harder to see. The possibility to use wrinkles and bagging as hints for the action of AU6 is therefore more limited than in chimpanzees. Like in chimpanzees, this action in bonobos seems to be more spread around the whole eye area, in a concentric movement (*e.g.*, Video S16).

It is important to note that we could not find an example of AU6 in isolation in bonobos, but only in conjunction with other AUs, such as AU45/43, AU9 (see sections below for description of these AUs, Fig. S10), or AU41. This was the case for both the initial work in applying FACS to bonobos (Kuchenbuch, 2010) and in the current larger sample. However, there is a slight temporal difference in AU6 and the other AUs, and thus we consider it as an independent AU for bonobos, similar to what was determined for chimpanzees. In humans, likely due to anatomical differences (*e.g.*, fat deposits on the zygomatic bone that form the human-like cheeks), AU6 can be coded as an independent action.

In bonobos, we also detected another movement that often accompanies AU6: AU7 - Lid Tightener, in which the lower eyelid is raised or bulged (Fig. S11). In humans AU7 can be coded as an independent AU and is produced by the other portion of the orbicularis oculi (pars palpebralis) muscle. However, in chimpanzees this action was not described, as the authors thought several factors could influence the reliability of detecting this movement (Vick et al., 2007). In bonobos, AU7 may likewise be hard to detect reliably due to the difficulty in clearly visualising the eyelid, and was only observed twice independently and as a very subtle movement in the analysis of our whole video dataset (Videos S17–S18). Hence, we recommend to code this movement only in optimal conditions of visibility of the eye area.

As both AU6—Cheek Raiser and AU7—Lid Tightener seem to be slightly different in bonobos compared to chimpanzees, we include the appearance changes for both of these below.

**A.** **Proposed muscular basis for AU6—Cheek Raiser:** orbicularis oculi (pars orbitalis).

**B.** **Appearance changes for AU6—Cheek Raiser:**

1. The skin around the eye is pulled towards the centre of the eye, including skin from browridge, IOT (infra-orbital triangle, Figs. S1–S2), nose and temples.
2. Skin wrinkles around the eye deepen, and new wrinkles may form around the eye.
3. The skin of the IOT is pulled towards the eye, but may not decrease in size.
4. Deepens the infraorbital furrow.
5. Even if eye closure is not present, it usually narrows eye aperture.
6. It was only observed in conjunction with other AUs, such as AU41, AU43/45, and AU9 (see below for further AUs descriptions).
7. When eye closure or AU7 are present, it can make the eyelids appear compressed and bulging.

**C.** **Minimum criteria for AU6—Cheek Raiser**: concentric movement of skin globally around the eyes being pulled inwards toward the centre of the eye.

**D.** **Subtle differences between AUs for AU6—Cheek Raiser:** as AU6 was not observed alone, this makes it harder to distinguish from other AUs. Nonetheless, there has to be at least a temporal distinction with other AUs appearance changes. AU6 may be confused with AU41, AU9, or AU43/45. However, with AU41 and AU9, movement will not be global, but with AU41 will be seen coming from the browridge downwards only, and with AU9 there will be only movement coming from the nose and IOT area upwards. With AU43/45 alone, there is some movement around the eye, but its area is less extensive and less globalised.

**A.** **Proposed muscular basis for AU7—Lid Tightener:** orbicularis oculi (pars palpebralis).

**B.** **Appearance changes for AU7—Lid Tightener:**

1. The eyelid is tightened and moves upwards covering more of the eyeball. There might be some movement also towards the corner of the eye.
2. It was only observed in the lower eyelid.
3. It narrows eye aperture.
4. It may change shape slightly from a curved U line to a more straight or arched line.
5. The skin right underneath below the eyelid may move upwards, but not beyond the IOT.
6. Lower eyelid furrow may become more apparent.

**C.** **Minimum criteria for AU7—Lid Tightener**: lower eyelid is pushed upwards covering more of the eyeball.

**D.** **Subtle differences between AUs for AU7—Lid Tightener:** AU7 may be confused with AU6, as in AU7 there is also movement in the IOT and eyelid. However, in AU7, this movement is not concentric, and more just a push of the eyelid upwards or towards the inner corner of the eyes, whilst in AU6 the movement is concentric towards the centre of the eye. Furthermore, we observed AU6 in both eyelids, whilst we only observed AU7 in the lower eyelid.

### AU43—Eye Closure and AU45—Blink

Action Units 43 and 45 both describe the closing of the eyelids. But as the name indicates, AU43 is coded when the eyes are closed for more than half a second, and AU45 if they are closed for half a second or less (*e.g.*, Video S19). Both AUs are caused not by the contraction, but by the relaxation of a muscle, *i.e.,* the levator palpebrae superioris muscle, in humans and chimpanzees. In bonobos as in chimpanzees, these AUs reduce the eye aperture while exposing more of the upper eyelid. The smaller browridge has a slightly positive effect on the visibility of these AUs, while the black colour of the eyelids (with the exception of individuals with bright eyelids) has a slightly negative effect. The overall visibility of this movement is thus similar to chimpanzees.

### Lower face Action Units

#### AU9—Nose Wrinkler

In humans, the levator labii superioris alaeque nasi muscle wrinkles the nose, producing AU9—Nose Wrinkler, but in chimpanzees, this AU may recruit an additional muscle, the procerus muscle. Like in humans, in chimpanzees, this AU pulls the nostrils upwards and medially, causing wrinkling at the root of the nose, while the medial portion of the browridge is pulled downwards. The lateral portion of the nasolabial furrow is deepened in chimpanzees.

Both muscles are present in bonobos (Miller, 1952; Diogo, Molnar & Wood, 2017; Diogo et al., 2017). The AU9 has similar appearance changes in this species (Fig. S12, Videos S20–S22), but because the bonobo nose is broader than in chimpanzees, the medial movement of the nostrils is less pronounced. The deepening of the nasolabial furrow described in chimpanzees is more difficult to discern in bonobos, and the same can be said of the wrinkling at the root of the nose. Therefore, this AU is easier to recognise in bonobos by the slight upward movement of the nose, which can be seen more clearly in profile than in frontal view. In addition, it seems that smaller movements produce more appearance changes around the nostril area, pulling the nostrils upwards, whilst in more intense movements the nostril, side of nose, upper lip, and glabella may have visible changes.

#### AU10—Upper Lip Raiser

AU10—Upper Lip Raiser refers to the upper lip being pulled upwards. This action may reveal parts of the upper teeth and gums. The muscle responsible for this movement in chimpanzees is the levator labii superioris muscle. This AU looks very similar in bonobos; a noticeable shortening of the distance between the nose and the upper lip is the best cue for recognition in bonobos as it is in chimpanzees. However, the upper lip seems to thicken less than in chimpanzees, although this may be due to many occurrences of AU10 being accompanied by AU12—Lip Corner Puller, which stretches the lips horizontally. The lighter lip colouration in some bonobos creates a higher contrast area, which may facilitate the identification of this movement (*e.g.*, Figs. S13–S15, Videos S23–S26).

#### AU12—Lip Corner Puller

This AU pulls the corners of the lips upwards and laterally towards the ears (*e.g.*, Figs. S16–S17, Videos S27–S29). The underlying muscle action is the contraction of the zygomaticus major muscle in chimpanzees. The mouth is elongated in the horizontal plane; in chimpanzees, the stretching of the top lip caused by this AU makes the vertical wrinkles between mouth and nose less visible. This appearance change can also be seen in bonobos. However, the appearance change that describes the deepening of the semi-circular mouth corner furrows into wrinkles mentioned in the ChimpFACS is not as straightforward in bonobos to detect. In many bonobos, the hair comes down and medially further than in many chimpanzees. The area where wrinkling is said to occur in chimpanzees when AU12 is acting is often covered by hair in bonobos, so that wrinkling in the mouth corner can then not be used as a reliable cue to recognise the action of AU12.

However, the bright lips make the elongation of the mouth into a crescent shape easier to see in bonobos, which compensates for when the lip corners are not visible. As in chimpanzees, a slight upward curving of the mouth corners is often present in the neutral face (AU0) of bonobos (Fig. S18). The degree of this curvature is individually different. The presence of AU12 alone is therefore often hard to discern. Thus, it is especially important for the correct coding of AU12 to consider the AU0 of the individual or for the actual movement of the lip corners to be detected.

#### AU16—Lower Lip Depressor

This AU pulls the lower lip towards the mental region. The inner lip area, the lower teeth and gums may be revealed, the lips are always parted (AU25), and the lower lip may bulge (but not fall anteriorly). The responsible muscle is the depressor labii inferioris muscle in chimpanzees, and this muscle is also present in bonobos (Miller, 1952; Diogo, Molnar & Wood, 2017; Diogo et al., 2017). We found no differences in AU16 between bonobos and chimpanzees, hence the ChimpFACS appearance changes can be used without any modification for bonobos (*e.g.*, Figs. S19–S20, Videos S30–S32).

#### AU160—Lower Lip Relax

The Lower Lip Relax - AU160 describes the strong relaxation of the lower lip to the point that it falls forward and hangs loose. This AU is not observed in humans, due to the small size and low flexibility of the human lower lip. It was however described for chimpanzees (Vick et al., 2007), orangutans (Correia-Caeiro et al., 2013), and gorillas (Correia-Caeiro et al., 2025), since these species have a very large, thick, and highly mobile lower lip. In bonobos, AU160 is also observed, and it presents similar appearance changes to the other apes (*e.g.*, Fig. S21, Videos S33–S35).

#### AU17—Chin Raiser

In humans, the main appearance change of this AU is an upward movement of the chin and the lower lip, causing the lower lip to protrude. In humans and chimpanzees, it is caused by the contraction of the mentalis muscle. This muscle is not mentioned in Miller’s work on bonobo facial musculature (Miller, 1952), but it was identified in the more recent dissections (Diogo et al., 2017). In bonobos, there is a very obvious presence of AU17 illustrated by the several examples included in Kuchenbuch’s work (2010) and here included. The description of this AU in chimpanzees can be applied to bonobos without major modifications (*e.g.*, Figs. S22–S23, Videos S36–S39). This AU is also clearly visible in bonobos because of the contrasting bright lip colour, which makes the lip and the mental area appearance changes much more visible than in chimpanzees.

#### AU18—Lip Pucker

The action of this AU in humans pushes the lips slightly forwards, the mouth opening is de-elongated becoming smaller and rounder, and wrinkles appear on the upper and lower lip (Ekman, Friesen & Hager, 2002a). AU18 is produced by the incisivii labii superioris and inferioris muscles to produce the typical “kiss/pucker” mouth shape in humans (Ekman, Friesen & Hager, 2002a; Hur, 2018). In the ChimpFACS, the presence of these muscles and the AU are noted as unclear. The published dissections do not seem to agree regarding the nomenclature, description, and inclusion of these muscles in NHP (Diogo et al., 2017), but it is unclear if this is because it was not dissected or it could not be found, or due to some technical issues. However, a recent work targeting these muscles was published with one chimpanzee sampled (Iwanaga et al., 2021), in which this muscle was clearly identified, at least in the lower lip, but with a different proposed function than the one proposed in the human FACS (Ekman, Friesen & Hager, 2002a). Hence, more studies on these muscles are needed to clarify their presence and function in human and NHP.

Due to the yet tentative muscular basis in bonobos, Kuchenbuch (2010) included only a suggestion for an AU18, consisting of several appearance changes: a clearly visible elongation of the lips with a pointed centre, but with the lips pulled flat in the vertical plane, and without outwards flaring. These appearance changes, albeit with slight differences, were suggested to be like the human FACS AU18 - Lip Pucker (Ekman, Friesen & Hager, 2002a). However, when examining these picture and video examples from the work of Kuchenbuch (2010) (Fig. S24), there seems to be more similar appearance changes from a weak AU22—Lip Funneler (see below for description), without the extensive outwards flaring of the lips (but still there is a slight flare in both videos), than an AU18, according to the human FACS. However, both videos also contain an AU18 (see below for still frames of AU18). The AU18 in the human FACS does not require lip protrusion (just mouth de-elongation and pursing of the lips), whilst the AU22 in the human FACS always requires the lips to be projected forwards (Ekman, Friesen & Hager, 2002a). The human FACS concedes that AU22 can happen without AU25, if AU17 is acting to push the lower lip upwards towards the upper lip and thus keeping the lips together. Furthermore, in other ape species, such as orangutans (Correia-Caeiro et al., 2013), similar movements to these ones observed in Kuchenbuch’s work were classified as AU22 in the OrangFACS, in which the projected lips can take two forms: funnelled or flattened. In the OrangFACS, AU18 was also included, but with the appearance changes of pursing the lips medially and the appearance of wrinkles in both lips, with none or minimal protrusion of the lips. As such, here we complement both movements (AU18 and AU22) with further video and picture examples, and opted for following the appearance changes of ChimpFACS and OrangFACS for AU18 and AU22.

In the current work, we identified several instances of AU18 in bonobos (*e.g.*, Fig. S25, Videos S40–S42), and we further add that AU18 can be produced with or without wrinkling, and can be coded whenever the lip corners are drawn medially, even if no puckering is observed. As this movement was not described in the ChimpFACS, we include below its detailed description for bonobos.

**A.** **Proposed muscular basis:** orbicularis oris and incisivii labii.

**B.** **Appearance changes:**

1. The lip corners move towards the mouth midline or the lips are pushed towards the mouth midline.
2. Some medial bulging of the lips may be observed, but there is no outwards flaring of the lips.
3. Wrinkling may be observed or the existent wrinkles become more conspicuous, particularly on the upper lip.
4. The mouth area may appear to become narrower as it appears compressed medially. In frontal view, the lip corners become visible.
5. The lip corners may appear to be slightly pointing upwards due to the movement of the upper lip being squeezed towards the mouth midline.
6. If the mouth is open, fewer teeth may become visible. The sharp angle of the lip corners becomes rounder.
7. Skin and hair accompany the lip corner movement towards the medial area of the mouth.
8. It can be observed as a unilateral movement on one side of the mouth (AU18L or AU18R, Videos S40). It can also occur only on one of the lips (top lip: AU18T or bottom lip: AU18B, Videos S41).
9. In low intensity movements, only the lip corners move slightly forward, without wrinkling or mouth shape change. Hence, in low intensity movements, AU18 may not be visible from a full frontal view if the lip corners are not visible (*e.g.*,  Videos S42).

**C.** **Minimum criteria**: the lip corners are pushed medially or the lips are drawn together medially.

**D.** **Subtle differences between AUs:** AU18 and AU12 are mutually exclusive movements as they move the lip corners in opposing directions. However, AU18 might be confused with AU12 returning to neutral and vice-versa. Therefore, identifying the neutral position of the lip corners in a particular individual is important to distinguish these two movements and the release of the opposing movement. In addition, with AU18 the lip corners may appear to be slightly pointing upwards (Fig. S25), which is a false indicator for AU12. However, in AU18 the lip corners move forward, not towards the ears as it happens in AU12.

AU18 can also be confused with AU22, since both movements may present some degree of lip protrusion and wrinkles on both lips. However, the main difference is the shape of the lips, where in AU18 the protrusion is due to the bunching up of the lips in the medial area of the mouth, whilst in AU22 the protrusion is due to one or both lips flaring out and taking a funnel or flattened shape.

Caution is needed when coding AU18 in bonobos, as the neutral position of the lip corners is hard to determine in many individuals. Hence, as a minimum criterion to code this AU, movement of the lip corners or the lip itself moving medially must be observed.

#### AU22—Lip Funneler

In humans, chimpanzees, and bonobos, AU22—Lip Funneler is produced by the orbicularis oris muscle to project the lips medially forwards and outwards in a typical funnelled or flattened shape. In chimpanzees and bonobos, due to the larger size of the lips in comparison to humans, AU22 is a more conspicuous movement and can have more variation of how the lips are positioned during this movement. In bonobos and chimpanzees, this AU can result in circular parting of the lips in the middle, resulting in the exposure of the inner lip area and often the separation of the lips (which needs the additional code AU25 - Lips Parted). The mental region is pulled slightly upwards. The vertical lines on the upper lip deepen during this action in both species. Pronounced actions of this AU are well visible in bonobos from a frontal view because of the bright lips: a flesh-coloured circle appears when the lips are funnelled; but it is easy to detect movement from a side view as well, as the lips are projected forwards (Figs. S26–S29, Videos S43–S48).

In the ChimpFACS, action of AU22 for the top lip only was described, but in our sample of bonobo videos, funnelling of the upper lip alone was not observed. Instead, we observed different intensities of AU22 in each lip, so it is possible that bonobos also have the ability of producing AU22 in only one lip (coded as AU22T for the top lip or AU22B for the bottom lip). In addition, we observed a higher mobility and flexibility of the lower lip in bonobos, beyond the funnel/flattened shape described for a typical AU22 described for humans or chimpanzees. In humans the lower lip is short, and other than the AU22, does not have a lot of flexibility to curl outwards or extend extensively. In bonobos, the lower lip is much larger than in humans, being often used to hold and manipulate objects or food. This differentiated lower lip morphology allows the production of movements that extend and elongate the lower lip outwards, which we also include as appearance changes for AU22 (*e.g.*, Fig. S29, Video S48).

#### AU24—Lip Presser

This AU presses both lips together, causing lip bulging, particularly in the upper lip, and can only be coded if both lips are together. This action is produced by the orbicularis oris muscle in both chimpanzees and bonobos. The appearance changes of the AU are the same in chimpanzees and bonobos, except there seems to be more bulging and less wrinkling on the lips in bonobos than in chimpanzees (*e.g.*, Figs. S30–S31, Videos S49–S51). The visibility in bonobos may be slightly better than in chimpanzees because of the contrasting lip colour.

#### AU25—Lips Part

Action Unit 25 parts the lips. Part of the teeth, gums and the inner mucosal lip area may be revealed. These appearance changes were found to be the same in bonobos as in chimpanzees (*e.g.*, Videos S52–S53). Other AUs often are acting to part the lips (*e.g.*, AU10 and AU16); AU25 is then coded additionally.

#### AU26—Jaw Drop

This AU lowers the mandible by relaxation of the jaw musculature. The lips may part, and if they do, a small space between the upper and lower teeth is visible. The appearance changes in bonobos are the same as in chimpanzees, and the same cues can be used for identification (*e.g.*, Fig. S32–S33, Videos S52–S53).

#### AU27—Mouth Stretch

AU27 actively pulls down the mandible, which results in a wide mouth opening. The mouth is elongated vertically. Visually, the only difference between AU26 and AU27 is the degree of separation of the teeth, where in AU26 there is a small space and in AU27 a large space between the teeth. Although AU27 describes a larger degree of mouth opening in relation to AU26, AU27 is mutually exclusive to AU26, as different muscles are involved. Due to the degree of mouth opening always separating the lips in AU27, it is coded as AU25+AU27. In addition, although this is mostly not visible, there is more muscular tension around the mouth and lips which are being stretched, hence the skin might appear smoother around the lips and lip corners. However, this only becomes visible in high intensity AU27. High intensity AU26 and low intensity AU27 may be hard to distinguish and only by continued visualisation of several examples can this discrimination be achieved by coders. All these appearance changes apply equally to chimpanzees and bonobos (*e.g.*, Figs. S34–S351, Videos S54–S55).

#### AU28—Lips Suck

In chimpanzees and bonobos, AU28—Lips Suck have similar appearance changes, with the main difference being the brighter colour of the lips in bonobos, which makes this movement more conspicuous (*e.g.*, Figs. S36–S37, Videos S56–S58). This movement in bonobos seems often to accompany AU24—Lip Presser, and this AU24+AU28 combination is described as possible in humans, but it was not reported for chimpanzees. However, the subtle differences reported in the ChimpFACS between AU24 and AU28 also apply to bonobos, with bulging of the upper lip being the minimum criterion to code AU24. If both lip insertion into mouth and bulging are present, AU24+AU28 should be coded together.

#### AU38—Nostril Dilator and AU39—Nostril Compressor

In humans the nostrils are widened with AU38—Nostril Dilator, or constricted with AU39—Nostril Compressor, by the action of the nasalis muscle. In the ChimpFACS this movement is stated as not observed and it was not observed either in Kuchenbuch’s work (2010), but we found these in the current video sample. Bonobos present a much larger and more developed nasal region than humans and to some extent chimpanzees, and a more connected nose to the upper lip. These differences in facial morphology affect the appearance changes to code AUs. These movements can be subtle movements or very quick movements, and hence harder to detect than other AUs.

**A.** **Proposed muscular basis:** nasalis.

**B. Appearance changes:**

1. In AU38, the nostril increases in size (*e.g.*, Video S59), and in AU39 decreases in size (*e.g.*, Video S60). In AU38/AU39 the nostrils usually change shape or at least the nostril wings will present some movement.
2. The skin next to the nose and on the nose shield as a whole might move, enlarging, contracting, projecting forward, or flattening, accompanying each of the respective movements.

**C.** **Minimum criteria**: the nostrils are widened in AU38 and narrowed in AU39.

**D. Subtle differences between AUs:** caution regarding appearance change 2 is needed, as movement of the skin around the nose might be due to other AUs pulling the skin globally in the mouth region (*e.g.*, AU10, AU16). Hence, AU38/AU39 are coded only if the minimum criterion for each AU is present, *i.e.,* if the movement is clearly originating in the nostrils or nasal shield. Additionally, these nose AUs may be difficult to detect depending on the angle and distance of the individual from the camera. Changes in head position might appear to change the nostril shape. Therefore, if there are head movements that can change the observed nostril shape or the nasal shield shape, the nose AUs should be coded only when movement is detected in the nostrils or the nasal shield.

In addition, AU38/AU39 might be hard to code in a side view if the nostrils are not fully visible.

AU38 is mutually exclusive to AU39 as these are opposite movements.

## Discussion

The ChimpFACS Extension for bonobos is the latest AnimalFACS to be developed in a series of FACS adaptations for animals that currently includes nine systems and four extensions, covering 21 species (see Table SI4 in Text SI8 for full list). Since FACS allows precise measure and quantification of facial movements, we will now be able to answer a wide range of previously unexplored questions. For example, which species are more facially mobile (*e.g.*, number or duration of AUs, combination of AUs), or which species use their facial movements flexibly or in line with intentionality criteria (Waller, Correia-Caeiro & Davila-Ross, 2015; Scheider et al., 2016; Townsend et al., 2016; Mielke et al., 2022; Correia-Caeiro & Liebal, 2023; Rincon et al., 2023). Another area virtually unexplored so far is the ontogenetic development of facial behaviour across NHP (Bard et al., 2014; Liebal, Schneider & Errson-Lembeck, 2019), which now with all apes having their own FACS adaptation, becomes a possibility.

For our first aim, the anatomical comparisons based on previous published dissections (Burrows et al., 2006; Diogo, Molnar & Wood, 2017; Diogo et al., 2017; Miller, 1952) and the examination of bonobos facial morphology demonstrated that the faces of chimpanzees and bonobos are very similar, and hence, it was expected that a direct application of ChimpFACS was likely possible. Such approach was taken for some species of the genus *Macaca*, in which due to lack of significant differences in facial musculature for a FACS adaptation (Burrows, Waller & Micheletta, 2016), the MaqFACS originally developed for rhesus macaques was extended to Barbary (Julle-Danière et al., 2015), crested (Clark et al., 2020), and Japanese macaques (Correia-Caeiro, Holmes & Miyabe-Nishiwaki, 2021).

Indeed the inter-reliability process and the application of ChimpFACS to analyse bonobo videos demonstrated that ChimpFACS can be applied for bonobos, fulfilling our second aim. In the process of testing the ChimpFACS application for bonobos, we also documented a few additional AUs, not previously included in the ChimpFACS, but included in the human FACS namely AU41—Glabella Lowerer and AU7—Lid Tightener, or in the MaqFACS, namely the three EADs: EAD1—Ears Forward, EAD2—Ears Elevator, and EAD3—Ears Flattener. AU4 was not included in the ChimpFACS due to absence of corrugation in the glabella (Vick et al., 2007) which is a minimum criterion to code this AU. However, AU41—Glabella Lowerer (*i.e.,* lowering the browridge but without corrugation) was later described in the MaqFACS and respective extensions for the genus *Macaca* and also observed in bonobos. Furthermore, in previous studies, brow lowering has been reported as significantly more frequent in bonobos than chimpanzees (Bard, Gaspar & Vick, 2011), so it is possible that clear examples of this movement might not have been detected during the ChimpFACS development. Even though we observed several clear examples of AU41 in bonobos, inter-rater reliability was still low overall for this AU (due to variability between coders when coding this AU), which may be due to difficulty discriminating between AU1+2 and AU41 when these movements are continuous, or between AU41 and release of AU1+2. Hence, caution is needed when coding AU41, which is likely only reliable in more optimal viewing conditions.

The current work also found clear examples of AU7—Lid Tightener. This AU was not included in the ChimpFACS due to lack of contrast around the eye area and difficulty in having a clear line of sight to the eyelids, eyes, and surrounding skin areas. AU5—Upper Lid Raiser and AU6—Cheek Raiser were included in the ChimpFACS, but discussed as difficult AUs to code in chimpanzees for the same reasons as the lack of an AU7, while in bonobos the enhanced eye region colour contrast and the smaller browridge facilitated the identification of these three movements. Perhaps due to advancements in technology for recording equipment nowadays compared to 20 years ago, we were able to identify clear examples of each of these AUs in our videos. However, as highlighted by the inter-rater reliability process, these AUs still seemed infrequent or difficult to detect, and not all of them were reliably coded between all coders. Other AUs, such as AU1+2—Brow Raiser or AU28—Lips Suck, achieved higher average reliability between all coders in the final coding round, but presented variation between coders. While it is not clear why this variation happened, as we still know very little about the learning cognitive mechanisms that take place during FACS training (*i.e.,* changes in facial behaviour perception from global to local features), it is possible that this may be related to number of certifications in AnimalFACS or experience of coding FACS in general for each coder. Additional factors such as minimum intensity of movement to code an AU, particularly if features are not clearly visible, or degree of familiarity with the neutral face of an individual may also lead to more disagreement in specific AUs. Further examples of these AUs and additional descriptions were added to try to improve the coding process for future coders using ChimpFACS and its Extension for bonobos. Hence, these AUs and all other AUs with lower inter-rater reliability should only be coded cautiously and if possible in ideal visibility situations for FACS coding (*e.g.*, light, proximity, high definition recording equipment, low movement).

It is possible that the additional movements reported here for bonobos are also present in chimpanzees, but they may have not been included in the original ChimpFACS for several reasons, such as for example, lower quality of video recordings or smaller/less diverse database of videos to sample the AUs from. Hence, the ChimpFACS Extension for bonobos might be useful when coding chimpanzees. It may also be the case that the movements observed in bonobos but not in chimpanzees may just be less frequent in the latter and hence larger/more varied samples of videos are needed. However, we did not measure frequency or duration of AUs in this work, so future research on chimpanzee and bonobo facial behaviour using FACS is needed to confirm this. Finally, despite both species being overall facially similar, small differences such as slightly higher contrast around eyes/mouth areas and decreased browridge may have facilitated the detection of appearance changes in bonobos as opposed to chimpanzees. Likewise, these more suitable features for AUs visibility may also be responsible for the perception that bonobos are more facially mobile. To better understand all these factors, cross-species comparison studies using FACS are needed in the future.

An important addition to this ChimpFACS Extension for bonobos that was not included in the ChimpFACS and will certainly be useful to be applied in chimpanzees, are the EADs. We found three ear movements in bonobos, similar to what has been found before for several macaque species (Parr et al., 2010; Correia-Caeiro, Holmes & Miyabe-Nishiwaki, 2021). Although in bonobos EADs seem to not be as frequent or as conspicuous as in macaques, we found clear examples of these movements. As the ChimpFACS was initially developed to compare facial movements between humans and chimpanzees, ear movements were not needed, and these were only considered as an addition to an AnimalFACS in the MaqFACS. Hence, the EADs here described, as well as other AUs and ADs not included in the ChimpFACS, can likely retrospectively be applied to chimpanzees, supplementing the number of AUs that can be applied to this species as well.

Some comparisons of facial behaviour in chimpanzees and bonobos seem to conclude that bonobos are more facially mobile than chimpanzees (De Waal, 1988; Bard, Gaspar & Vick, 2011). In De Waal’s (1988) study, higher frequency of facial behaviours was displayed by bonobos in sexual behaviour, and by chimpanzees in aggression contexts, but with bonobos overall having higher number of unique facial behaviours (12) *vs.* chimpanzees (6). In Bard, Gaspar & Vick (2011), both quantitative and qualitative measures were taken, with bonobos displaying more movements with particular AUs. Supporting De Waal (1988) and Wrangham (1993) also suggested that bonobos have less conspicuous agonistic behaviour compared to other primate species, which could suggest fewer facial movements overall. Alternatively, it could be that higher number or complexity of facial behaviours in bonobos compared to chimpanzees, is used to maintain their more tolerant (Samuni, Langergraber & Surbeck, 2022) and cooperative (Samuni & Surbeck, 2023) societies, leading to lower agonistic interactions. However, these studies did not apply FACS or lacked inter-rater reliability, and so the Extension for bonobos can build on this previous work to better investigate the complexities of facial behaviour in bonobos in agonistic contexts and in relation to the species despotic level. In another primate taxon in which the MaqFACS and its extensions were applied, it was found that the more tolerant species of the genus (*e.g.*, crested macaques) display a more diverse facial behaviour when compared to less tolerant species (*e.g.*, Barbary and rhesus macaques) (Rincon et al., 2023). These studies in different species seem to support facial behaviour complexity (measured with FACS) to be associated with tolerance.

In a more recent study that used bonobo play faces *vs* aggressive faces to distinguish between contexts, showed contradictory evidence to the studies mentioned above, in which male-male aggression in bonobos was higher than in chimpanzees (Mouginot et al., 2024). However, as the studies above, this study classified facial behaviour holistically, which provides less information about potential differences in facial behaviour, such as for example that play faces might contain different AUs (Waller et al., 2013). Further comparisons of facial behaviour use between both *Pan* species are therefore likely needed to better understand these seemingly conflicting results. One potential reason for these reported species differences in facial mobility might be that these studies (De Waal, 1988; Wrangham, 1993; Mouginot et al., 2024) did not specifically examine differences in facial movement in both species with FACS, and till date no truly comparative and systematic quantification of facial movements in the *Pan* genus has been done.

In our third goal, we described 22 AUs in bonobos, which indicates a lower potential for facial mobility than in humans (30 AUs), but higher than in chimpanzees (17 AUs), or other apes, namely orangutans (17 AUs) and gibbons (20 AUs). However, these results do not necessarily suggest that bonobos are more facially mobile than chimpanzees, even though a larger number of AUs has been described in the current work. As explained above, other factors are likely responsible for the higher number of facial movements reported here (see also Correia-Caeiro et al., 2025 for further discussion on this). Furthermore, it is important to highlight that the FACS only outlines the potentiality for movement, not its actual occurrence, intensity, complexity, or contextual use. To fully understand these aspects, FACS must be applied in controlled observational or empirical studies. The ChimpFACS and the Extension for bonobos thus serve as a standard reference for coding facial behaviour in both these species, which will enable future detailed and objective comparisons between species, encompassing not only the occurrence and quantity of AUs, but also their complexity (*e.g.*, number of AUs in each facial behaviour) and function (*i.e.,* in which contexts the AUs are produced).

In addition to the questions mentioned above, the ChimpFACS Extension for bonobos can be used to further explore a vast number of research questions including population differences (*e.g.*, cultural use of facial behaviours), individual differences (*e.g.*, influence of sex or personality on facial behaviours), and human influence (*e.g.*, wild *vs.* human managed), both at a species, or if in combination with other AnimalFACS, at a comparative level (*e.g.*, individual differences in bonobos *vs* chimpanzees).

The ChimpFACS Extension for bonobos provides a valuable opportunity to advance our understanding on the social behaviour of this less studied ape through one of the most important but tendentially understudied modalities of communication in this species. This coding scheme has potential to offer novel insights into bonobo social communication and emotional expression, and facilitates comparative studies with humans and other primates species.

## Supplemental Information

10.7717/peerj.19484/supp-1 Supplemental Information 1 Comparison of facial landmarks of humans and bonobos Colour of labels simply for better visualisation purposes. CC licensed images from Pixabay users tarasnesterenko1 –human, and hwtz11 - bonobo.

10.7717/peerj.19484/supp-2 Supplemental Information 2 Comparison of facial landmarks of chimpanzees and bonobos Both species present the same facial landmarks used in FACS appearance changes descriptions. Noticeable differences are on the nasal shield shape/size (see also Figure S3), and upper eyelids and lips edge colouration. Colour of labels simply for better visualisation purposes. CC licensed images from Pixabay users Pixel-mixer - chimpanzee and hwtz11 - bonobo.

10.7717/peerj.19484/supp-3 Supplemental Information 3 Focused landmarks for the nasal region of bonobos The nasal shield in bonobos presents more visually complex landmarks than other facial regions - here separated from Figure S2 for better visualisation. Colour of labels simply for better visualisation purposes. CC licensed image from Pixabay user hwtz11.

10.7717/peerj.19484/supp-4 Supplemental Information 4 Variation in hair coverage in frontal region, browridge shape, and facial colouration between individuals Some individuals have almost no hair on their frontal region, others have short sparse hair, and others have long, thick hair parted in half and sticking to the sides. The hair density and length affects visibility of the frontal region, browridge, and ears, which in turn may make AUs or EADs identification more challenging. Bonobos also seem to present variation in browridge shape, size, and saliency. Individuals with longer hair on the frontal region may obscure some of the appearance changes for browridge AUs (AU1+2 and AU41). There is also considerable variation in the nasal shield shape and size, presenting more or less wrinkles on the nasal bridge. Finally, the facial colouration also shows variation, with some individuals presenting all dark faces, some all dark faces with lips bright pink/cream colour, whilst in others, the whole mouth region is lighter in colour. This variation in features is important to keep in mind during FACS coding as it may affect the coding of AUs. These images are still frames from videos by PK (B, I), ML (E, F), Friends of Bonobos/Lola Ya Bonobo (D, G), and photographs by FW/Kokolopori Bonobo Research Project (A, C, H, J-L).

10.7717/peerj.19484/supp-5 Supplemental Information 5 Example of AU1+2 - Brow Raiser Left**:**neutral browridge. Right: AU1+2 - Brow Raiser (indicated by the yellow arrow). Still frames from video by PK.

10.7717/peerj.19484/supp-6 Supplemental Information 6 Example of AU1+2 - Brow Raiser Left: neutral browridge. Right: AU1+2 - Brow Raiser. Still frames from video by PK.

10.7717/peerj.19484/supp-7 Supplemental Information 7 Example of AU1+2 - Brow Raiser and AU41 - Glabella Lowerer Left: AU1+2 - Brow Raiser. Centre: Neutral browridge. Left: AU41 - Glabella Lowerer. Still frames from video by ML.

10.7717/peerj.19484/supp-8 Supplemental Information 8 Example of AU5 - Upper Lid Raiser Left: neutral eye region. Right: AU5 - Upper Lid Raiser. Other AUs are present (including AU1+2). Still frames from video by PK.

10.7717/peerj.19484/supp-9 Supplemental Information 9 Example of AU5 - Upper Lid Raiser Left: neutral eye region. Right: AU5 - Upper Lid Raiser. Other AUs are present (including AD63 - Eyes Up and AD53 - Head Up). Still frames from video by PK.

10.7717/peerj.19484/supp-10 Supplemental Information 10 Example of AU6 - Cheek Raiser Left: neutral under eye area; Right: AU6 - Cheek Raiser. Other AUs present. Still frames from video by PK.

10.7717/peerj.19484/supp-11 Supplemental Information 11 Example of AU7 - Lid Tightener Left: neutral under eye area; Right: AU7 - Lid Tightener (more apparent on right eyelid than on the left, in which the lower eyelid is straighter and the eyeball is visibly more covered). Other AUs present. Still frames from S11 Video by Friends of Bonobos/Lola Ya Bonobo.

10.7717/peerj.19484/supp-12 Supplemental Information 12 Example of AU9 - Nose Wrinkler For both top and bottom frames: Left: neutral nose area; Right: AU9 - Nose Wrinkler. Other AUs present. Still frames from video by PK.

10.7717/peerj.19484/supp-13 Supplemental Information 13 Example of AU10 - Upper Lip Raiser Other AUs present. Picture by ML.

10.7717/peerj.19484/supp-14 Supplemental Information 14 Example of AU10 - Upper Lip Raiser Other AUs present. Still frames from video by PK.

10.7717/peerj.19484/supp-15 Supplemental Information 15 Example of AU10 - Upper Lip Raiser Other AUs present. Still frames from video by PK.

10.7717/peerj.19484/supp-16 Supplemental Information 16 Example of AU12 - Lip Corner Puller For both top and bottom frames: Left: neutral mouth corner area; Right: AU12 - Lip Corner Puller. Other AUs present. Still frames from video by PK.

10.7717/peerj.19484/supp-17 Supplemental Information 17 Example of AU12 - Lip Corner Puller Left: neutral mouth corner area; Right: AU12 - Lip Corner Puller. Other AUs present. Still frames from video by PK.

10.7717/peerj.19484/supp-18 Supplemental Information 18 False indicator for AU12 - Lip Corner Puller Slight upward curving in the neutral faces (AU0) of several individuals. Still frames from video by PK.

10.7717/peerj.19484/supp-19 Supplemental Information 19 Several examples of AU16 - Lower Lip Depressor Other AUs present. Still frames from video by PK.

10.7717/peerj.19484/supp-20 Supplemental Information 20 Examples of AU16 - Lower Lip Depressor Right: AU16R - Right Lower Lip Depressor. Left: AU16L - Left Lower Lip Depressor. Other AUs present. Pictures by FW/Kokolopori Bonobo Research Project.

10.7717/peerj.19484/supp-21 Supplemental Information 21 Examples of AU160 - Lower Lip Relax Other AUs present. Top left: picture by ML, top right, and bottom left and right: pictures by PK.

10.7717/peerj.19484/supp-22 Supplemental Information 22 Example of AU17 - Chin Raiser Left: neutral mental region; Right: AU17 - Chin Raiser. Other AUs present. Still frames from videos by PK.

10.7717/peerj.19484/supp-23 Supplemental Information 23 Example of AU17 - Chin Raiser Left: neutral mental region; Right: AU17 - Chin Raiser. Other AUs present. Still frames from videos by PK.

10.7717/peerj.19484/supp-24 Supplemental Information 24 Examples of AU22 - Lip Funneler previously suggested to be AU18 - Lip Pucker in Kuchenbuch, 2010 Left: note the slight outward flaring of the lower lip; Right: note the slight outward flaring of the top lip. Due to the projection of the lips and the slight flaring outwards of at least one lip, in the present work, both examples are here classified as AU22 - Lip Funneler (see next section for AU22 description). Still frames from video by PK.

10.7717/peerj.19484/supp-25 Supplemental Information 25 Examples of AU18 - Lip Pucker The lip corners are pushed medially and wrinkling of the upper lip is observed. No outwards flare of the lips is visible. Other AUs present. Top: Still frames from video by PK with AU18. Bottom: Pictures by PK.

10.7717/peerj.19484/supp-26 Supplemental Information 26 Example of AU22 - Lip Funneler with the typical funnel shape Still frames from video by PK.

10.7717/peerj.19484/supp-27 Supplemental Information 27 Example of AU22 - Lip Funneler with the typical funnel shape Still frames from video by PK.

10.7717/peerj.19484/supp-28 Supplemental Information 28 Examples of AU22 - Lip Funneler Top Left: AU22 + AU17, with AU17 action pushing the lower lip together with the upper lip. Top Centre: AU22 + AU27. Top Right: AU22 without AU25 (AU17 is likely present closing the lips together). Bottom: AU22 + AU25. Note the flare of at least one of the lips in all examples, presenting a typical flattened shape. Still frames and pictures by PK.

10.7717/peerj.19484/supp-29 Supplemental Information 29 Examples of AU22 –Lip Funneler without the typical funnel or flattened shape, but with lip extension Other AUs present. Left: still frame from video by PK, Right: still frame from video by Friends of Bonobos/Lola Ya Bonobo.

10.7717/peerj.19484/supp-30 Supplemental Information 30 Example of AU24 - Lip Presser Pictures by PK.

10.7717/peerj.19484/supp-31 Supplemental Information 31 Example of AU24 - Lip Presser Picture by FW/Kokolopori Bonobo Research Project.

10.7717/peerj.19484/supp-32 Supplemental Information 32 Example of AU26 - Jaw Drop Pictures by PK.

10.7717/peerj.19484/supp-33 Supplemental Information 33 Example of AU26 –Jaw Drop Picture by PK.

10.7717/peerj.19484/supp-34 Supplemental Information 34 Example of AU27 - Mouth Stretch Other AUs present. Still frames from video by PK.

10.7717/peerj.19484/supp-35 Supplemental Information 35 Example of AU27 - Mouth Stretch Other AUs present. Pictures by FW/Kokolopori Bonobo Research Project.

10.7717/peerj.19484/supp-36 Supplemental Information 36 Example of AU28 - Lips Suck Other AUs present. Still frames from video by PK.

10.7717/peerj.19484/supp-37 Supplemental Information 37 Example of AU28 - Lips Suck Other AUs present. Still frames from video by PK.

10.7717/peerj.19484/supp-38 Supplemental Information 38 Comparison between FACS Action Units (AU) for humans (Ekman, Friesen & Hager, 2002) , chimpanzees (Vick et al., 2007) , and bonobos according to the underlying musculature ✓ - observed, x - not observed. Cells highlighted in grey are actions observed in bonobos.

10.7717/peerj.19484/supp-39 Supplemental Information 39 Comparison between FACS Action Descriptors (AD) for humans (Ekman, Friesen & Hager, 2002) , chimpanzees (Vick et al., 2007) and bonobos ✓ - observed, x - not observed. Cells highlighted in grey were observed in bonobos.

10.7717/peerj.19484/supp-40 Supplemental Information 40 Mean Wexler’s index (Wexler, 1972) (1) and independent coding agreement for each AU, AD and EAD in the two coding rounds NA denotes instances where both coders agreed that a particular Action was not present in any of the clips.

10.7717/peerj.19484/supp-41 Supplemental Information 41 Supplemental Information

## Additional Information and Declarations

### Field Study Permissions

The following information was supplied relating to field study approvals (i.e., approving body and any reference numbers): Ethical permission to collect video data at the Kokolopori Bonobo Reserve was granted by the Institutional Animal Care and Use Committee at the Faculty of Arts and Sciences at Harvard University, the Ministry of Research of the Democratic Republic of the Congo and is in line with the ethical guidelines of the Department of Primatology at the Max-Planck-Institute for Evolutionary Anthropology. The field video data collection was conducted non-invasively and adhered to the best practice guidelines for health monitoring and disease control in great ape populations (Gilardi et al., 2015).

### Data Deposition

The following information was supplied regarding data availability: The data is available in the figshare videos: Correia-Caeiro, Catia (2025). AU1+2. Videos S1–S6. figshare. Media. https://doi.org/10.6084/m9.figshare.29044685.v1
Correia-Caeiro, Catia (2025). AU6. Videos S14–S16. figshare. Media. https://doi.org/10.6084/m9.figshare.29044721.v1
Correia-Caeiro, Catia (2025). AU7. Videos S17–S18. figshare. Media. https://doi.org/10.6084/m9.figshare.29044727.v1
Correia-Caeiro, Catia (2025). AU9. Videos S20–S22. figshare. Media. https://doi.org/10.6084/m9.figshare.29044739.v1
Correia-Caeiro, Catia (2025). AU10. Videos S23–S26. figshare. Media. https://doi.org/10.6084/m9.figshare.29044745.v1
Correia-Caeiro, Catia (2025). AU12. Videos S27–S29. figshare. Media.
https://doi.org/10.6084/m9.figshare.29044751.v1
Correia-Caeiro, Catia (2025). AU16. Videos S30–S32. figshare. Media. https://doi.org/10.6084/m9.figshare.29044754.v1
Correia-Caeiro, Catia (2025). AU17. Videos S36–S39. figshare. Media. https://doi.org/10.6084/m9.figshare.29044760.v1
Correia-Caeiro, Catia (2025). AU18. Videos S40–S42. figshare. Media. https://doi.org/10.6084/m9.figshare.29044763.v1
Correia-Caeiro, Catia (2025). AD19. Videos S61. figshare. Media. https://doi.org/10.6084/m9.figshare.29044793.v1
Correia-Caeiro, Catia (2025). AU22. Videos S43–S48. figshare. Media. https://doi.org/10.6084/m9.figshare.29044769.v1
Correia-Caeiro, Catia (2025). AU24. Videos S49–S51. figshare. Media. https://doi.org/10.6084/m9.figshare.29044775.v1
Correia-Caeiro, Catia (2025). AU25+26. Videos S52–S53. figshare. Media. https://doi.org/10.6084/m9.figshare.29044778.v1
Correia-Caeiro, Catia (2025). AU27. Videos S54–S55. figshare. Media. https://doi.org/10.6084/m9.figshare.29044781.v1
Correia-Caeiro, Catia (2025). AU28. Videos S56–S58. figshare. Media. https://doi.org/10.6084/m9.figshare.29044784.v1
Correia-Caeiro, Catia (2025). AD30. Videos S62–S63. figshare. Media. https://doi.org/10.6084/m9.figshare.29044796.v1
Correia-Caeiro, Catia (2025). AD32. Videos S64. figshare. Media. https://doi.org/10.6084/m9.figshare.29044799.v1
Correia-Caeiro, Catia (2025). AU38 & AU39. Videos S59–S60. figshare. Media. https://doi.org/10.6084/m9.figshare.29044790.v1
Correia-Caeiro, Catia (2025). AU41. Videos S7–S13. figshare. Media. https://doi.org/10.6084/m9.figshare.29044697.v1
Correia-Caeiro, Catia (2025). AU45. Video S19. figshare. Media. https://doi.org/10.6084/m9.figshare.29044736.v1
Correia-Caeiro, Catia (2025). AU160. Videos S33–S35. figshare. Media. https://doi.org/10.6084/m9.figshare.29044757.v1
Correia-Caeiro, Catia (2025). EADs. Videos S65–S68. figshare. Media. https://doi.org/10.6084/m9.figshare.29044802.v1
Correia-Caeiro, Catia (2025). Head and Eye ADs. Videos S69–S70. figshare. Media. https://doi.org/10.6084/m9.figshare.29044805.v1.
