# Supplementary figures and images for: Adapting the facial action coding system for chimpanzees (Pan troglodytes) to bonobos (Pan paniscus): the ChimpFACS extension for bonobos
Source: PeerJ. 2025 Jun 13;13:e19484. doi: 10.7717/peerj.19484 (PMC12169169; doi:10.7717/peerj.19484)

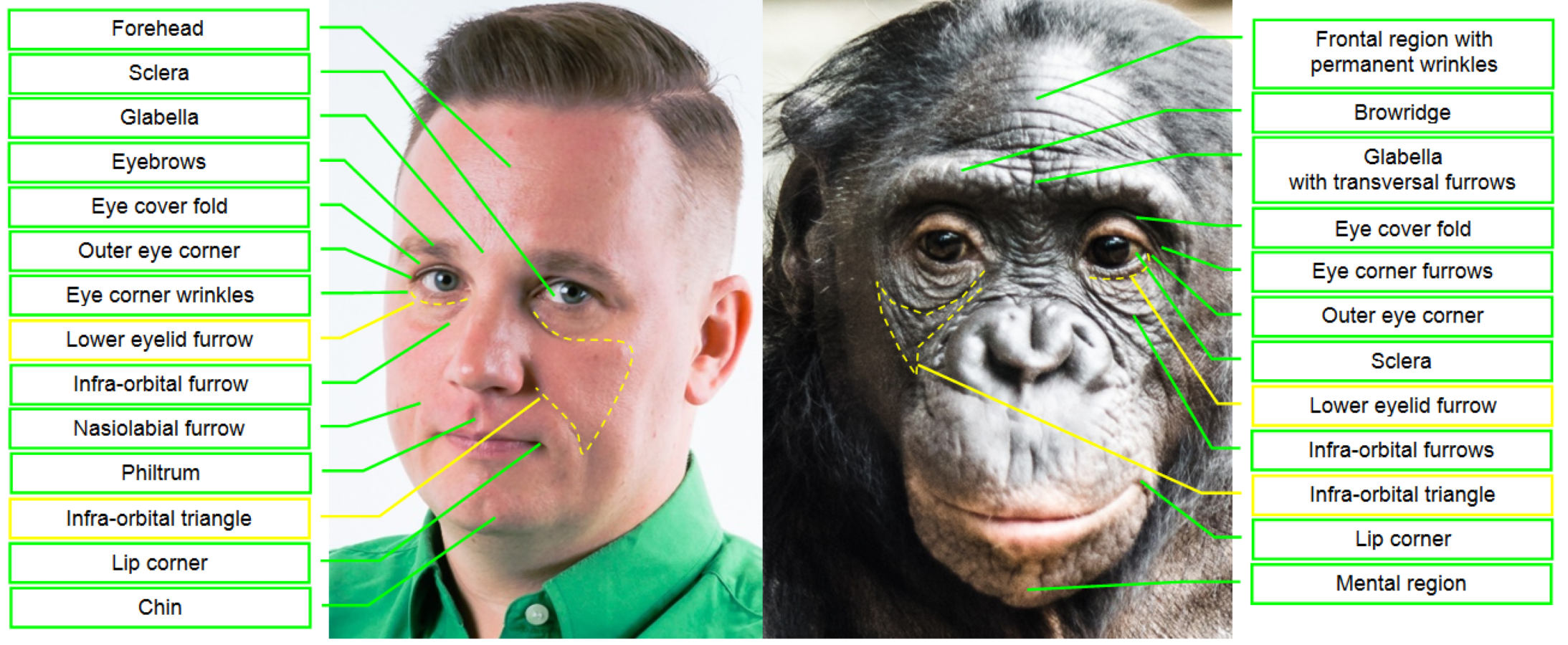

Supplement: Supplemental Information 1 — Colour of labels simply for better visualisation purposes. CC licensed images from Pixabay users tarasnesterenko1 –human, and hwtz11 - bonobo. [file peerj-13-19484-s001.png]

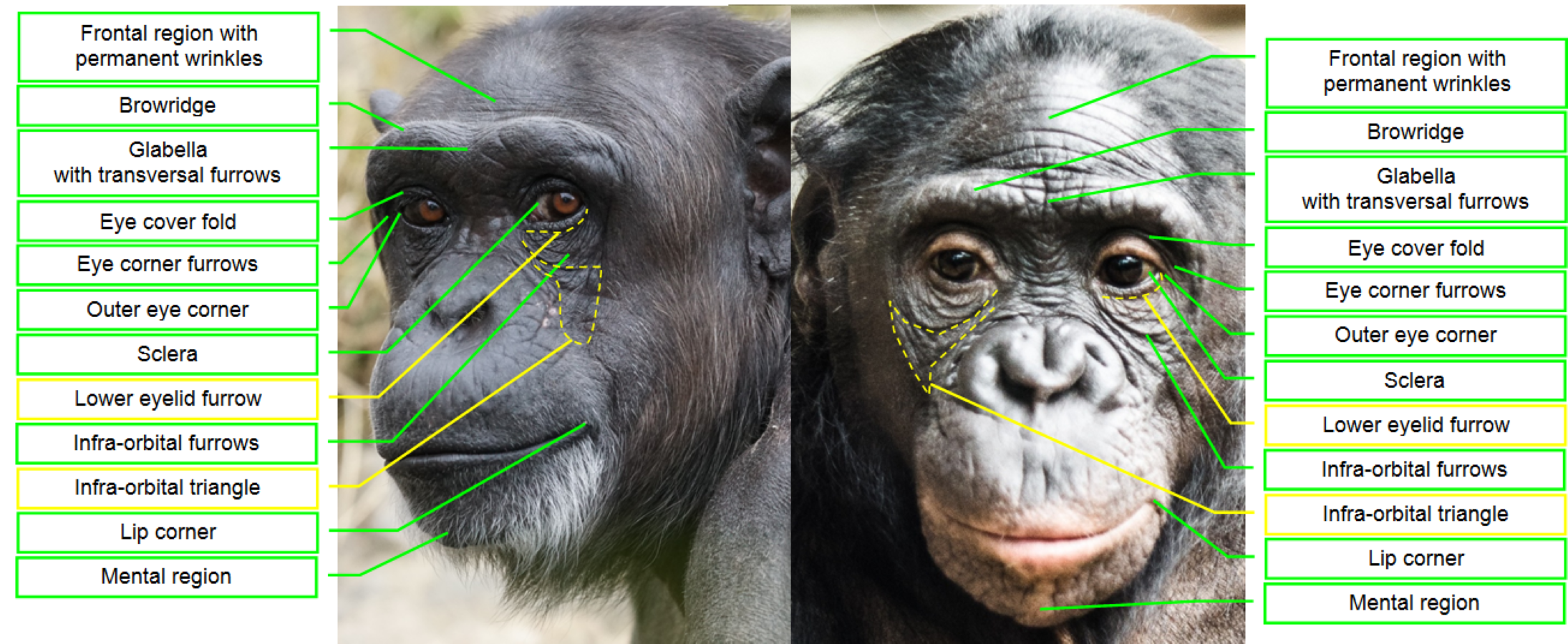

Supplement: Supplemental Information 2 — Both species present the same facial landmarks used in FACS appearance changes descriptions. Noticeable differences are on the nasal shield shape/size (see also Figure S3), and upper eyelids and lips edge colouration. Colour of labels simply for better visualisation purposes. CC licensed images from Pixabay users Pixel-mixer - chimpanzee and hwtz11 - bonobo. [file peerj-13-19484-s002.png]

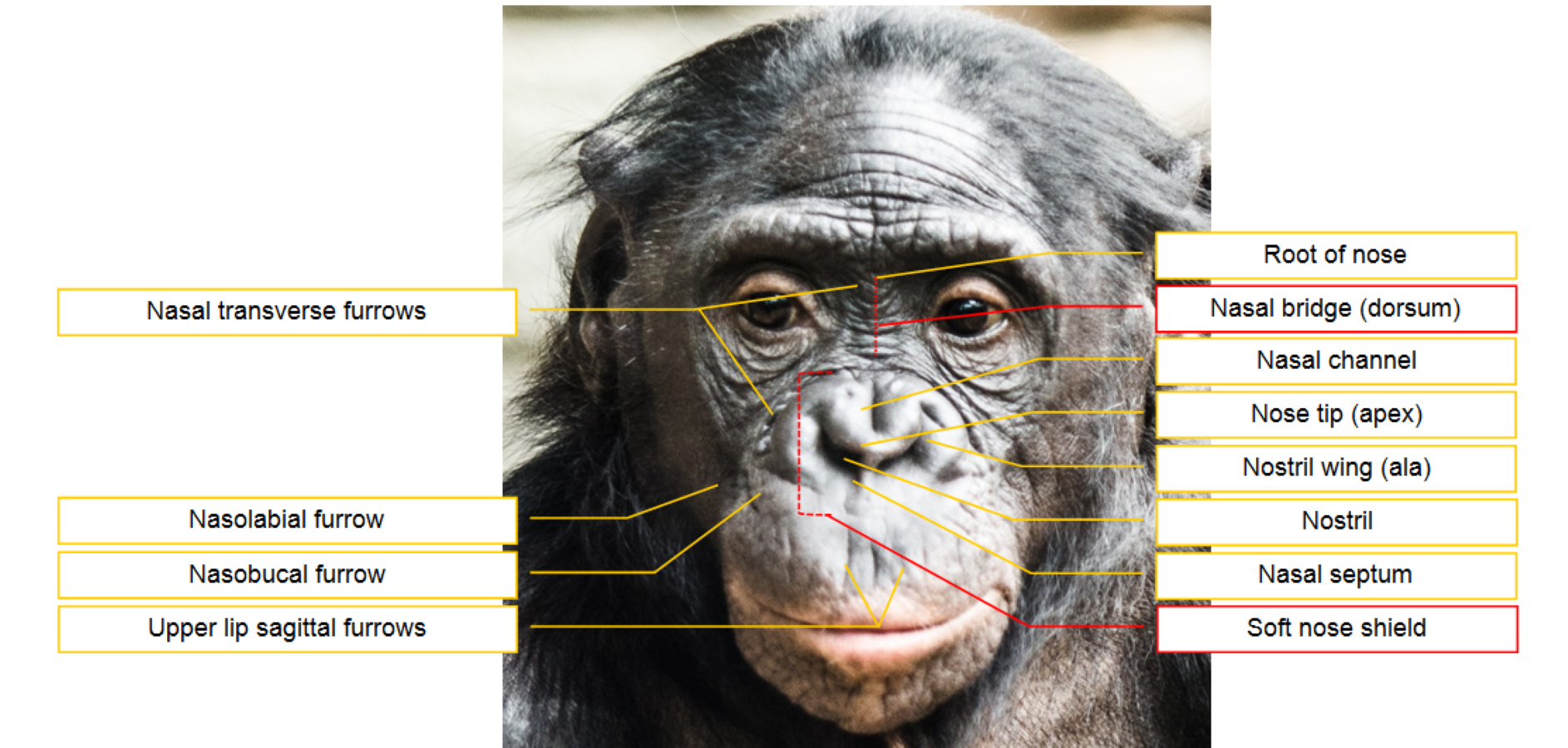

Supplement: Supplemental Information 3 — The nasal shield in bonobos presents more visually complex landmarks than other facial regions - here separated from Figure S2 for better visualisation. Colour of labels simply for better visualisation purposes. CC licensed image from Pixabay user hwtz11. [file peerj-13-19484-s003.png]

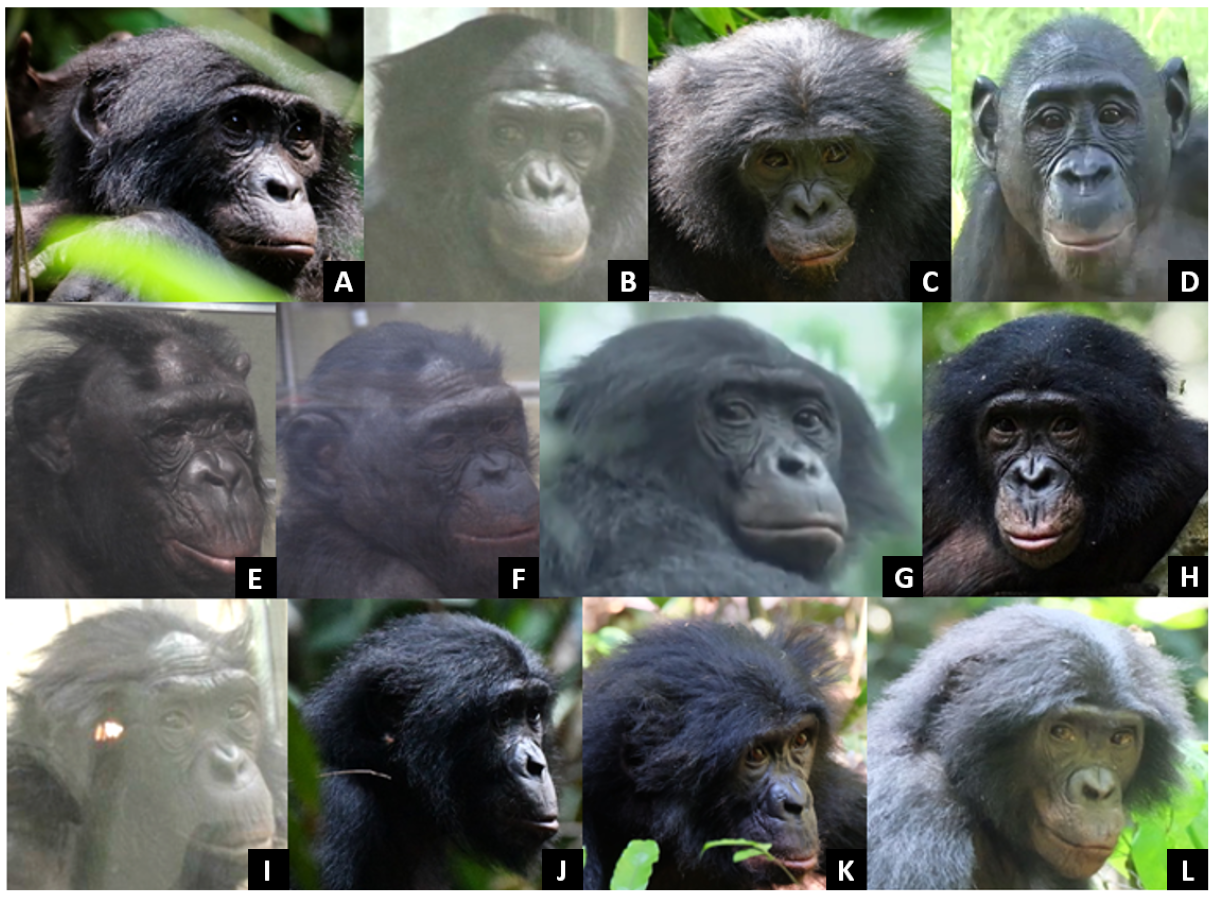

Supplement: Supplemental Information 4 — Some individuals have almost no hair on their frontal region, others have short sparse hair, and others have long, thick hair parted in half and sticking to the sides. The hair density and length affects visibility of the frontal region, browridge, and ears, which in turn may make AUs or EADs identification more challenging. Bonobos also seem to present variation in browridge shape, size, and saliency. Individuals with longer hair on the frontal region may obscure some of the appearance changes for browridge AUs (AU1+2 and AU41). There is also considerable variation in the nasal shield shape and size, presenting more or less wrinkles on the nasal bridge. Finally, the facial colouration also shows variation, with some individuals presenting all dark faces, some all dark faces with lips bright pink/cream colour, whilst in others, the whole mouth region is lighter in colour. This variation in features is important to keep in mind during FACS coding as it may affect the coding of AUs. These images are still frames from videos by PK (B, I), ML (E, F), Friends of Bonobos/Lola Ya Bonobo (D, G), and photographs by FW/Kokolopori Bonobo Research Project (A, C, H, J-L). [file peerj-13-19484-s004.png]

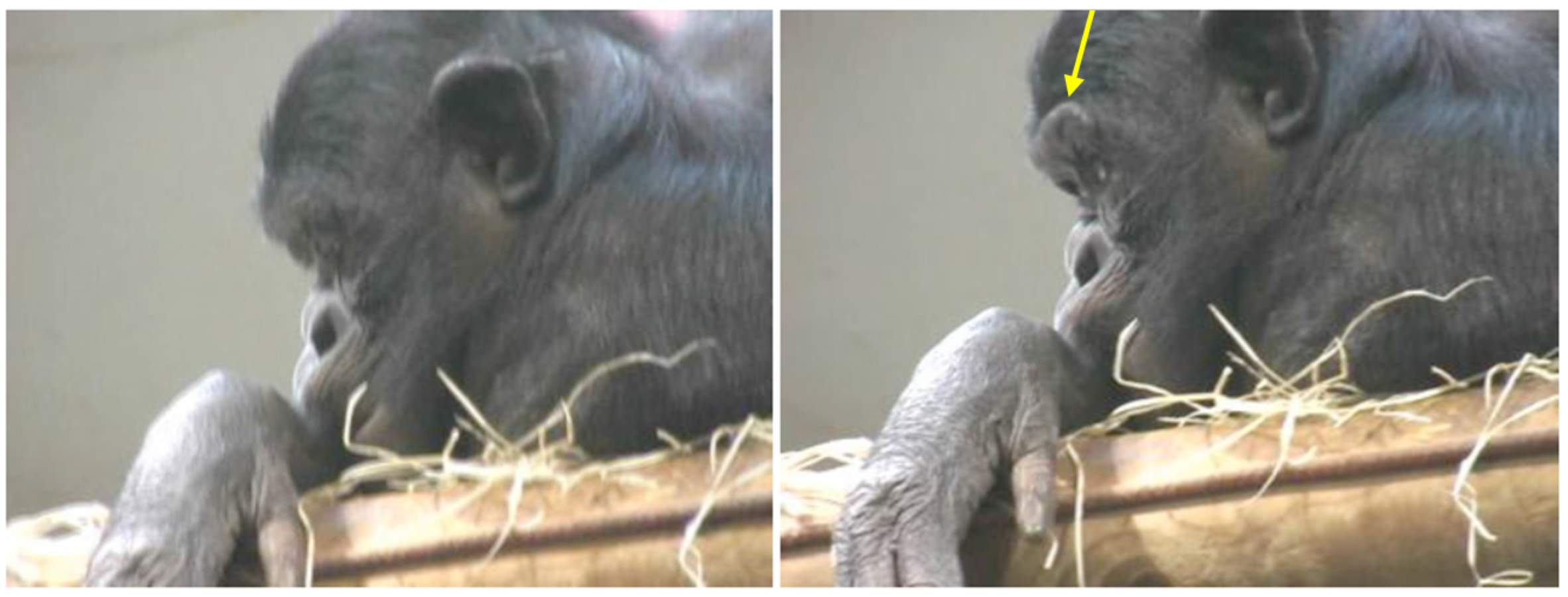

Supplement: Supplemental Information 5 — Left:neutral browridge. Right: AU1+2 - Brow Raiser (indicated by the yellow arrow). Still frames from video by PK. [file peerj-13-19484-s005.png]

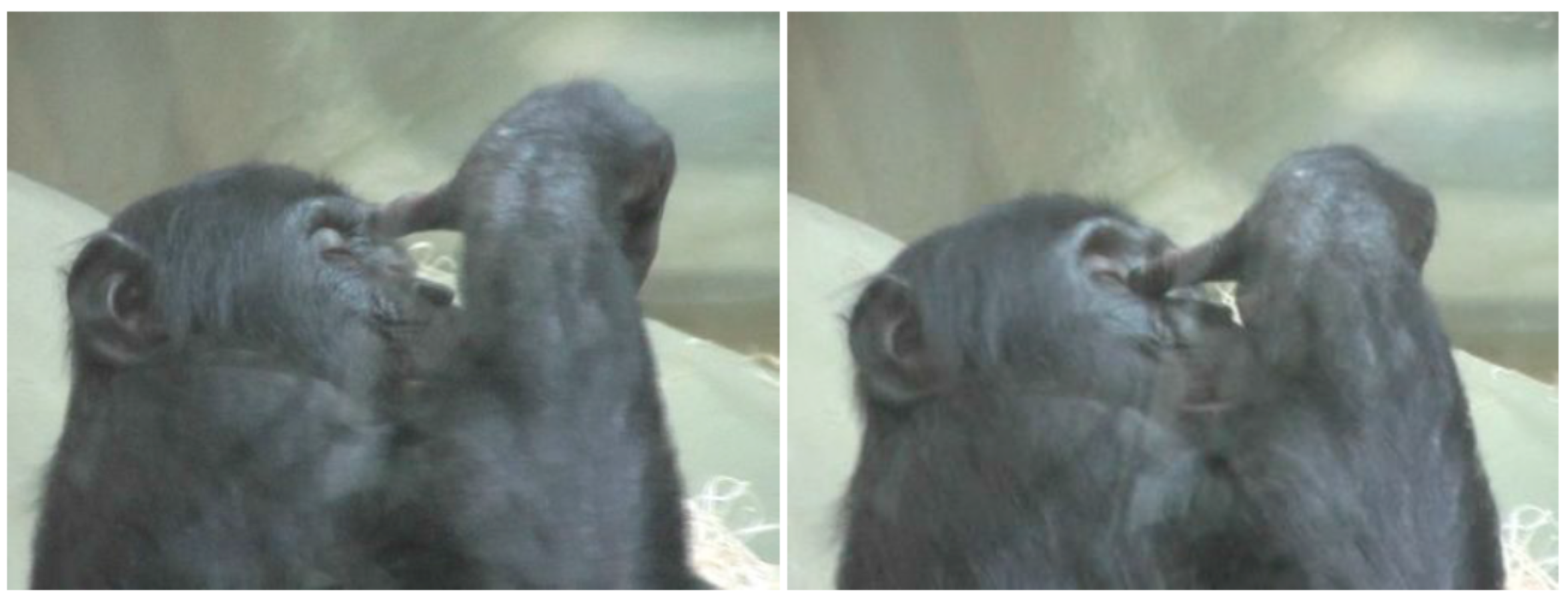

Supplement: Supplemental Information 6 — Left: neutral browridge. Right: AU1+2 - Brow Raiser. Still frames from video by PK. [file peerj-13-19484-s006.png]

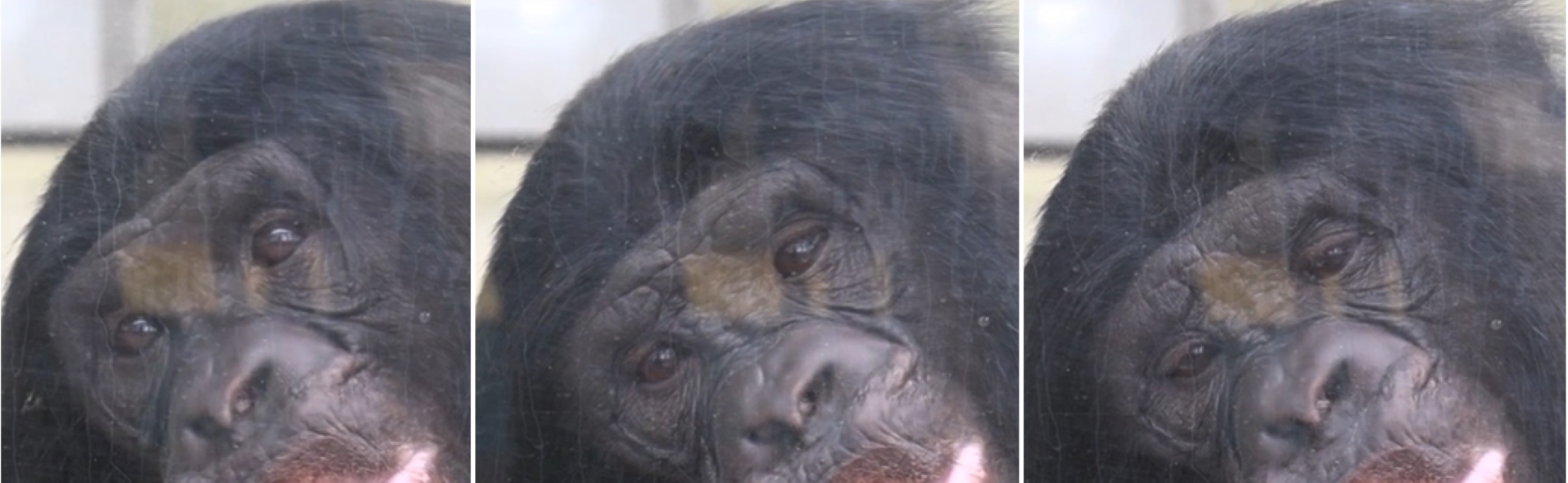

Supplement: Supplemental Information 7 — Left: AU1+2 - Brow Raiser. Centre: Neutral browridge. Left: AU41 - Glabella Lowerer. Still frames from video by ML. [file peerj-13-19484-s007.png]

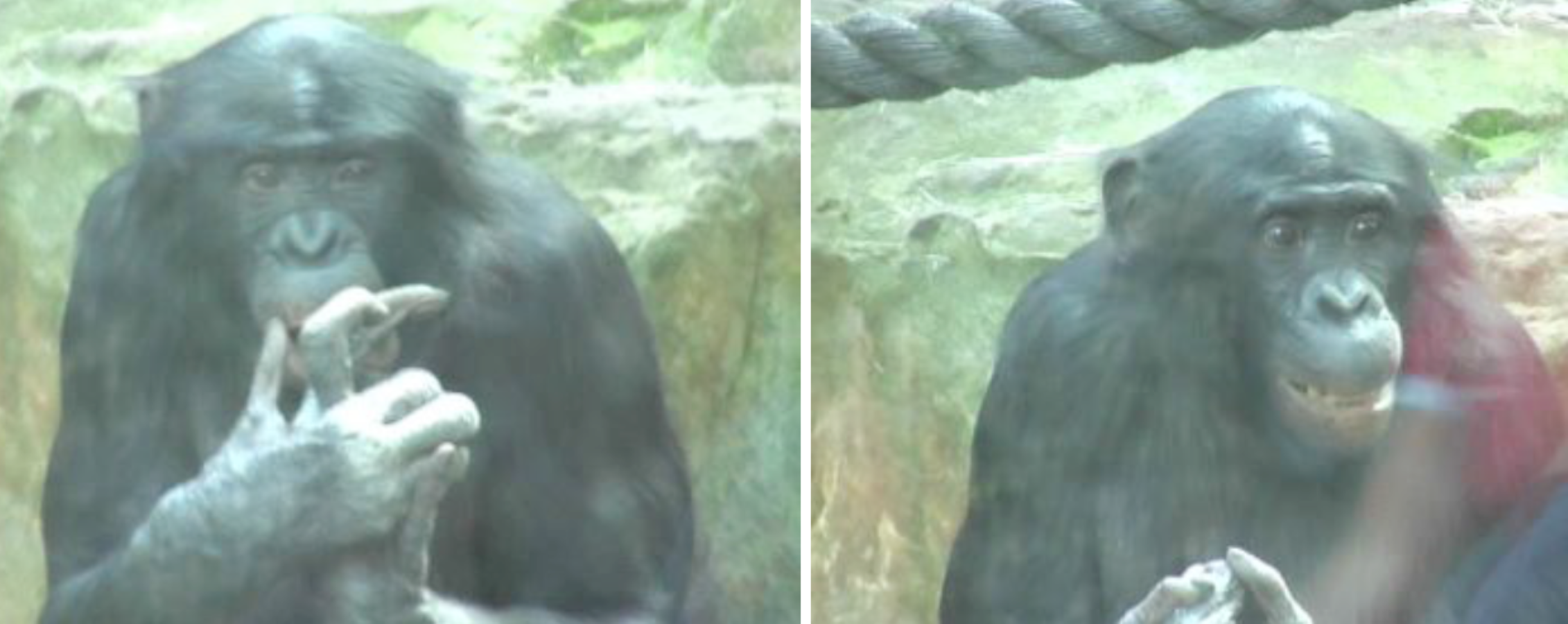

Supplement: Supplemental Information 8 — Left: neutral eye region. Right: AU5 - Upper Lid Raiser. Other AUs are present (including AU1+2). Still frames from video by PK. [file peerj-13-19484-s008.png]

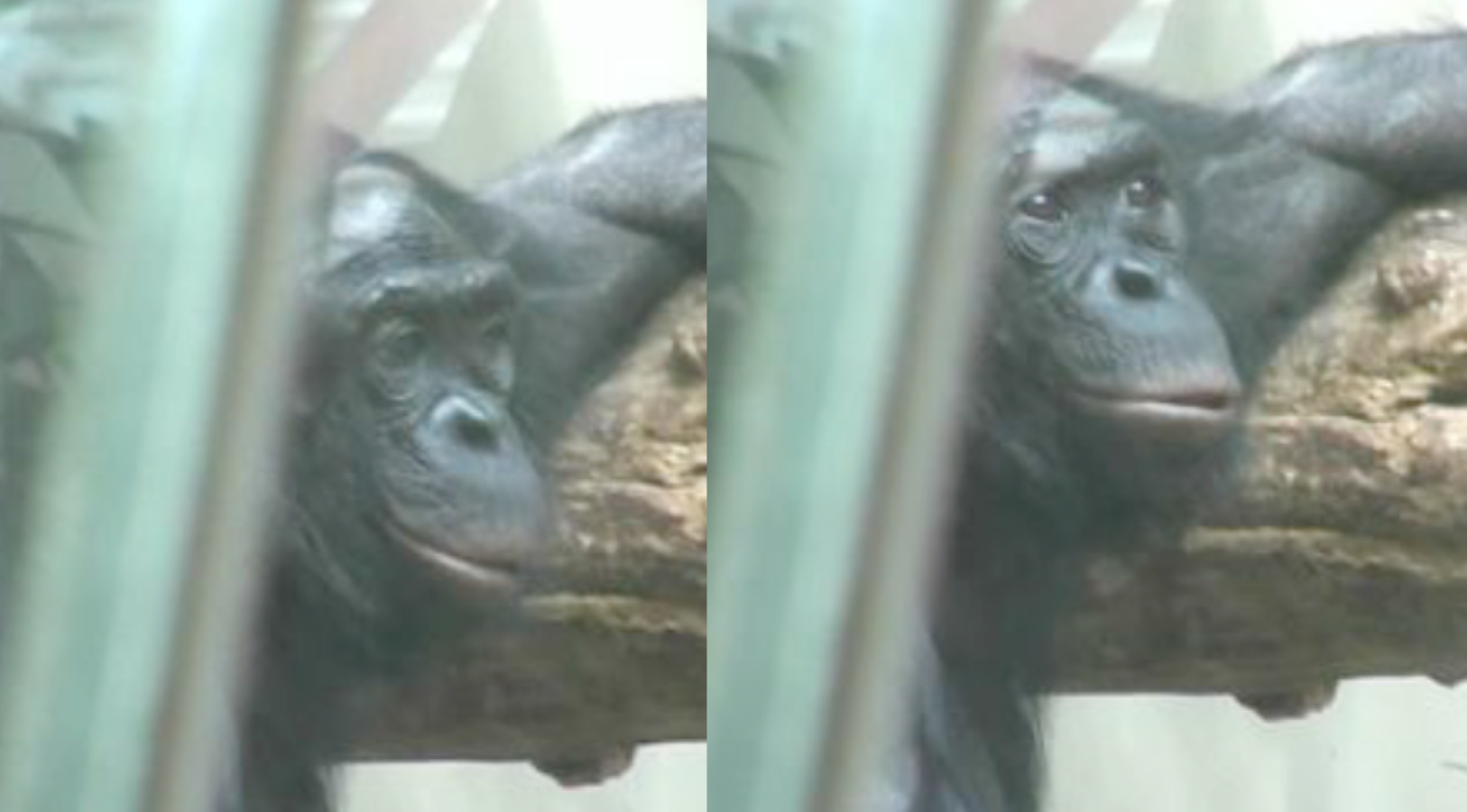

Supplement: Supplemental Information 9 — Left: neutral eye region. Right: AU5 - Upper Lid Raiser. Other AUs are present (including AD63 - Eyes Up and AD53 - Head Up). Still frames from video by PK. [file peerj-13-19484-s009.png]

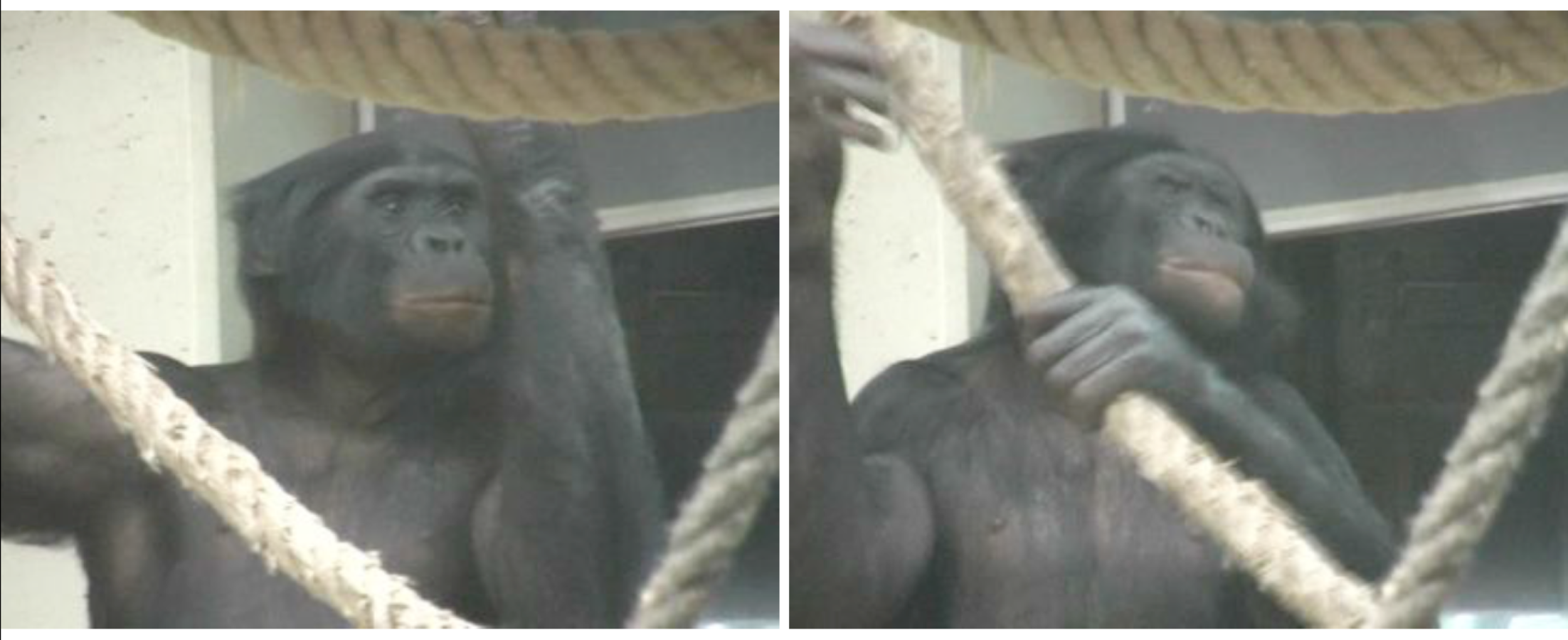

Supplement: Supplemental Information 10 — Left: neutral under eye area; Right: AU6 - Cheek Raiser. Other AUs present. Still frames from video by PK. [file peerj-13-19484-s010.png]

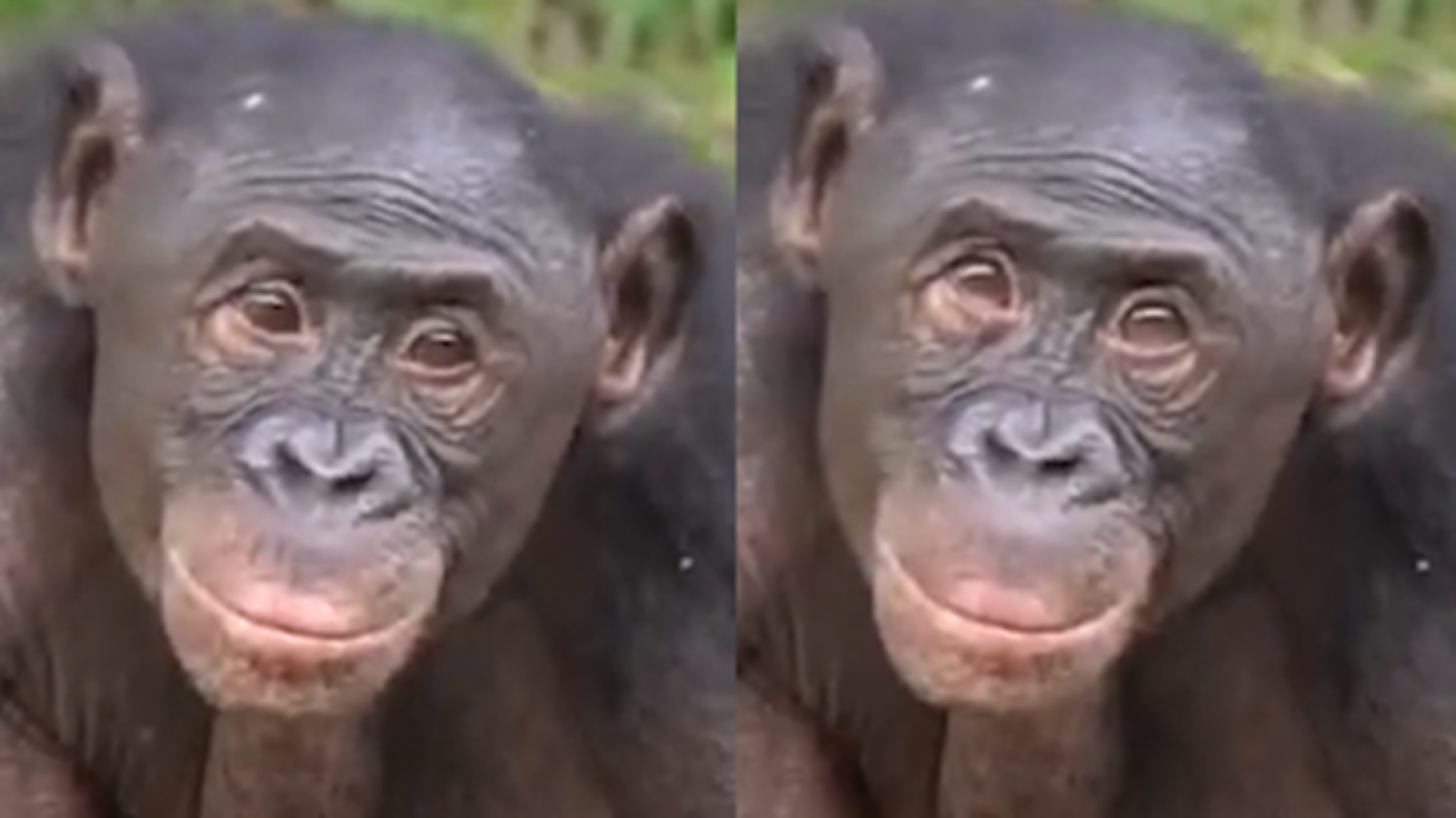

Supplement: Supplemental Information 11 — Left: neutral under eye area; Right: AU7 - Lid Tightener (more apparent on right eyelid than on the left, in which the lower eyelid is straighter and the eyeball is visibly more covered). Other AUs present. Still frames from S11 Video by Friends of Bonobos/Lola Ya Bonobo. [file peerj-13-19484-s011.png]

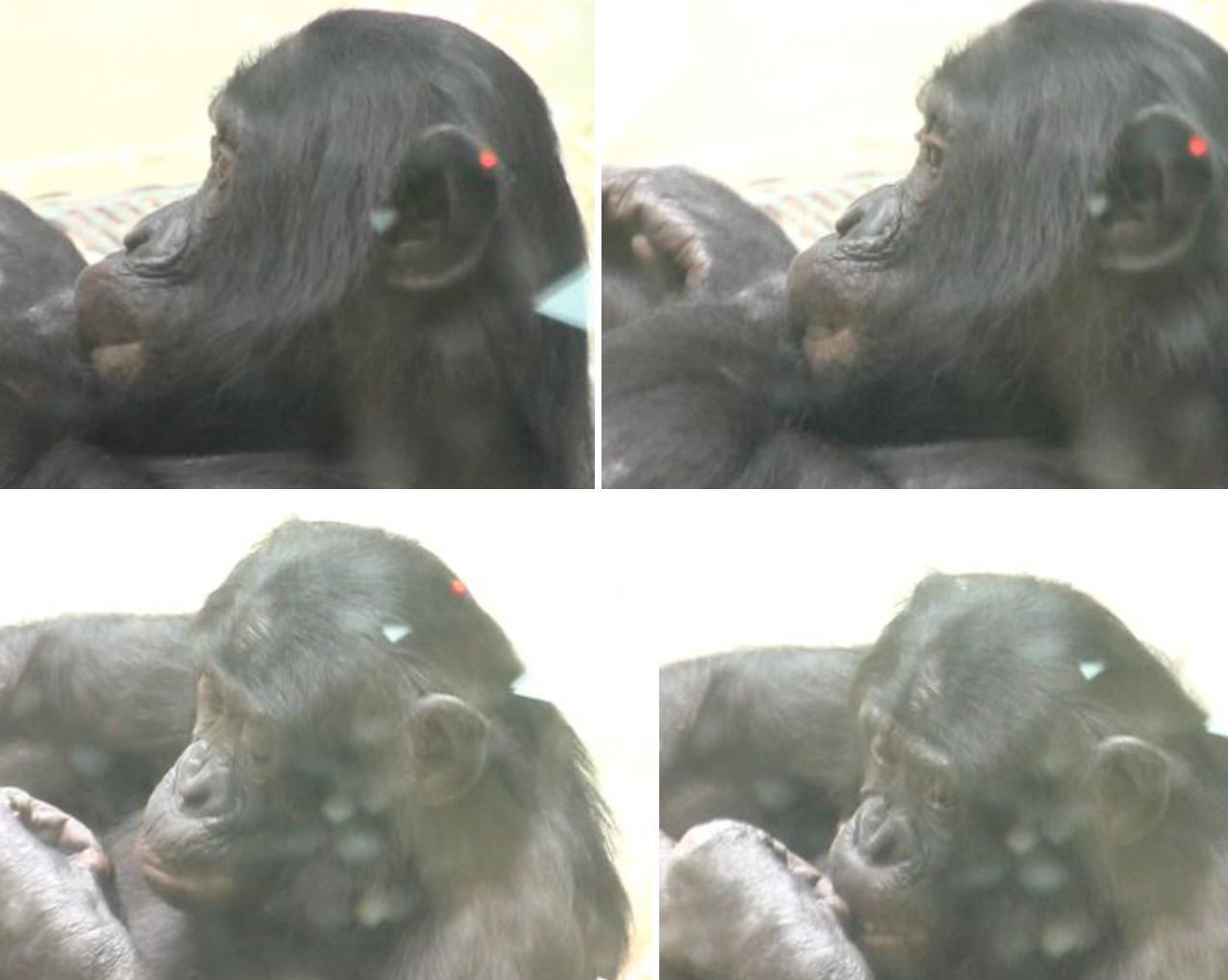

Supplement: Supplemental Information 12 — For both top and bottom frames: Left: neutral nose area; Right: AU9 - Nose Wrinkler. Other AUs present. Still frames from video by PK. [file peerj-13-19484-s012.png]

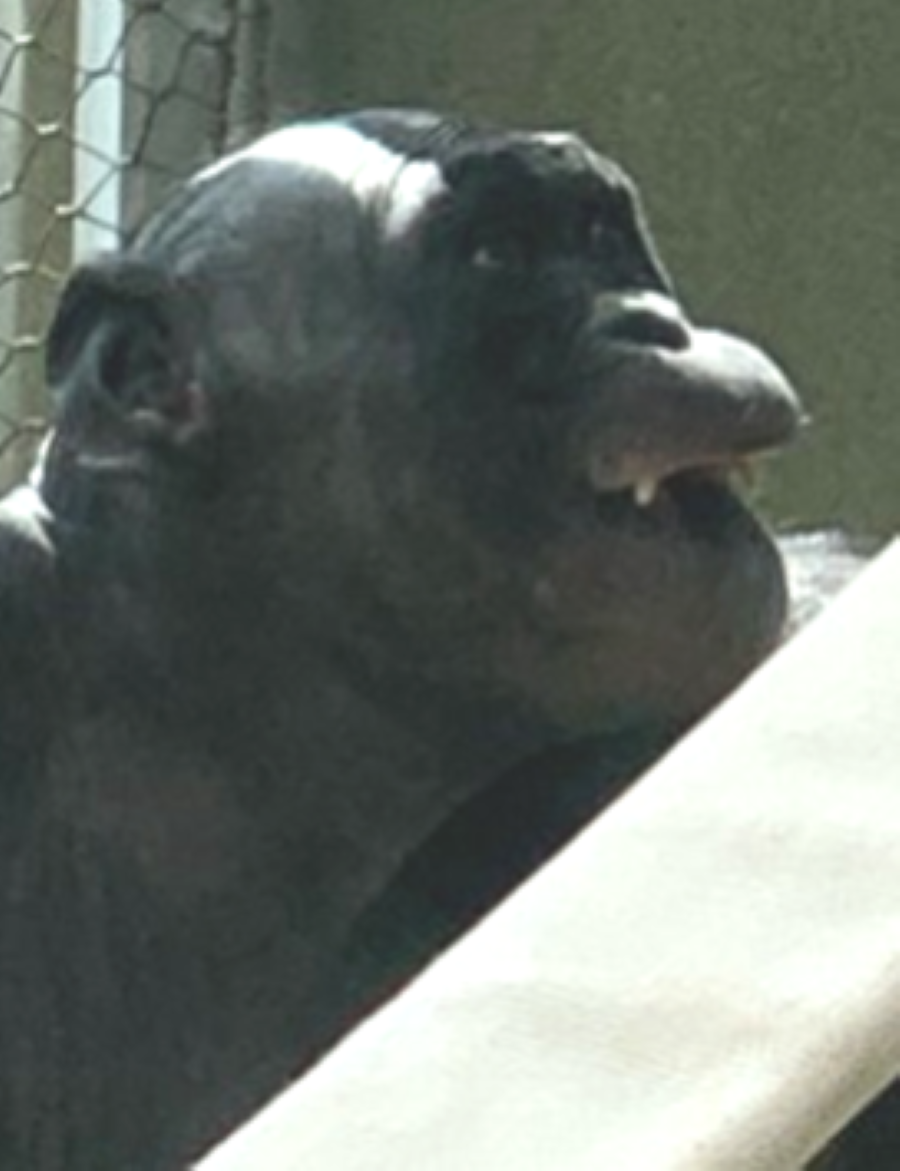

Supplement: Supplemental Information 13 — Other AUs present. Picture by ML. [file peerj-13-19484-s013.png]

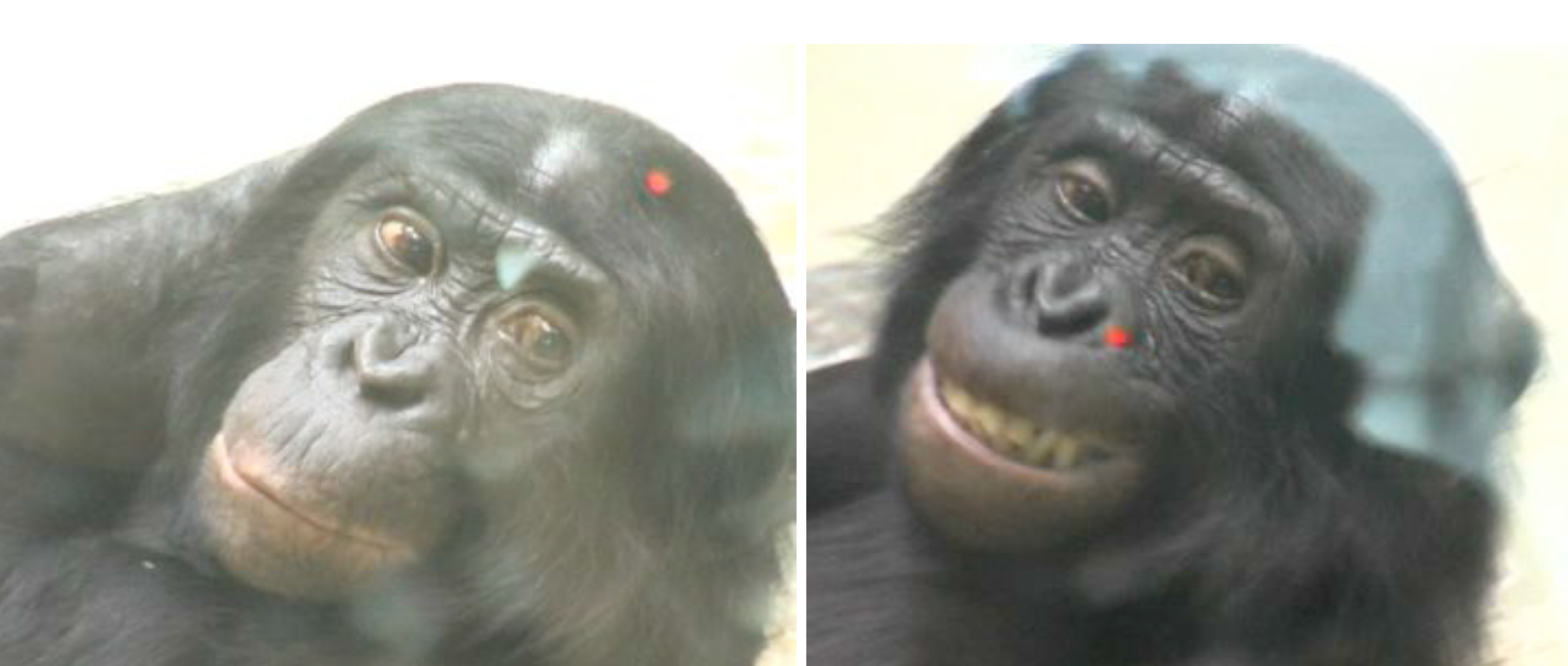

Supplement: Supplemental Information 14 — Other AUs present. Still frames from video by PK. [file peerj-13-19484-s014.png]

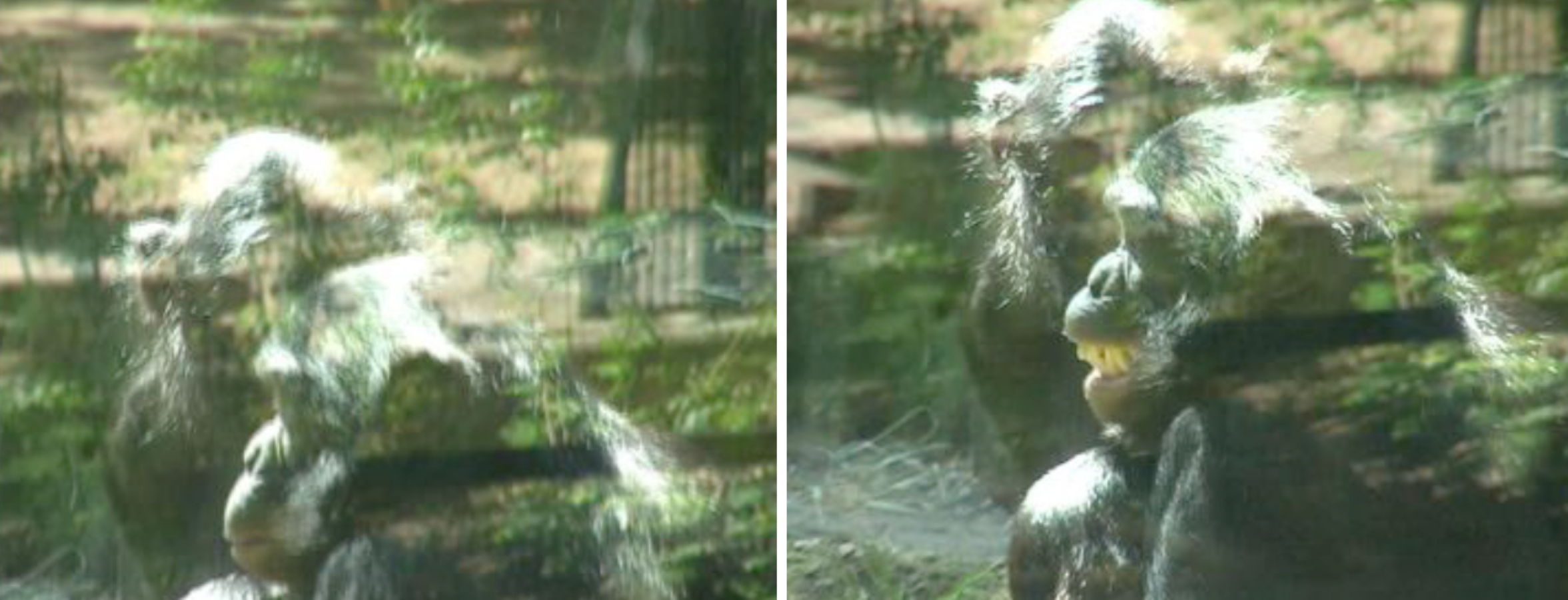

Supplement: Supplemental Information 15 — Other AUs present. Still frames from video by PK. [file peerj-13-19484-s015.png]

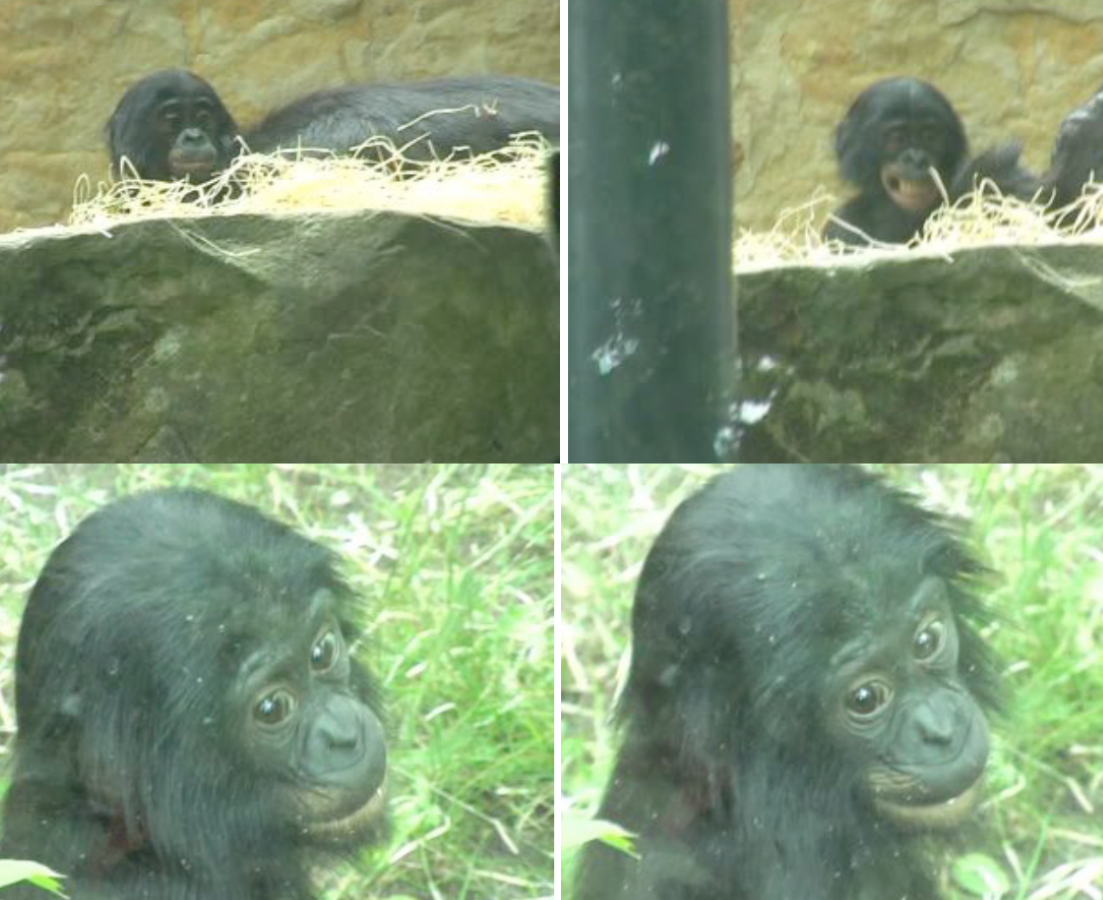

Supplement: Supplemental Information 16 — For both top and bottom frames: Left: neutral mouth corner area; Right: AU12 - Lip Corner Puller. Other AUs present. Still frames from video by PK. [file peerj-13-19484-s016.png]

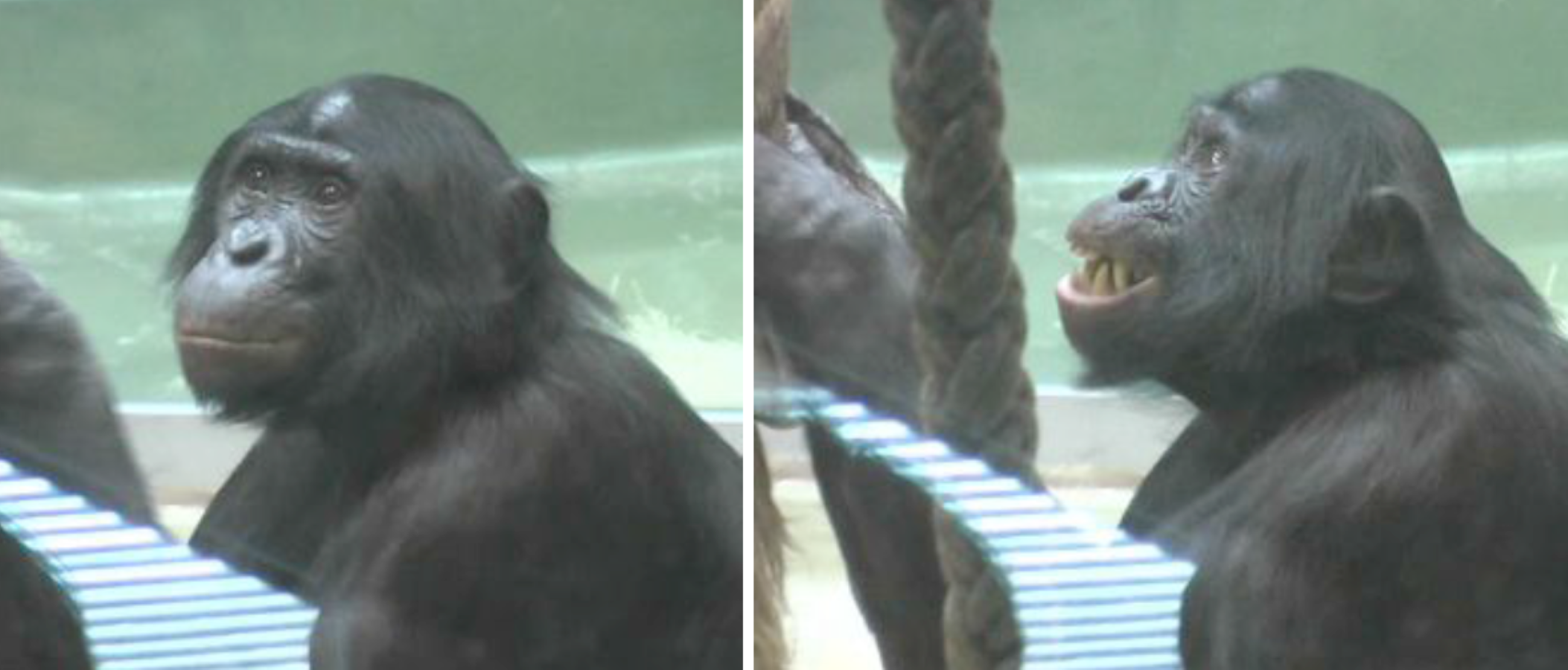

Supplement: Supplemental Information 17 — Left: neutral mouth corner area; Right: AU12 - Lip Corner Puller. Other AUs present. Still frames from video by PK. [file peerj-13-19484-s017.png]

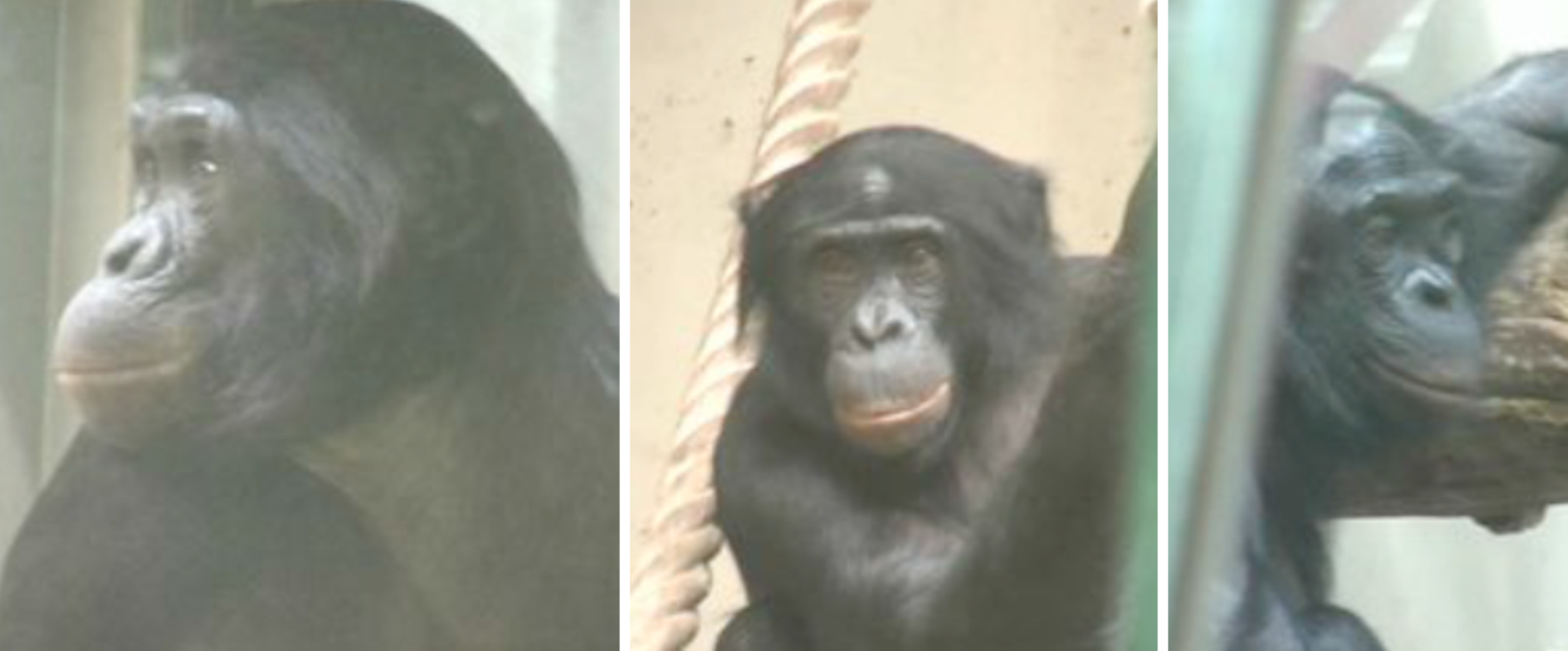

Supplement: Supplemental Information 18 — Slight upward curving in the neutral faces (AU0) of several individuals. Still frames from video by PK. [file peerj-13-19484-s018.png]

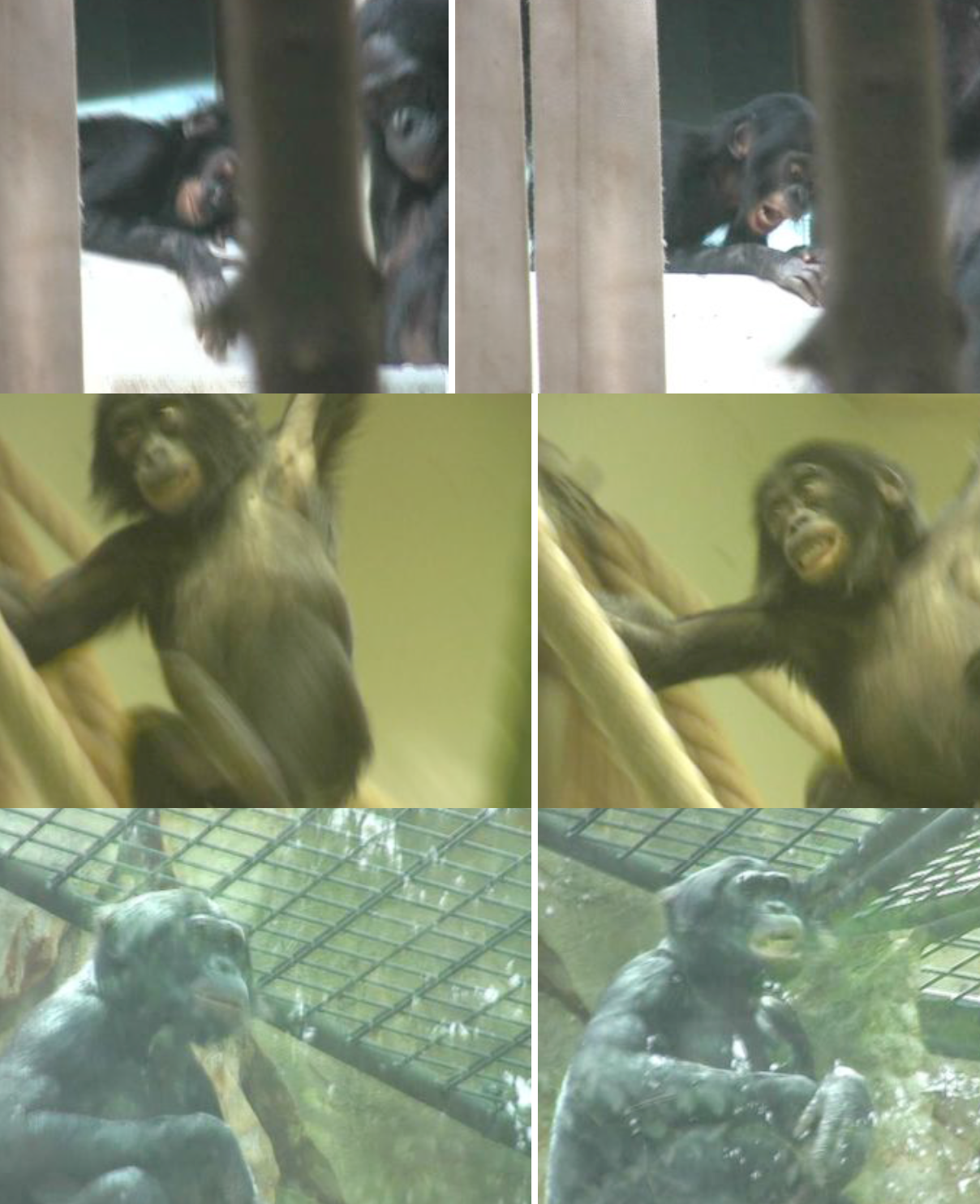

Supplement: Supplemental Information 19 — Other AUs present. Still frames from video by PK. [file peerj-13-19484-s019.png]

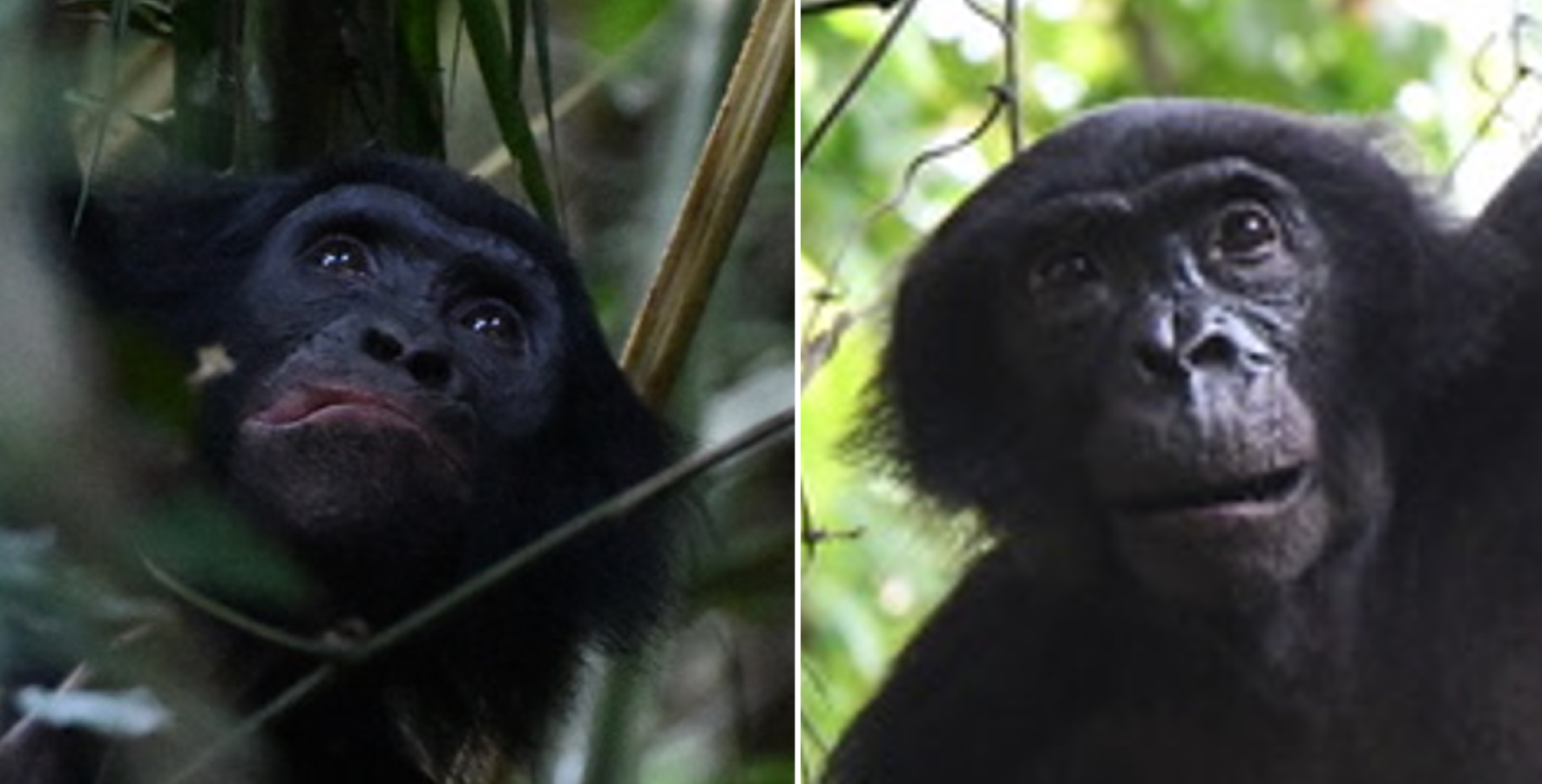

Supplement: Supplemental Information 20 — Right: AU16R - Right Lower Lip Depressor. Left: AU16L - Left Lower Lip Depressor. Other AUs present. Pictures by FW/Kokolopori Bonobo Research Project. [file peerj-13-19484-s020.png]

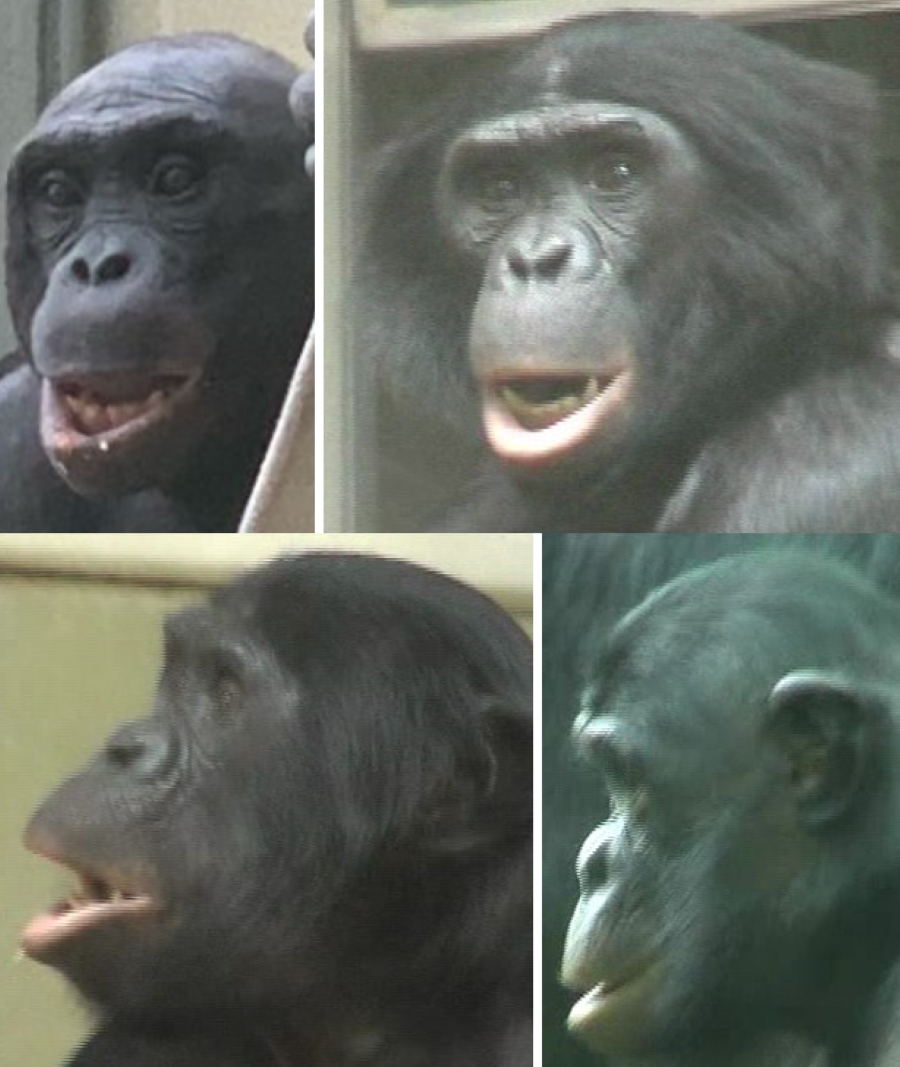

Supplement: Supplemental Information 21 — Other AUs present. Top left: picture by ML, top right, and bottom left and right: pictures by PK. [file peerj-13-19484-s021.png]

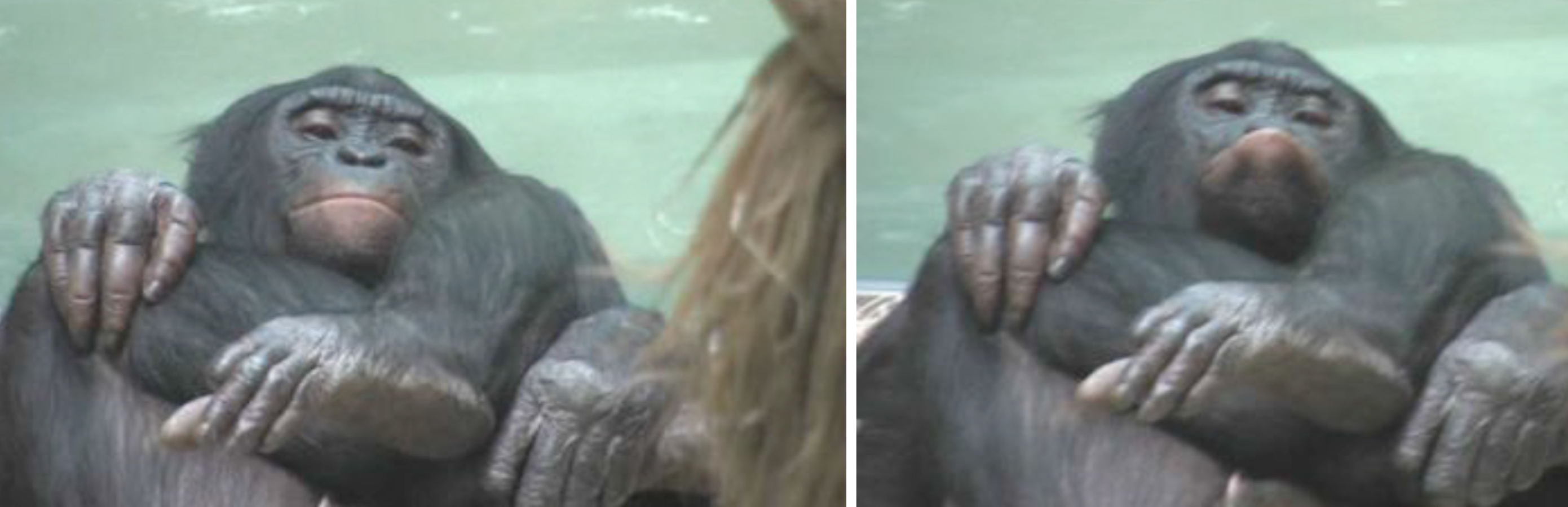

Supplement: Supplemental Information 22 — Left: neutral mental region; Right: AU17 - Chin Raiser. Other AUs present. Still frames from videos by PK. [file peerj-13-19484-s022.png]

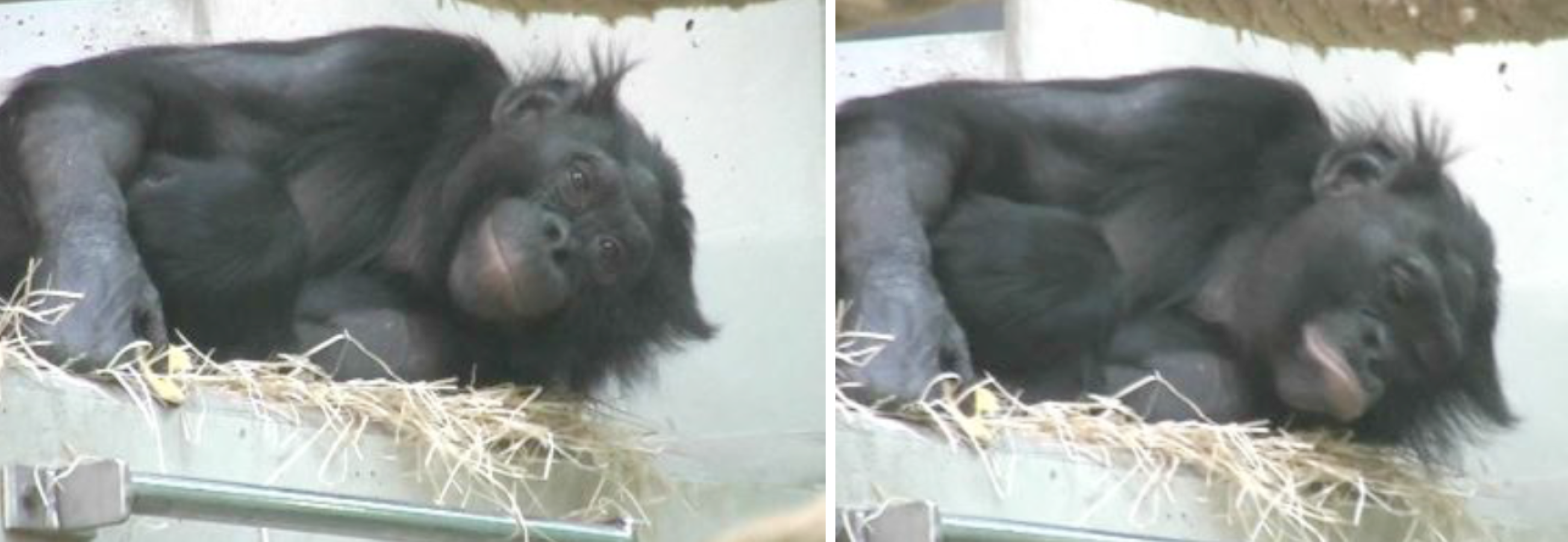

Supplement: Supplemental Information 23 — Left: neutral mental region; Right: AU17 - Chin Raiser. Other AUs present. Still frames from videos by PK. [file peerj-13-19484-s023.png]

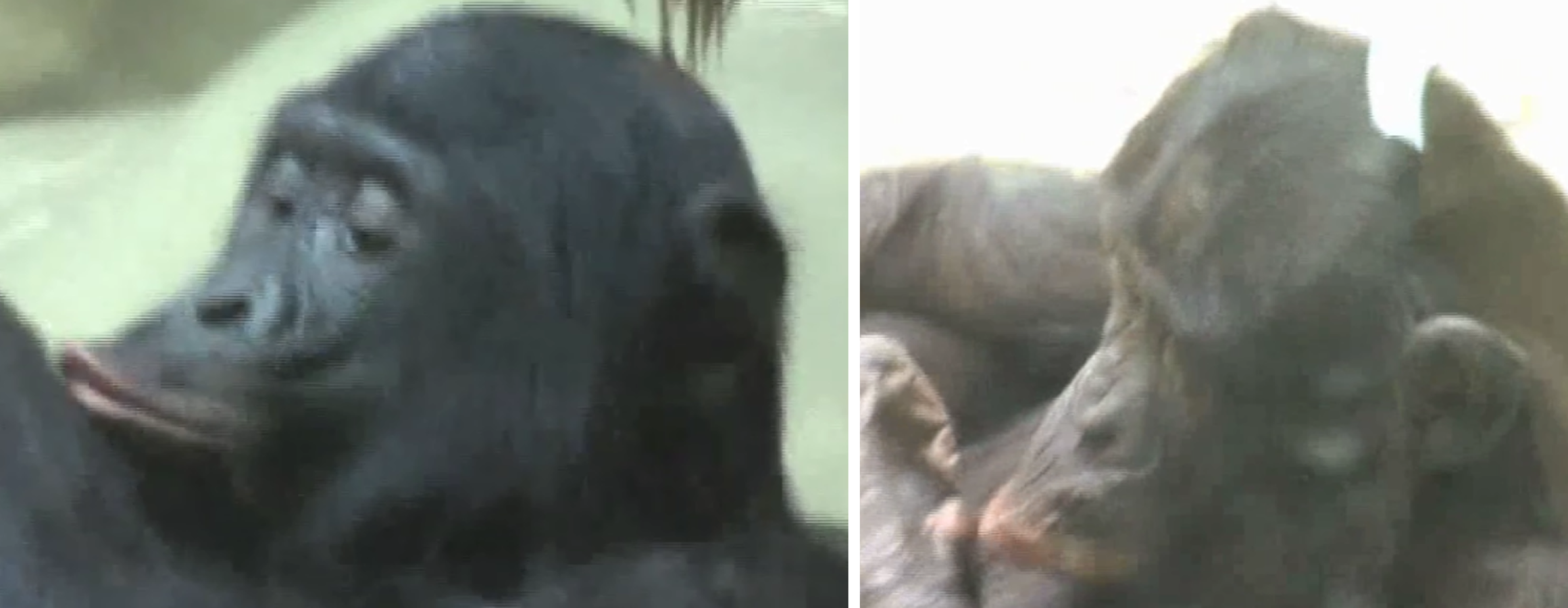

Supplement: Supplemental Information 24 — Left: note the slight outward flaring of the lower lip; Right: note the slight outward flaring of the top lip. Due to the projection of the lips and the slight flaring outwards of at least one lip, in the present work, both examples are here classified as AU22 - Lip Funneler (see next section for AU22 description). Still frames from video by PK. [file peerj-13-19484-s024.png]

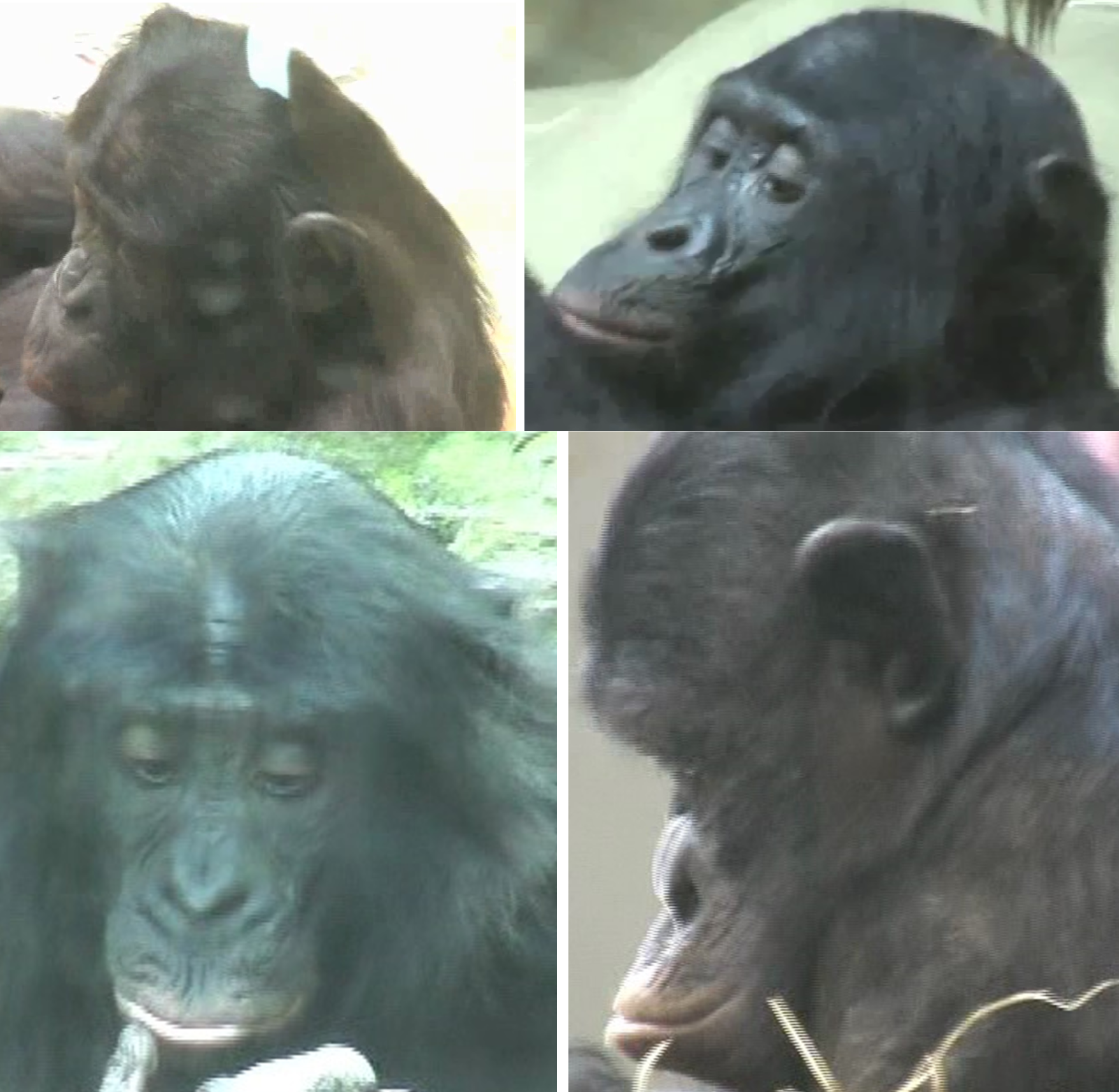

Supplement: Supplemental Information 25 — The lip corners are pushed medially and wrinkling of the upper lip is observed. No outwards flare of the lips is visible. Other AUs present. Top: Still frames from video by PK with AU18. Bottom: Pictures by PK. [file peerj-13-19484-s025.png]

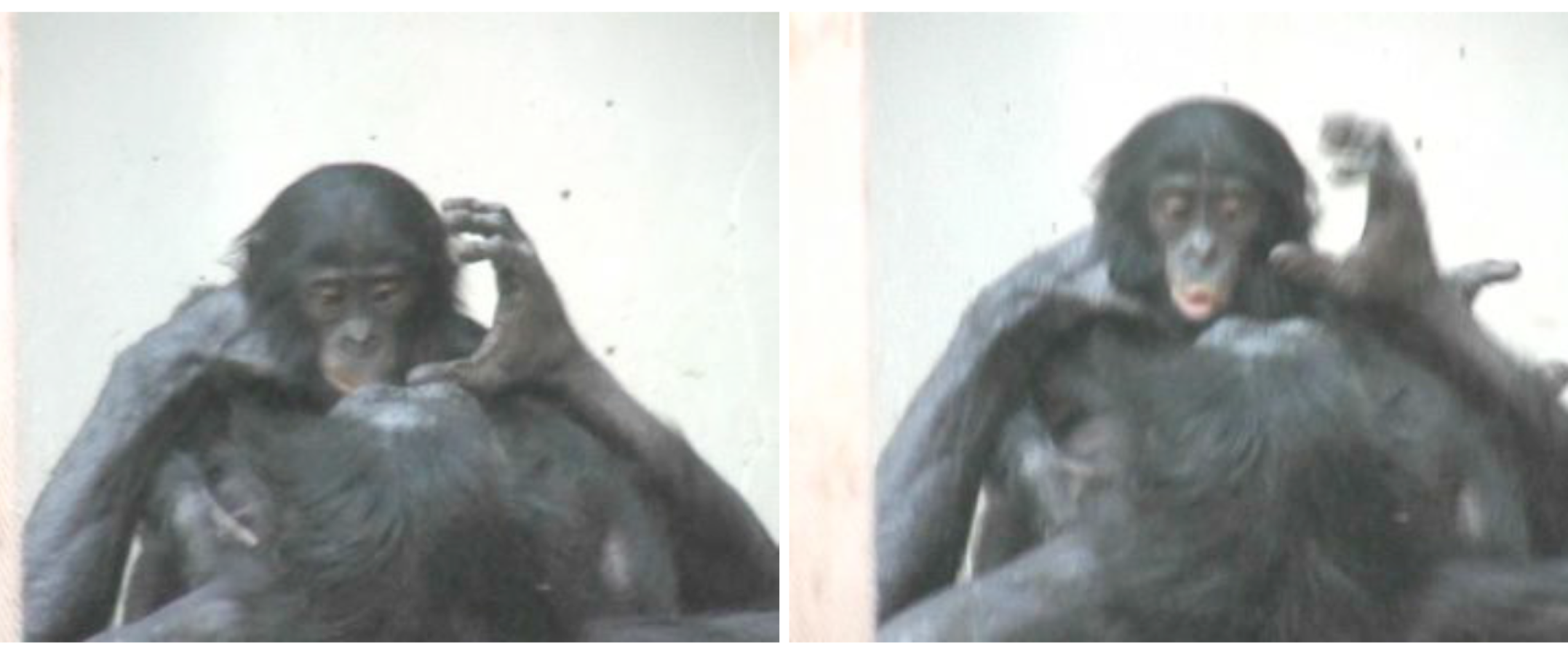

Supplement: Supplemental Information 26 — Still frames from video by PK. [file peerj-13-19484-s026.png]

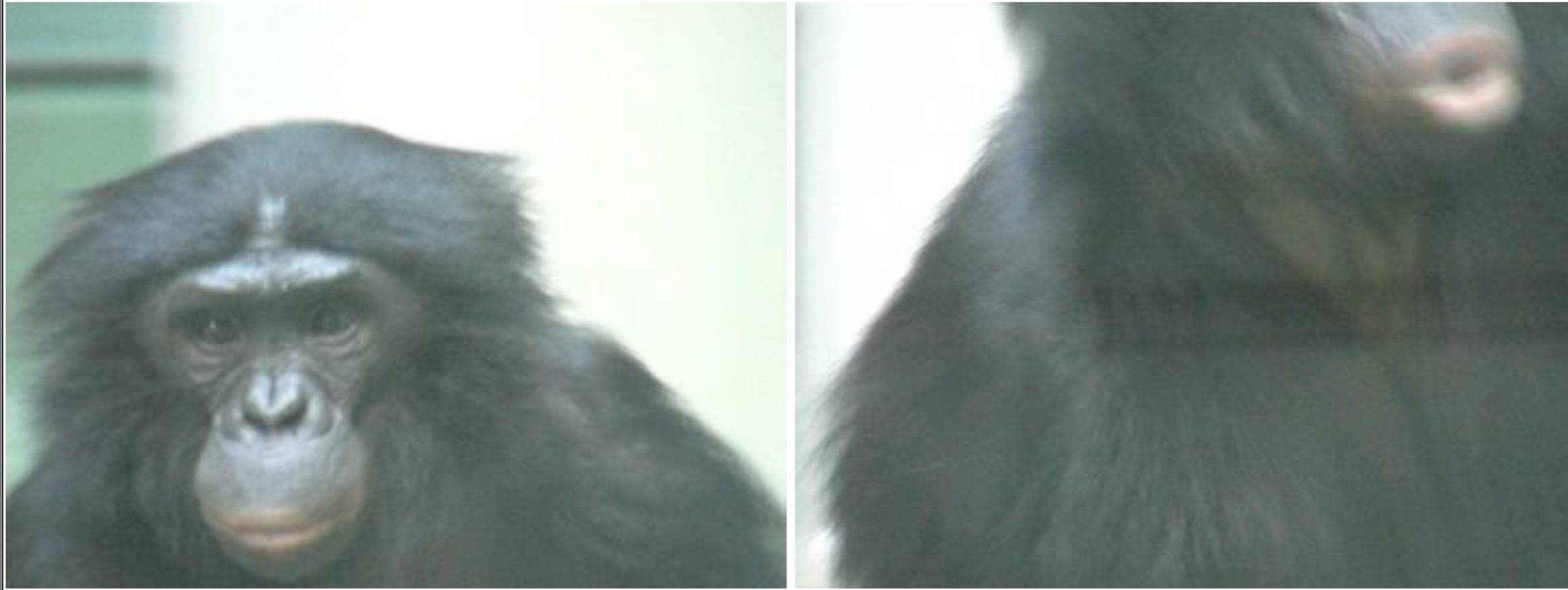

Supplement: Supplemental Information 27 — Still frames from video by PK. [file peerj-13-19484-s027.png]

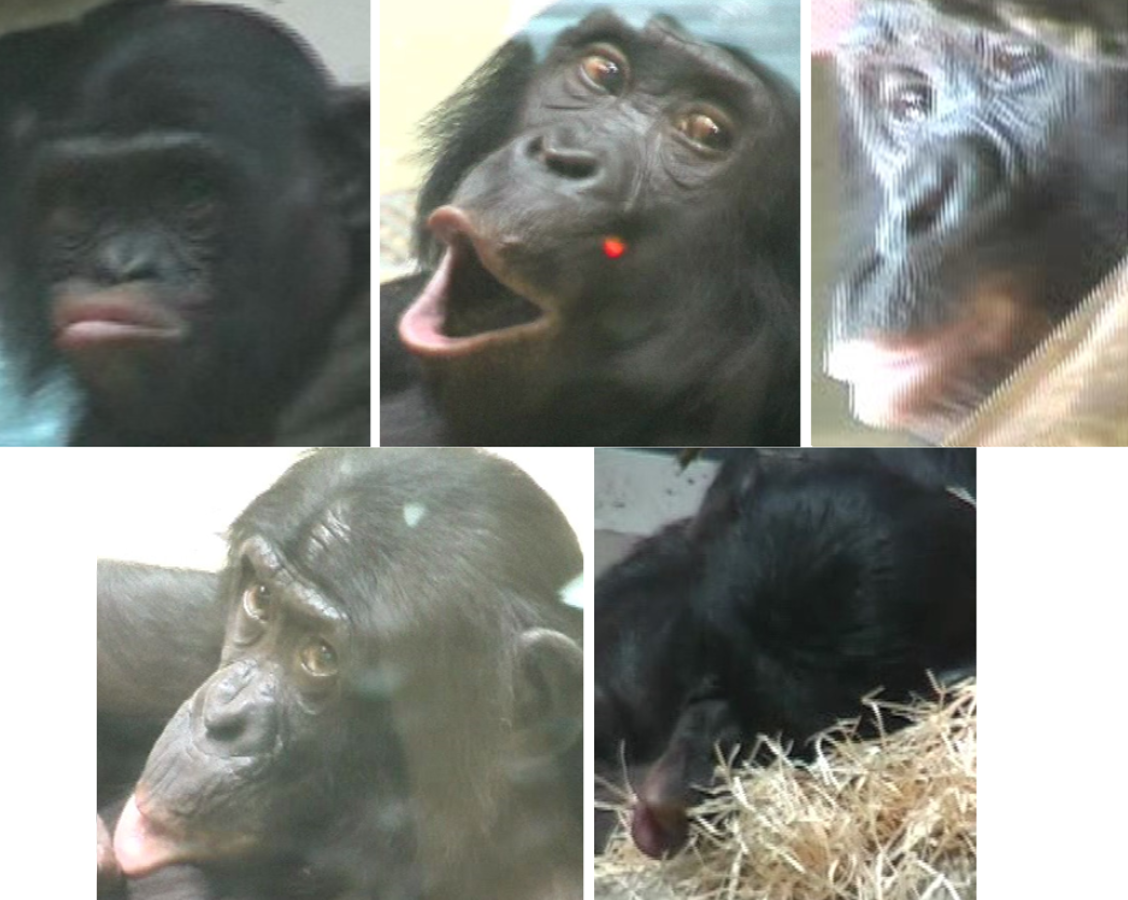

Supplement: Supplemental Information 28 — Top Left: AU22 + AU17, with AU17 action pushing the lower lip together with the upper lip. Top Centre: AU22 + AU27. Top Right: AU22 without AU25 (AU17 is likely present closing the lips together). Bottom: AU22 + AU25. Note the flare of at least one of the lips in all examples, presenting a typical flattened shape. Still frames and pictures by PK. [file peerj-13-19484-s028.png]

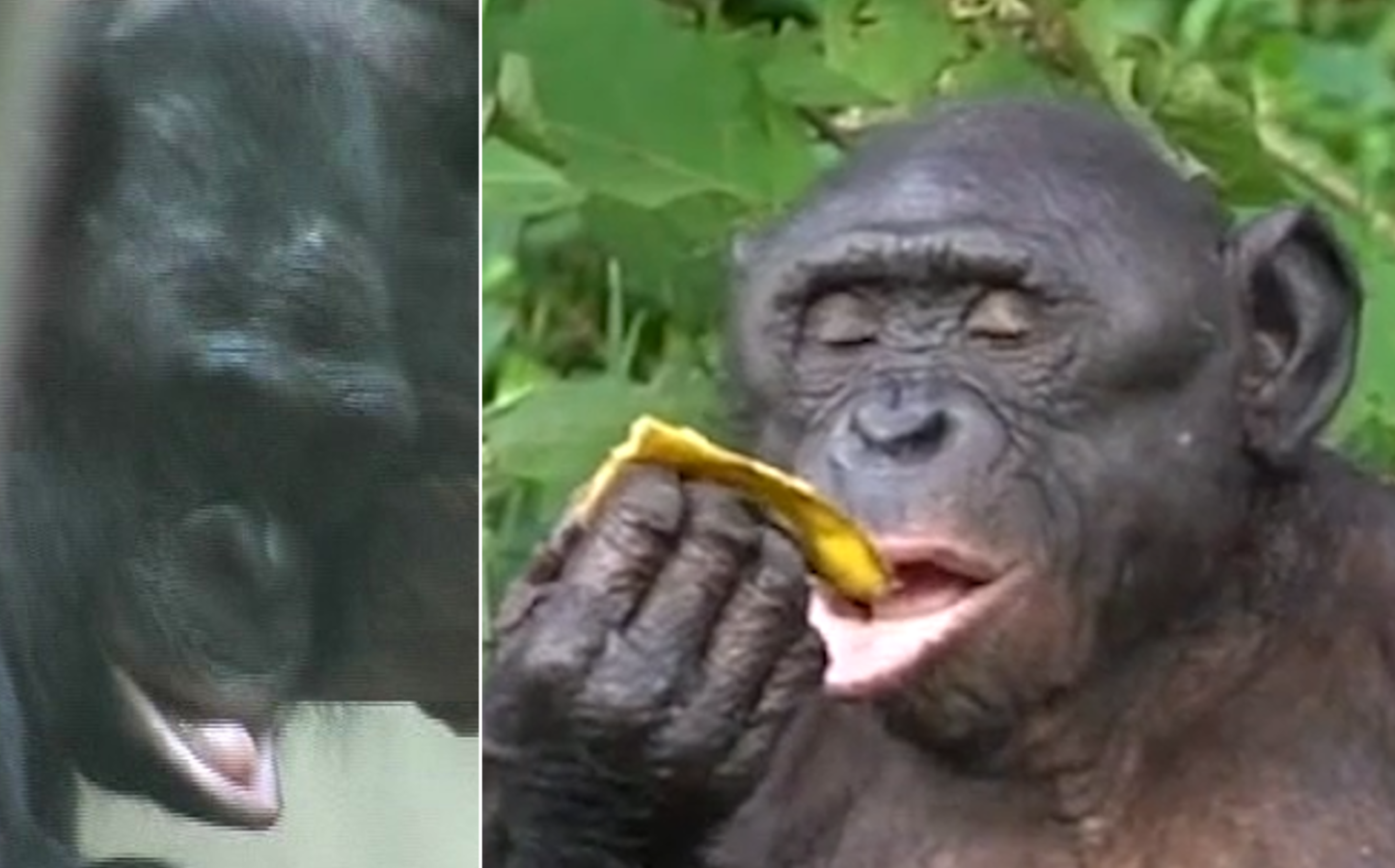

Supplement: Supplemental Information 29 — Other AUs present. Left: still frame from video by PK, Right: still frame from video by Friends of Bonobos/Lola Ya Bonobo. [file peerj-13-19484-s029.png]

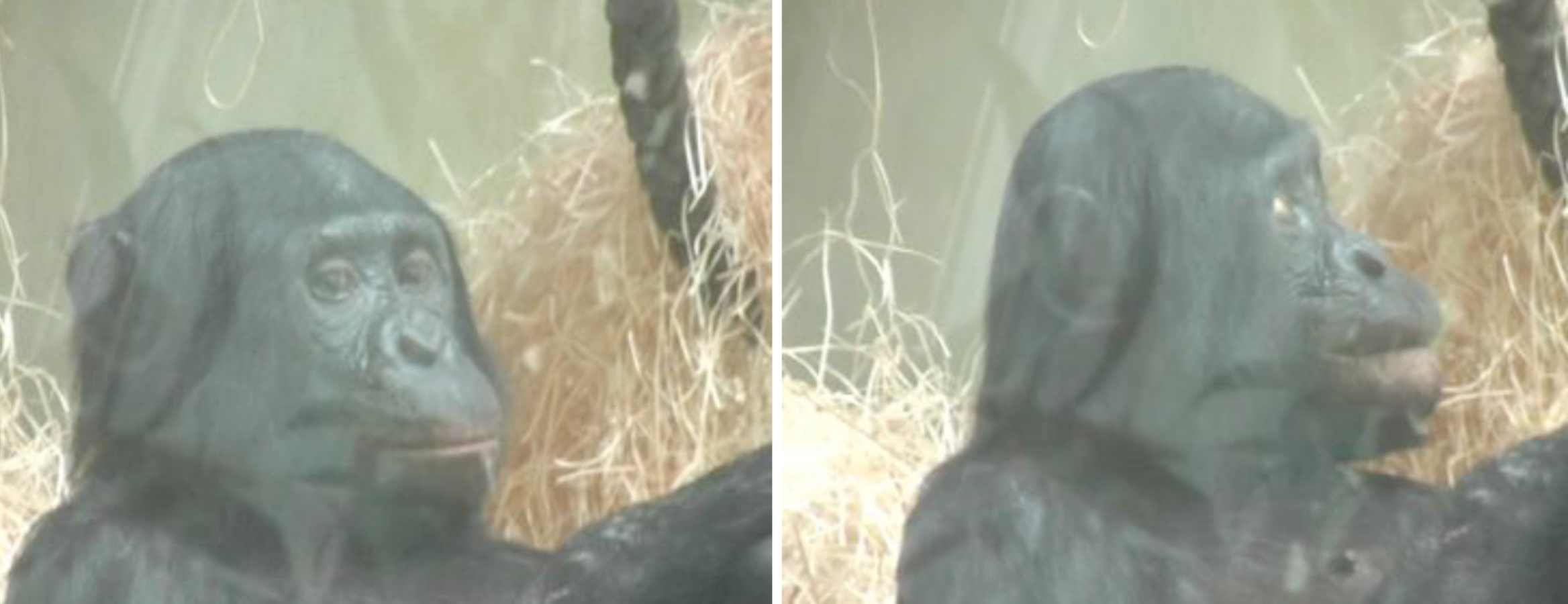

Supplement: Supplemental Information 30 — Pictures by PK. [file peerj-13-19484-s030.png]

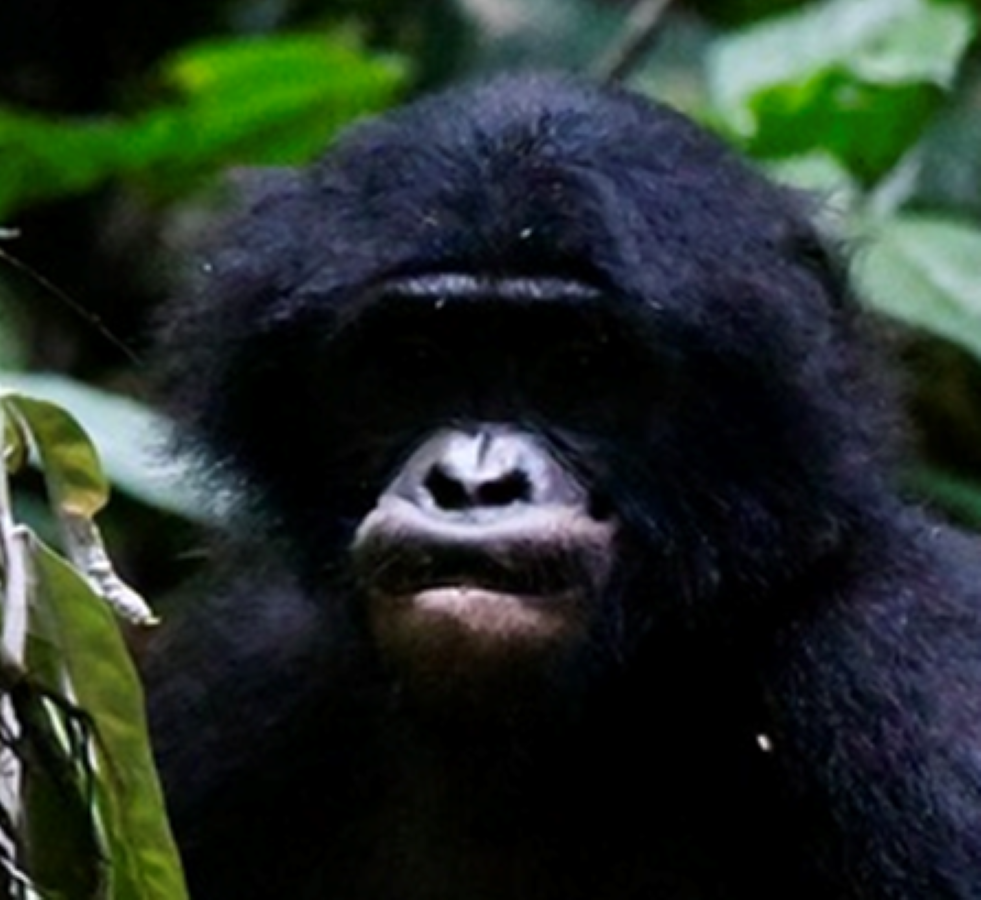

Supplement: Supplemental Information 31 — Picture by FW/Kokolopori Bonobo Research Project. [file peerj-13-19484-s031.png]

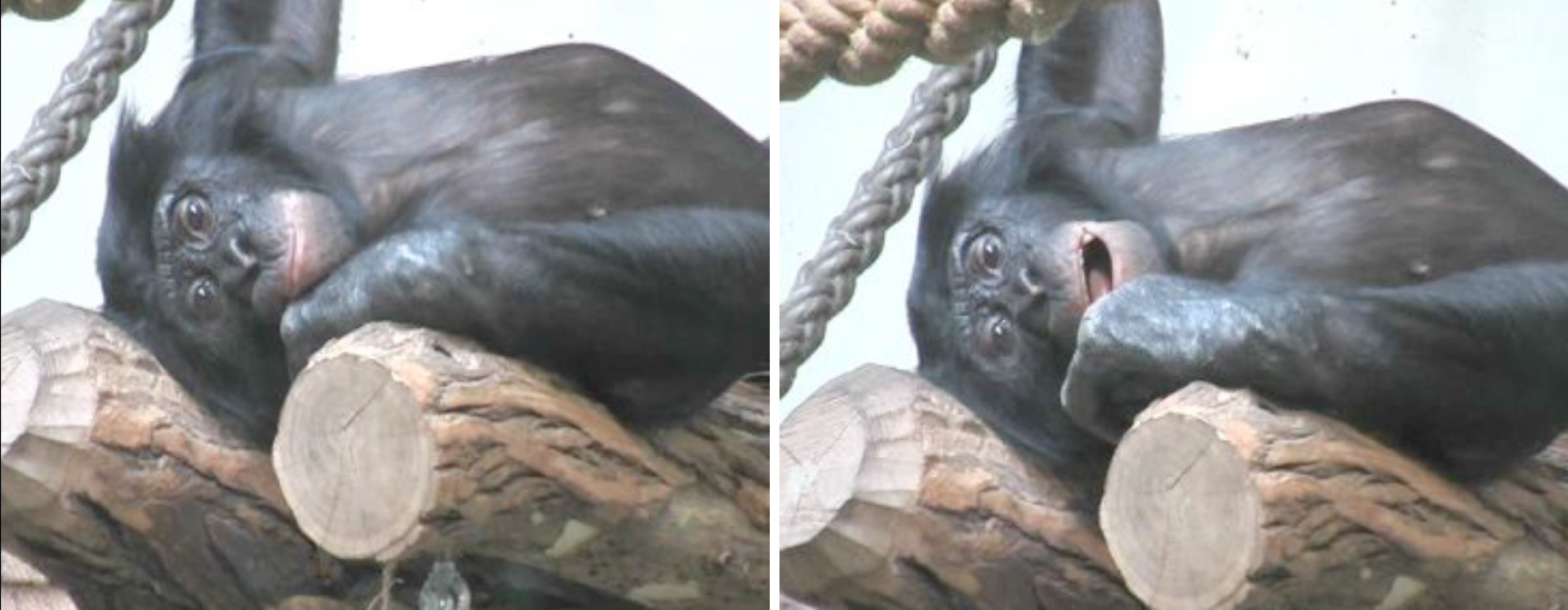

Supplement: Supplemental Information 32 — Pictures by PK. [file peerj-13-19484-s032.png]

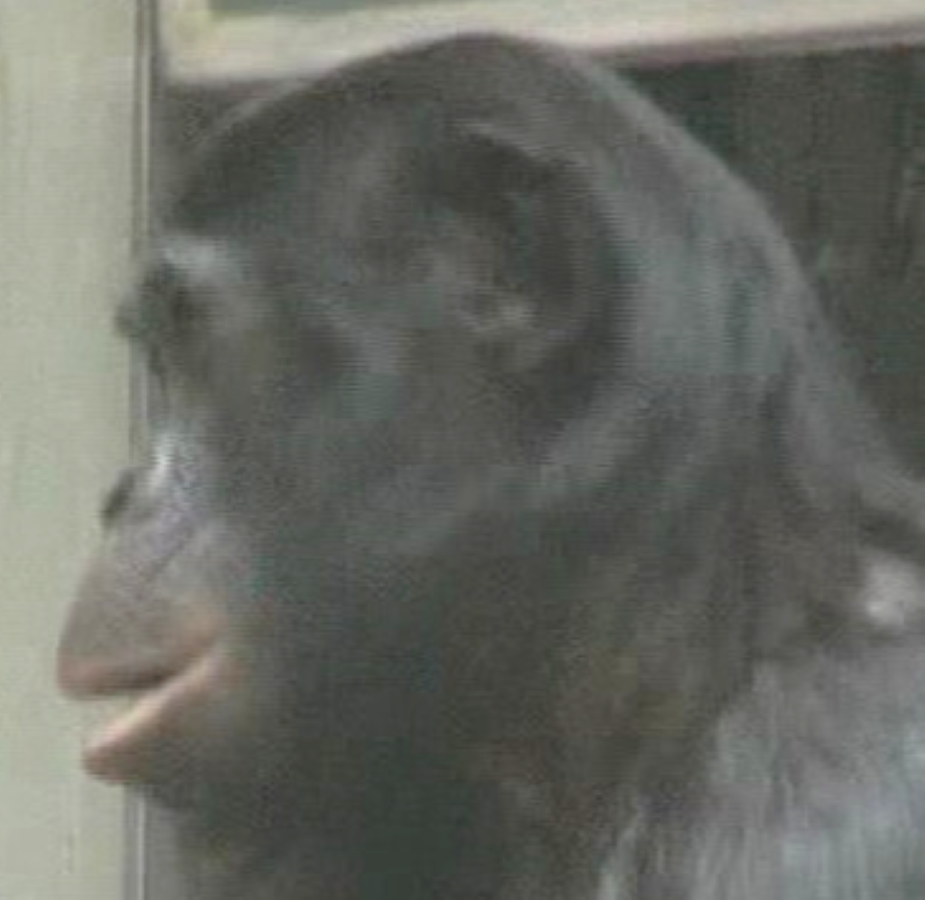

Supplement: Supplemental Information 33 — Picture by PK. [file peerj-13-19484-s033.png]

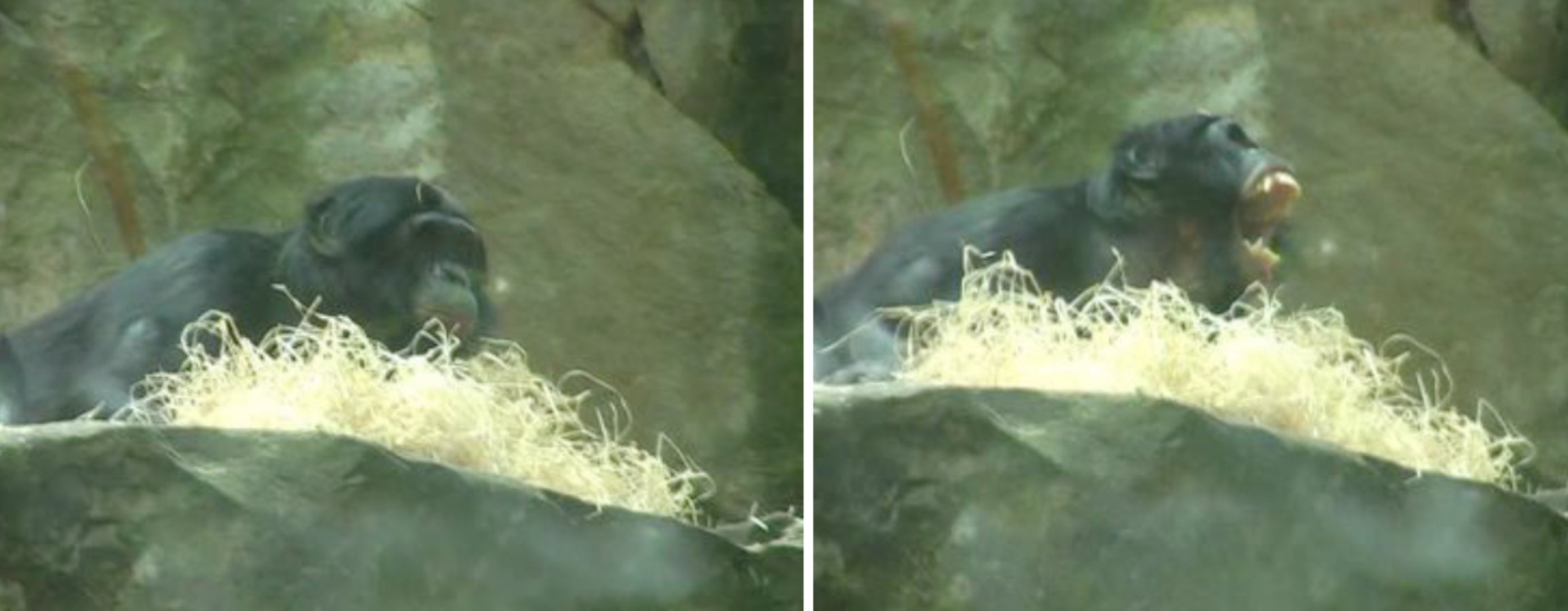

Supplement: Supplemental Information 34 — Other AUs present. Still frames from video by PK. [file peerj-13-19484-s034.png]

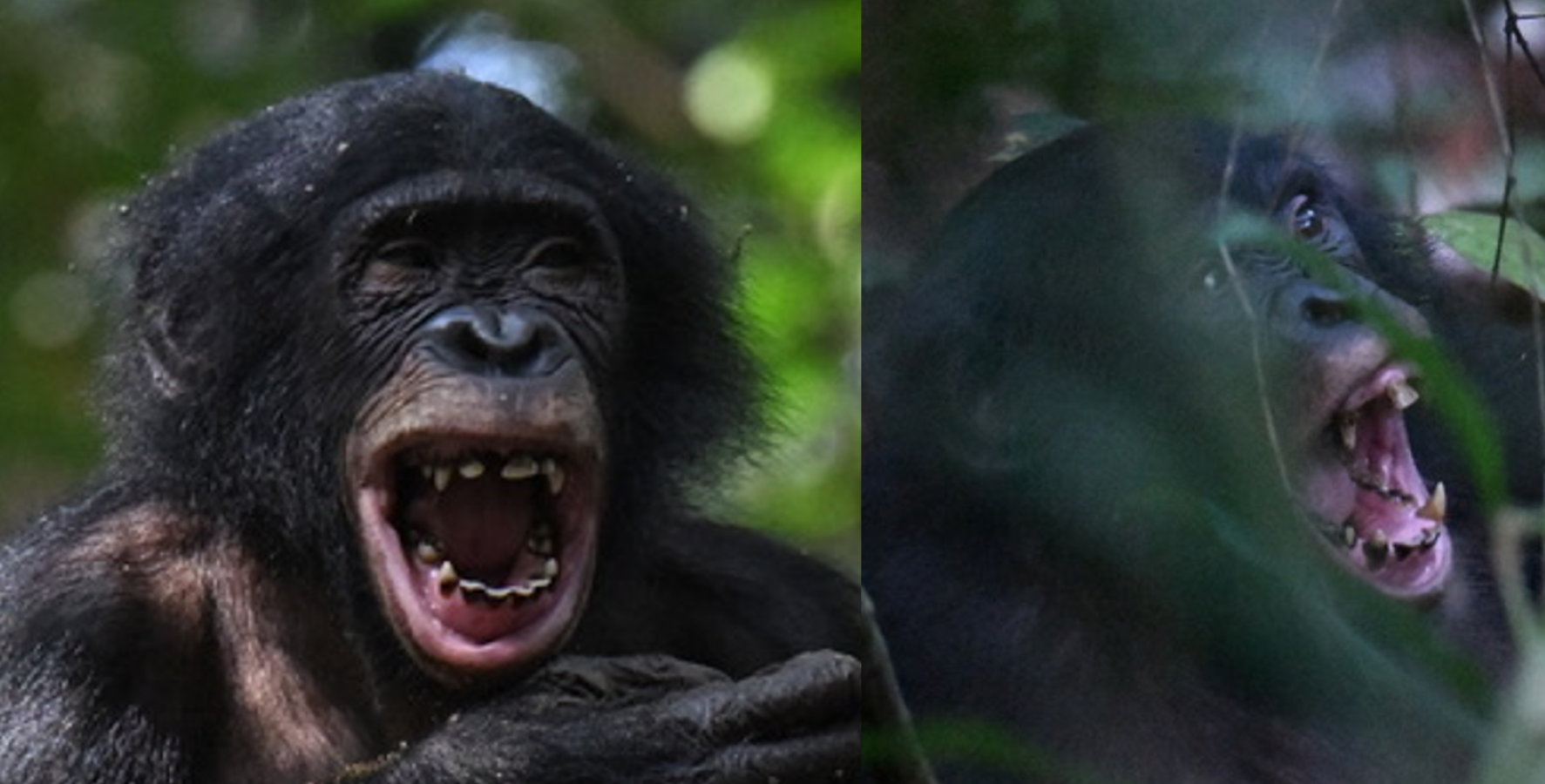

Supplement: Supplemental Information 35 — Other AUs present. Pictures by FW/Kokolopori Bonobo Research Project. [file peerj-13-19484-s035.png]

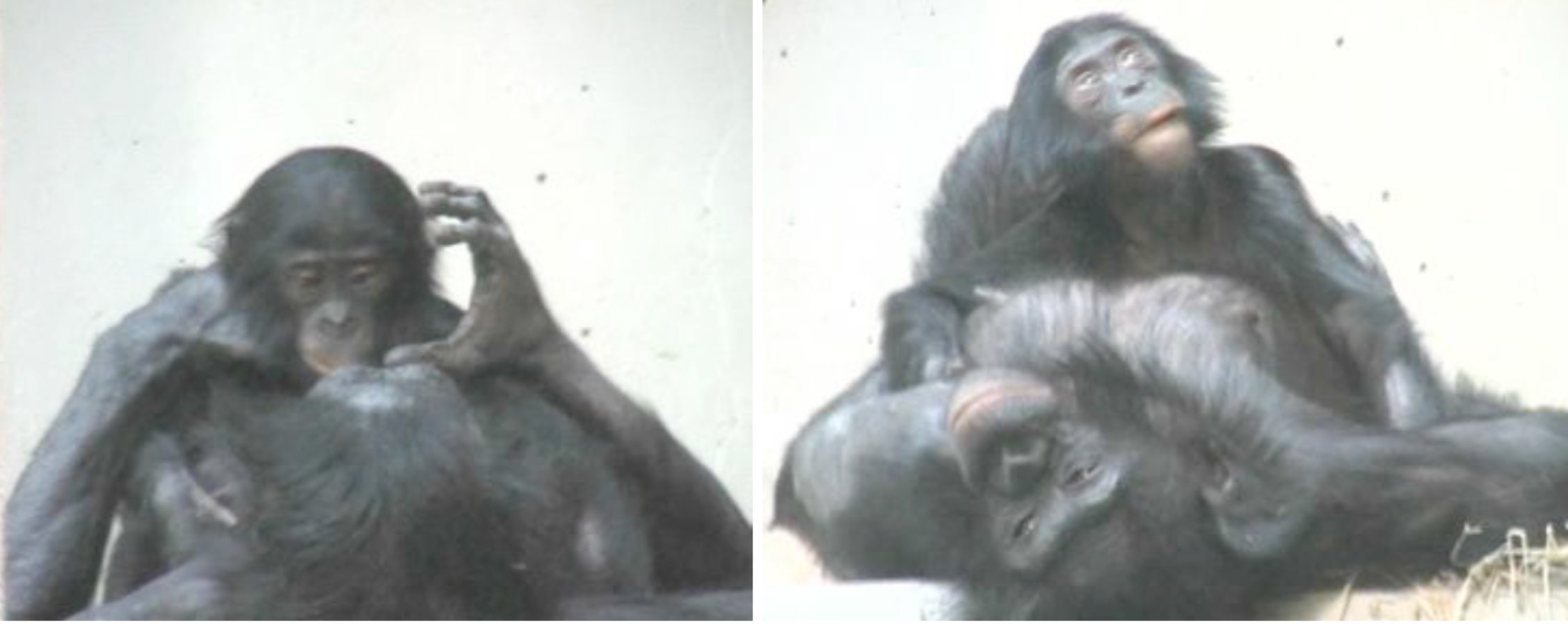

Supplement: Supplemental Information 36 — Other AUs present. Still frames from video by PK. [file peerj-13-19484-s036.png]

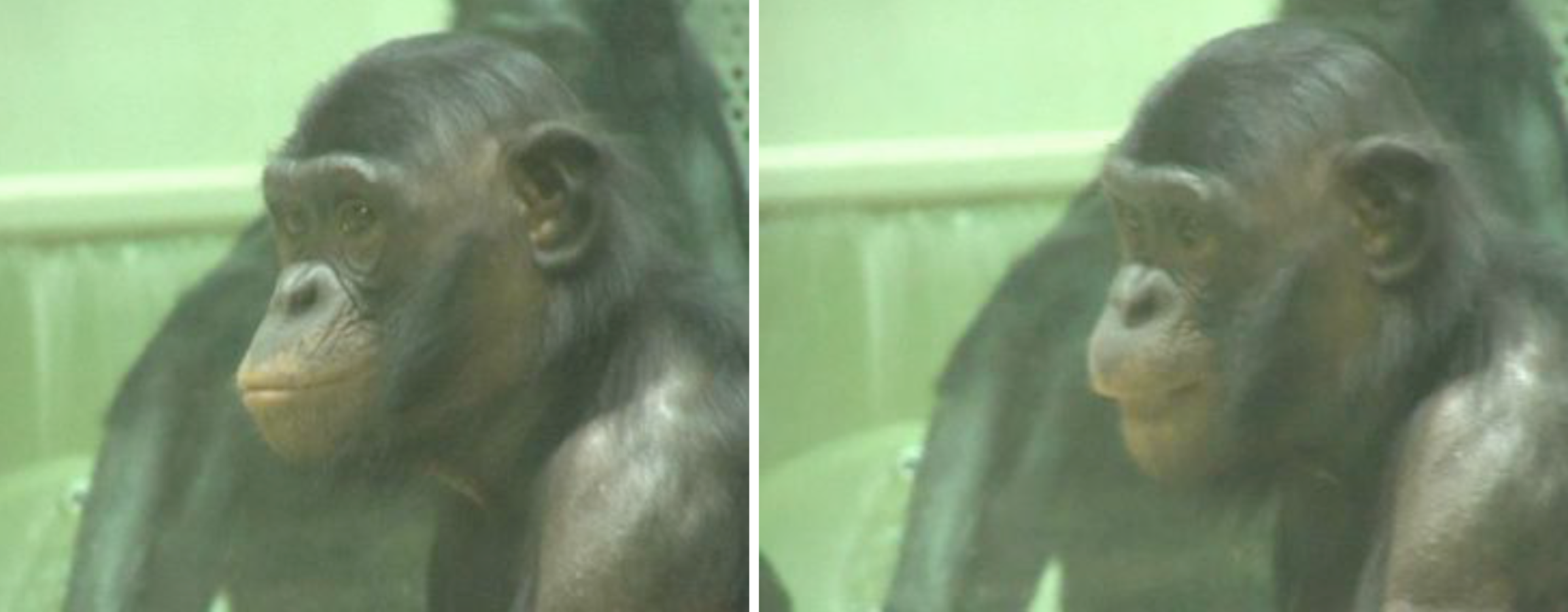

Supplement: Supplemental Information 37 — Other AUs present. Still frames from video by PK. [file peerj-13-19484-s037.png]
