# Supplementary material for: Adapting the facial action coding system for chimpanzees (Pan troglodytes) to bonobos (Pan paniscus): the ChimpFACS extension for bonobos
Source: PeerJ. 2025 Jun 13;13:e19484. doi: 10.7717/peerj.19484 (PMC12169169; doi:10.7717/peerj.19484)
Supplement: Supplemental Information 38 — ✓ - observed, x - not observed. Cells highlighted in grey are actions observed in bonobos. [file peerj-13-19484-s038.docx]

Table S1: Comparison between FACS Action Units (AU) for humans (Ekman, Friesen & Hager, 2002), chimpanzees (Vick et al., 2007), and bonobos according to the underlying musculature. ✓- observed, x - not observed. Cells highlighted in grey are actions observed in bonobos.

| **AU code** | **AU name** | **Underlying muscle** | **Human** | **Chimpanzee** | **Bonobo** |
| --- | --- | --- | --- | --- | --- |
| **AU1** | **Inner Brow Raiser** | Frontalis (medial) | ✓ | **x** | **x** |
| **AU2** | **Outer Brow Raiser** | Frontalis (lateral) | ✓ | **x** | **x** |
| **AU1+2** | **Brow Raiser** | Frontalis^1^ | x | ✓ | ✓ |
| **AU4** | **Brow Lowerer** | Procerus^1^, Depressor supercilii^1^, Corrugator supercilii^1^ | ✓ | x | x |
| **AU41** | **Glabella Lowerer** | Procerus^1^ | x | x | ✓ |
| **AU5** | **Upper Lid Raiser** | Levator palpebrae superioris and/or Orbicularis oculi^1^ | ✓ | ✓ | ✓ |
| **AU6** | **Cheek Raiser** | Orbicularis oculi^1^, pars orbitalis | ✓ | ✓ | ✓ |
| **AU7** | **Lid Tightener** | Orbicularis oculi^1^, pars palpebralis | ✓ | x | ✓ |
| **AU43** | **Eye closure** |  | ✓ | ✓ | ✓ |
| **AU45** | **Blink** |  | ✓ | ✓ | ✓ |
| **AU8** | **Lips Towards Each Other** | Orbicularis oris^1^ | ✓ | **x** | **x** |
| **AU9** | **Nose Wrinkler** | Levator labii superioris alaeque nasi^1^ | ✓ | ✓ | ✓ |
| **AU10** | **Upper Lip Raiser** | Levator labii superioris^1^ | ✓ | ✓ | ✓ |
| **AU11** | **Nasiolabial Furrow Deepener** | Zygomaticus minor^1^ | ✓ | **x** | **x** |
| **AU12** | **Lip Corner Puller** | Zygomaticus major^1^ | ✓ | ✓ | ✓ |
| **AU13** | **Cheek Puffer** | Caninus (or Levator anguli oris) ^1^ | ✓ | **x** | **x** |
| **AU14** | **Dimpler** | Buccinator^1^ | ✓ | **x** | **x** |
| **AU15** | **Lip Corner Depressor** | Depressor anguli oris^1^ | ✓ | **x** | **x** |
| **AU16** | **Lower Lip Depressor** | Depressor labii inferioris^1^ | ✓ | ✓ | ✓ |
| **AU160** | **Lower Lip Relax** | Relaxation of orbicularis oris/lower lip | **x** | ✓ | ✓ |
| **AU17** | **Chin Raiser** | Mentalis^1^ | ✓ | ✓ | ✓ |
| **AU18** | **Lip Pucker** | Incisivii labii (superioris and inferioris), Orbicularis oris^1^ | ✓ | **x** | ✓ |
| **AU20** | **Lip Stretcher** | Risorius | ✓ | **x** | **x** |
| **AU21** | **Neck Tightener** | Platysma myoides^1^ | ✓ | **x** | **x** |
| **AU22** | **Lip Funneler** | Orbicularis oris^1^ | ✓ | ✓ | ✓ |
| **AU23** | **Lip Tightener** |  | ✓ | **x** | **x** |
| **AU24** | **Lip Presser** |  | ✓ | ✓ | ✓ |
| **AU25** | **Lips Parted** |  | ✓ | ✓ | ✓ |
| **AU26** | **Jaw Drop** | Orbicularis oris^1^, Levator labii superioris^1^, Depressor labii inferioris^1^, non-mimetic muscles  Orbicularis oris^1^ | ✓ | ✓ | ✓ |
| **AU27** | **Mouth Stretch** |  | ✓ | ✓ | ✓ |
| **AU28** | **Lips Suck** |  | ✓ | ✓ | ✓ |
| **AU38** | **Nostril Dilator** | Nasalis^1^ | ✓ | x | ✓ |
| **AU39** | **Nostril Compressor** | Nasalis^1^, Depressor septi nasi^1^ | ✓ | x | ✓ |

^1^Described by Diogo et al (2017).
