# Supplementary material for: Adapting the facial action coding system for chimpanzees (Pan troglodytes) to bonobos (Pan paniscus): the ChimpFACS extension for bonobos
Source: PeerJ. 2025 Jun 13;13:e19484. doi: 10.7717/peerj.19484 (PMC12169169; doi:10.7717/peerj.19484)
Supplement: Supplemental Information 39 — ✓ - observed, x - not observed. Cells highlighted in grey were observed in bonobos. [file peerj-13-19484-s039.docx]

**Table S2:** **Comparison between FACS Action Descriptors (AD) for humans** (Ekman, Friesen & Hager, 2002)**, chimpanzees** (Vick et al., 2007) **and bonobos.** ✓- observed, x - not observed. Cells highlighted in grey were observed in bonobos.

| **AD code** | **AD name** | **Human** | **Chimpanzee** | **Bonobo** |
| --- | --- | --- | --- | --- |
| **AD19** | **Tongue Show** | ✓ | ✓ | ✓ |
| **AD29** | **Jaw Thrust** | ✓ | ✓ | **x** |
| **AD30** | **Jaw Sideways** | ✓ | ✓ | ✓ |
| **AU31** | **Jaw Clencher** | ✓ | **x** | **x** |
| **AD32** | **Bite** | ✓ | ✓ | ✓ |
| **AD33** | **Blow** | ✓ | ✓ | **x** |
| **AD34** | **Puff** | ✓ | **x** | **x** |
| **AD35** | **Cheek Suck** | ✓ | ✓ | **x** |
| **AD36** | **Bulge** | ✓ | **x** | **x** |
| **AD37** | **Lip Wipe** | ✓ | ✓ | **x** |
| **EAD1** | **Ears Forward** | **x** | **x** | ✓ |
| **EAD2** | **Ears Elevator** | **x** | **x** | ✓ |
| **EAD3** | **Ears Flattener** | **x** | **x** | ✓ |
