# Supplementary material for: Adapting the facial action coding system for chimpanzees (Pan troglodytes) to bonobos (Pan paniscus): the ChimpFACS extension for bonobos
Source: PeerJ. 2025 Jun 13;13:e19484. doi: 10.7717/peerj.19484 (PMC12169169; doi:10.7717/peerj.19484)
Supplement: Supplemental Information 40 — NA denotes instances where both coders agreed that a particular Action was not present in any of the clips. [file peerj-13-19484-s040.docx]

Table S3: Mean Wexler's index (Wexler, 1972) (1) and independent coding agreement for each AU, AD and EAD in the two coding rounds. NA denotes instances where both coders agreed that a particular Action was not present in any of the clips.

|  | **Round 1** | | **Round 2** | | |
| --- | --- | --- | --- | --- | --- |
|  | **CCC-PK** | **CCC-LO** | **CCC-PK** | **CCC-LO** | **Average of the 2 coder pairs** |
| Wexler's index | 0.77 | 0.74 | 0.81 | 0.81 | 0.81 |
| AU1+2: Brow Raiser | 0.82 | 0.57 | 0.82 | 0.75 | 0.79 |
| AU41: Glabella Lowerer | 0.00 | 0.67 | 0.33 | 0.75 | 0.54 |
| AU5: Upper Lid raiser | NA | NA | NA | NA | NA |
| AU6: Cheek Raiser | 0.00^1^ | 0.80 | 0.50^1^ | 0.80 | 0.65 |
| AU7: Lid Tightener | NA | 0.00^1^ | NA | 0.00^1^ | - |
| AU43: Eye Closure | NA | 0.00^1^ | NA | NA | NA |
| AU45: Blink | 0.95 | 0.86 | 0.95 | 0.86 | 0.91 |
| AU9: Nose Wrinkler | 0.63 | 0.71 | 0.71 | 0.71 | 0.71 |
| AU10: Upper Lip Raiser | 0.96 | 0.92 | 0.96 | 0.88 | 0.92 |
| AU12: Lip Corner Puller | 0.89 | 0.78 | 0.89 | 0.95 | 0.92 |
| AU16: Lower Lip Depressor | 0.75 | 0.80 | 0.75 | 0.80 | 0.78 |
| AU160: Lower Lip Relax | 1.00 | 0.50^1^ | 1.00 | 0.50^1^ | 0.75 |
| AU17: Chin Raiser | 1.00 | 0.67^1^ | 1.00 | 0.67^1^ | 0.84 |
| AU18: Lip Pucker | 0.40 | 0.62 | 0.60 | 0.62 | 0.61 |
| AU22: Lip Funneler | 0.89 | 0.94 | 0.89 | 0.94 | 0.92 |
| AU24: Lip Presser | 0.67 | 0.67 | 0.91 | 0.92 | 0.92 |
| AU25: Lips Part | 0.98 | 0.90 | 0.98 | 0.98 | 0.98 |
| AU26: Jaw Drop | 0.90 | 0.69 | 0.86 | 0.90 | 0.88 |
| AU27: Mouth Stretch | 0.86 | 0.80 | 0.86 | 1.00 | 0.93 |
| AU28: Lips Suck | 0.29 | 0.44 | 0.33 | 0.71 | 0.52 |
| AU38: Nostril Dilator | 0.00^1^ | 0.00^1^ | 0.00^1^ | 0.00^1^ | 0.00 |
| AU39: Nostril Compressor | NA | 0.00^1^ | NA | 0.00^1^ | - |
| AD19: Tongue Show | 1.00 | 1.00 | 1.00 | 1.00 | 1.00 |
| AD29: Jaw Thrust | NA | NA | NA | NA | NA |
| AD30: Jaw Sideways | 1.00 | 0.50 | 1.00 | 1.00 | 1.00 |
| AD35: Cheek Suck | 0.00^1^ | 0.00^1^ | 1.00 | 0.00^1^ | 0.50 |
| EAD1: Ears Forward | NA | 0.00^1^ | NA | 0.00^1^ | - |
| EAD2: Ears Elevator | 0.00^1^ | 0.67^1^ | 0.67^1^ | 0.67^1^ | 0.67 |
| EAD3: Ears Flattener | 0.67 | 0.86 | 0.86 | 0.86 | 0.86 |

^1^Low agreement due to rarely coded AUs/ADs/EADs (<3 occurrences), not due to low agreement between coders.
