# Supplementary material for: Adapting the facial action coding system for chimpanzees (Pan troglodytes) to bonobos (Pan paniscus): the ChimpFACS extension for bonobos
Source: PeerJ. 2025 Jun 13;13:e19484. doi: 10.7717/peerj.19484 (PMC12169169; doi:10.7717/peerj.19484)
Supplement: Supplemental Information 41 [file peerj-13-19484-s041.docx]

**Supplemental Information Text for:**

**Adapting the Facial Action Coding System for chimpanzees (*Pan troglodytes*) to bonobos (*Pan paniscus*): The ChimpFACS Extension for bonobos**

Catia Correia-Caeiro^1,2*^, Paul Kuchenbuch^3^, Linda S. Oña^1^, Franziska Wegdell^4,5^, Maël Leroux^6^, Andre Schuele^7^, Jared P. Taglialatela^8,9^, Simon W. Townsend^4,5^, Martin Surbeck^10,11^, Bridget M. Waller^12^, Katja Liebal^1,2^

^1^Human Biology & Primate Cognition, Institute of Biology, Leipzig University, Leipzig, Germany

^2^Department of Comparative Cultural Psychology, Max Planck Institute for Evolutionary Anthropology, Leipzig, Germany

^3^Independent researcher, Berlin, Germany

^4^Department of Evolutionary Anthropology, University of Zurich, Switzerland

^5^[Institute for the Interdisciplinary Study of Language Evolution](https://www.isle.uzh.ch/en.html), University of Zurich, Switzerland

^6^Éthologie Animale et Humaine (EthoS), CNRS, University of Rennes, Normandie University, France

^7^Berlin Zoo, Germany

^8^Ape Cognition and Conservation Initiative, USA

^9^Department of Ecology, Evolution, and Organismal Biology, Kennesaw State University, USA

^10^Department of Human Evolutionary Biology, Harvard University, Cambridge, USA

^11^Department of Human Behavior, Ecology and Culture, Max Planck Institute for Evolutionary Anthropology, Leipzig, Germany

^12^Department of Psychology, Nottingham Trent University, Nottingham, UK

***Corresponding author**

E-mail: catia_caeiro@hotmail.com (CCC)

**ORCID**: https://orcid.org/0000-0002-2819-6039

**SI1: Keywords hits in GoogleScholar**

**Table SI1:** Approximate number of results for keywords featuring the great ape species/genus, and for the combination of each great ape species/genus and “facial expressions” (both keywords using quotations, retrieved on the 19^th^ January 2025).

| **Keywords** | **Number of results** |
| --- | --- |
| Chimpanzee | 263.000 |
| **Bonobo** | **31.300** |
| Western gorilla | 2.100 |
| Eastern gorilla | 964 |
| Gorilla | 209.000 |
| Sumatran orangutan | 4.070 |
| Bornean orangutan | 2.840 |
| Tapanuli orangutan | 443 |
| Orangutan | 68.600 |
| Chimpanzee facial expressions | 13.400 |
| **Bonobo facial expressions** | **2.160** |
| Western gorilla facial expressions | 81 |
| Eastern gorilla facial expressions | 44 |
| Gorilla facial expressions | 7.740 |
| Sumatran orangutan facial expressions | 88 |
| Bornean orangutan facial expressions | 82 |
| Tapanuli orangutan facial expressions | 11 |
| Orangutan facial expressions | 2.740 |

**SI2: Facial morphology in bonobos**

The facial landmarks used in describing facial appearance changes of AUs are mostly the same in bonobos as in chimpanzees (**Fig. S2**). The most noticeable differences between these two species are the larger nasal shield in bonobos (**Fig. S3**, which resembles the shape of the nasal shield in gorillas, Correia-Caeiro et al., 2025), and upper eyelids and lips edge colouration (de Waal, 1988; Caldecott, 2005). These differences may increase visibility and/or detection rate of some movements in bonobos, since a larger nasal shield may increase visibility of nose AUs, and higher contrasting coloration may also increase visibility of mouth and eye AUs.‬‬‬‬‬‬‬‬‬‬‬‬‬‬‬‬‬‬ Chimpanzees and bonobos show further statistically significant differences in some facial features (Horn, 1979). In comparison to chimpanzees, bonobos have more paedomorphic characteristics (Horn, 1979, Lieberman et al., 2007). For example, bonobos have higher and more rounded frontal regions, and smaller and narrower browridges, with 72.3% of supra-orbital torus length in relation to chimpanzees (Horn, 1979). Despite individual variation, the frontal hair in adult chimpanzees is usually shorter, and some individuals may be more prone to baldness, while the frontal hair in adult bonobos is often longer and parted in the middle with conspicuous tufts (“sideburns”) sticking out at the sides (Kuchenbuch, 2010). Significant species differences in mandibular shape are also present between chimpanzees (i.e., more squared and angular) and bonobos (i.e., more rounded and gently sloping jawline) (Boughner & Dean, 2008). Chimpanzees infantile facial colouration is usually of a light cream colour, whilst infant bonobos already show the adult dark/black colouration. On the other hand, adult chimpanzees tend to have overall dark faces (black, dark brown, greyish spots), whilst bonobos (both adults and infants) usually present some cream coloured patches on their eyelids and around the lip areas, creating some contrasting features (personal observations, **Fig. S2** and **Fig. S4**). Traditionally, it was assumed that only humans had a conspicuous white sclera and that if some apes presented a sclera, this was a rare individual variation. Furthermore, in chimpanzees and bonobos it was deemed absent (Mearing & Koops, 2021). However, recent evidence has shown this assumption to be inaccurate (Clark et al., 2023; Kano, 2023). Whilst there seems to be large individual variation, some bonobos and chimpanzees present conspicuous white sclera, which contrasts with the dark iris and may aid in identification of eye movements (although see (Mearing et al., 2022) for the ongoing functionality debate). In addition, bonobo ears are smaller than those of chimpanzees and are often concealed by the longer hair of the head, which may make ear movements harder to detect.

Facial morphological sex differences are not very conspicuous both in chimpanzees and bonobos, particularly when compared with the other great apes (e.g., orangutans, *Pongo spp.*, gorillas, *Gorilla spp.*) that exhibit greater sexual dimorphism (Schaefer et al., 2004). Bonobos are indeed the least sexually dimorphic ape in terms of skull size and shape, even compared with humans (Schaefer et al., 2004). Nonetheless, male bonobos are slightly larger than females and present a larger facial width-to-height ratio (i.e., males have wider faces than females) (Martin et al., 2019).

**SI3: List of facilities housing bonobos**

**Table SI2:** List of facilities (e.g., zoos, sanctuaries, research institutions) housing bonobos from which videos were recorded and analysed for the development of the ChimpFACS Extension for bonobos. These videos were extracted from public sources (licensed as CC or with video owner permission) such as YouTube.com, Pixabay.com, etc., or belonged to private databases provided by the co-authors of this study.

| **Facility Name** | **Country** | **Video database source** |
| --- | --- | --- |
| Apenheul Zoo | Netherlands | Public |
| Berlin Zoo | Germany | Private (PK) |
| La Vallée des Singes | France | Public |
| Leipzig Zoo | Germany | Public |
| Lola Ya Bonobo sanctuary | DRC | Public |
| Ouwehands Zoo | Netherlands | Public |
| Planckendael Zoo | Belgium | Public |
| San Diego Zoo | USA | Public |
| Twycross Zoo | UK | Public |
| Wilhelma Zoo | Germany | Public |
| Wuppertal Zoo | Germany | Public |
| Ape Initiative Research Centre | USA | Private (ML) |

**SI4: Video databases of wild sites with bonobos**

Other than the captive populations mentioned above, two sources for video databases were used featuring wild bonobo populations to develop the ChimpFACS Extension for bonobos. One video of wild bonobos from the Bolobo territory was sourced from public databases (Mbou-Mon-Tour NGO, DRC). The second source for bonobo videos was from a large video database filmed by FW at the Kokolopori Bonobo Reserve (DRC). This database consisted of videos recorded with a Panasonic HC-VX11 4K camcorder during ad libitum and focal sampling. The videos were collected from May to October 2022 on habituated, wild bonobos belonging to four bonobo communities. FW adhered to the best practice guidelines for health monitoring and disease control in great ape populations (Gilardi et al., 2015): underwent quarantine, wore masks during data collection and maintained a 7m distance to the bonobos.

**SI5: Video contexts**

**Table SI3:** List of contexts annotated in the videos used in this work with a rough estimation of how many videos contained that context. Please note that we did not systematically categorised videos regarding contexts as this was beyond the scope of this work. Hence, these numbers are probably an under-estimation, as contexts were not always annotated. Also, the number of videos in the table does not reflect the total number of videos, as each video may contain several contexts.

| **Contexts** | **Number of videos** |
| --- | --- |
| Terrestrial behaviour | 234 |
| Arboreal behaviour | 95 |
| Aquatic behaviour | 14 |
| Resting | 84 |
| Grooming (allo- and self-) | 66 |
| Feeding | 59 |
| Play | 49 |
| Mother-infant interactions | 45 |
| Locomotion | 28 |
| Cognitive experiments | 21 |
| Sexual behaviour | 13 |
| Agonistic behaviour | 11 |
| Object manipulation | 8 |
| Nursing | 8 |
| Foraging | 7 |
| Human interactions (positive and negative) | 6 |
| Stressful situations | 4 |
| Other species interactions | 3 |
| Vocalisations | 3 |
| Termite fishing | 1 |
| Stereotypies | 1 |

**SI6: Action Descriptors and Ear Action Descriptors**

**Action Descriptors (ADs)** identify and describe more broad muscular movements or movements from non-mimetic muscles. The muscles responsible for ADs are not innervated by the facial nerve (cranial nerve VII (Reighard, 1900)), and so usually are not considered “facial expression” movements. However, these movements are often observed in combination with AUs, altering their visual appearance. Hence, section “A. Proposed muscular basis” described in the main text, is omitted below for all ADs, as either no movements are related to facial musculature or the musculature has not been clearly identified. In addition, section “D. Subtle differences between AUs” is included below wherever necessary, to help distinguishing between similar ADs or AUs.

In ChimpFACS only two ADs were described along photographic examples: AD30 - Jaw Sideways and AD35 - Cheek Suck. A few other ADs are identified as present in chimpanzees, but without description or examples (AD19 - Tongue Show, AD29 - Jaw Thrust, AD32 - Bite, AD33 - Blow, and AD37 - Lip Wipe), hence it is assumed the appearance changes in chimpanzees are the same as described in humans.

In Kuchenbuch's work (2010), other than AD30, AD19 is also described with picture examples. In the current sample, we also observed AD19, AD30, and AD32, but no other ADs. Hence, here we provide examples of these ADs, and similarly to AUs, the ADs for bonobos are only described with A-D sections if these are not included in the ChimpFACS or if there are significant differences in the appearance changes between species. It is possible other ADs are present in bonobos, so if other movements not here described are observed, these should be coded accordingly to the appearance changes from the human FACS.

**AD19 - Tongue Show**

**B. Appearance changes:**

1. The tongue is extended outwards and reaches at least the inner lower lip (e.g., **Fig. SI1**, **S61 Videos**).
2. The jaw must be lowered and the lips are separated. Therefore, this AD is always coded together with AU25+AU26 or AU25+AU27.

**C.** **Minimum criteria**: The tip of the tongue reaches at least the lower lip.


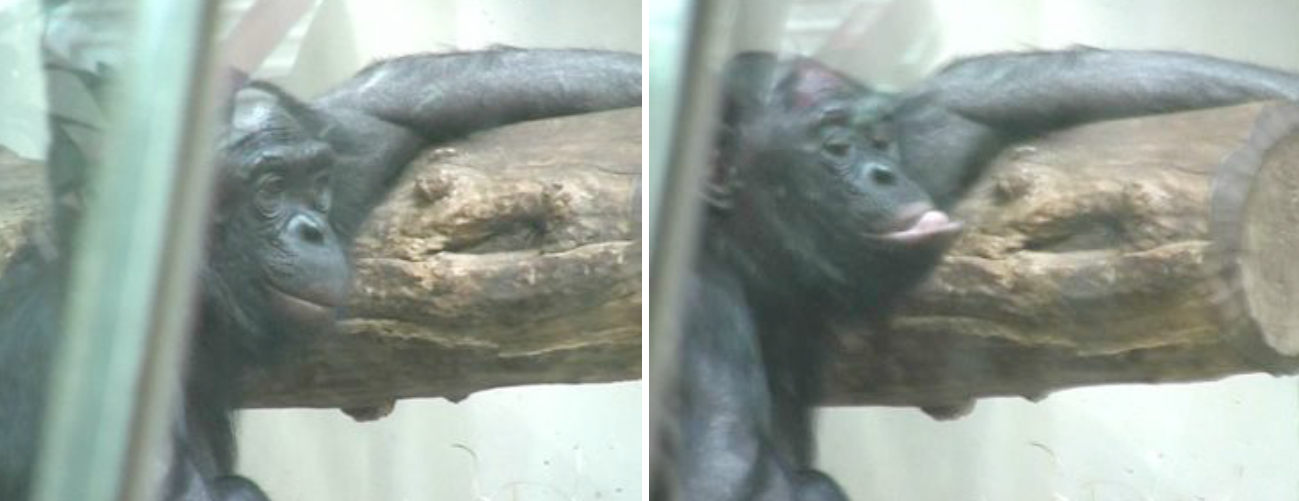


**Figure SI1:** AD19 - Tongue Show. Other AUs present. Still frames from video by PK.

**AD30 - Jaw Sideways**

**B. Appearance changes:**

1. The jaw is moved laterally to one side in relation to the midline (e.g., **S62-S63 Videos**).
2. If the mouth is open, the lower teeth are seen moving to one side.

**C. Minimum criteria to code AD30:** The jaw is moved to one side of the face.

**AD32 - Bite**

**B. Appearance changes:**

1. One of the lips is introduced in the mouth and held between the teeth, biting the lip (e.g., **S64 Videos**).

**C. Minimum criteria to code AD32:** A lip is held between the teeth.

**D. Subtle differences between AUs/ADs:** AD32 may be confused with AU28, but in the latter the lip is not held by the teeth, and is instead rolled over the teeth or sucked inside. In certain movements it may be hard to distinguish these two Actions, so when in doubt if the lip is being bitten, code AU28.

In addition to ADs, bonobos displayed ear movements, but no ear movements were included in the human nor the ChimpFACS. The ear musculature is present in humans only vestigially (with individual variation), but has been described for chimpanzees and bonobos in detail and comparisons revealed mostly similarities between these two species (Diogo et al., 2017). Three muscles seem to act to pull the ears in three directions: Auricularis superior, which pulls the ears up, Auricularis inferior, which pulls the ears downwards, and Auricularis posterior, which flattens the ears against the head. However, ear movements in bonobos were hard to pinpoint exactly when they were returning to neutral or being activated, and because of this, they are classified as ADs instead of AUs, i.e., Ear Action Descriptors (EADs).

Ear movements were also not included in Kuchenbuch's work (2010), but EADs have been included in FACS of other species (e.g., MaqFACS (Parr et al., 2010)). In the current work, we observed several examples of ear movements that resemble the appearance changes described in the MaqFACS, so we include EADs here for bonobos.

Ear visibility presents extensive variation in bonobos due to the longer hair parted to the sides, and are likely less visible in bonobos than in chimpanzees. Nonetheless, in the individuals in which the ears are visible, EADs can be coded. Caution is needed when coding EADs, as in some bonobos, during mastication, the ears might move frequently, giving the false appearance changes for EADs. However, these should not be coded as EADs, as they don't seem to be movements derived from the ear musculature, but perhaps more global movement from jaw and mastication muscles, as in e.g., **S65 Videos**.

**EAD1 - Ears Forward**

**B.** **Appearance changes:**

1. Pulls ears forward (e.g., **S66 Videos**).
2. Ears may become more visible and appear larger in frontal view or back view.
3. Conversely, ears may become less visible and appear smaller in side view.

**C.** **Minimum criteria**: Ears are clearly seen moving forward, away from the head.

**EAD2 - Ears Elevator**

**B.** **Appearance changes:**

1. Raises the ear upwards towards the top of the head (e.g., **S67 Videos**).

**C.** **Minimum criteria**: Ears are clearly seen moving upwards.

**EAD3 - Ears Flattener**

**B.** **Appearance changes:**

1. Pulls ears backwards, flattening them against the head (e.g., **S68 Videos**).
2. Ears may become less visible and appear smaller in frontal view. In back view, they may disappear against the head/hair.
3. Conversely, ears may become more visible and appear larger in side view.

**C.** **Minimum criteria**: Ears are clearly seen being pulled backwards or flattened against the head.

**D. Subtle differences between EADs:** EAD1 and EAD3 are mutually exclusive. However, it might be difficult to determine the position of the neutral, since these two movements happen in a continuum. EAD2 is easier to identify as there is no opposite movement, but it appears to be a subtler movement than the other two. The neutral position of the ears may present individual variation, so unless the coder is highly familiar with the individual to determine this position, these movements will always be coded as each individual movement independently of where is the actual neutral position (unless the ear is seen moving twice forward without moving back consecutively).

**Other Action Descriptors**

Other miscellaneous movements classified as ADs including head (e.g., **S69 Video**) and eye direction (e.g., **Fig. SI2**, **S70 Videos**), gross behaviours, and visibility status are briefly described next.

**Head and Eye Action Descriptors**

Head movements recorded in video are possible to code based on the movement itself, or due to changes in the relative position of the head and body, usually from a neutral position (or anatomical standard position, where head and eyes are facing forward, head at a 90º angle).

**AD51 - Head Turn Left:** The head moves to the left along a vertical axis.

**AD52 - Head Turn Right:** The head moves to the right along a vertical axis.

**AD53 - Head Up:** The head moves upwards.

**AD54 - Head Down:** The head moves downwards.

**AD55 - Head Tilt Left:** The head rotates tilting to the left side (e.g., **S69** **Video**).

**AD56 - Head Tilt Right:** The head rotates tilting to the right side.

**AD57 - Head Forward:** The head moves forward and away from the body. In a neutral position, the neck is not well-defined, but with AD57, the neck elongates forward and becomes visible.

**AD58 - Head Back:** The head moves back and towards the body.

In humans, eye movements are easy to detect as the coloured iris is surrounded by a large white sclera. In bonobos, like other non-human primates, eye movements can be more difficult to detect, since the eyes usually have a dark brown iris without any sclera visible in the neutral position. Bonobo eyes have a black round pupil that reacts to light, although in most lighting conditions the pupil is hard to distinguish from the iris. A more or less portion of white sclera can become visible on the outer eye corner when the eyeball moves laterally (e.g., **Fig. SI2, S70 Videos**), aiding the detection of some eye movements. How much sclera is visible during eye movements seems to present variation between individuals.

Head and eye positions tend to influence how each are perceived by humans (Otsuka & Clifford, 2018), so it is important to look for cues other than position alone, such as sclera visibility or clear movement.

**AD61 - Eyes Turn Left:** The eyes move to the left. In large movements the sclera may show on the right side of the eye (e.g., **Fig. SI2**).

**AD62 - Eyes Turn Right:** The eyes move to the right. In large movements the sclera may show on the left side of the eye (e.g., **S70 Videos**).

**AD63 - Eyes Up:** The eyes move upwards.

**AD64 - Eyes Down:** The eyes move downwards.


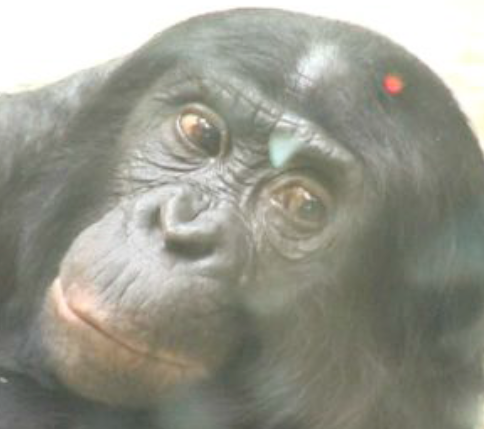

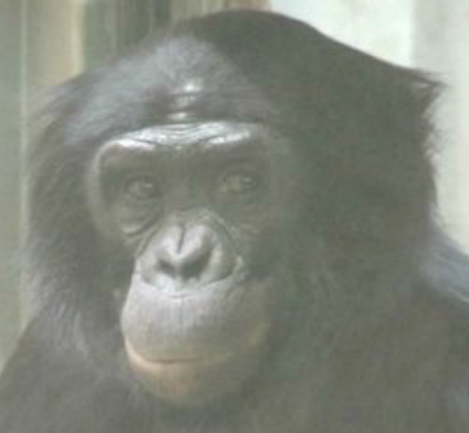

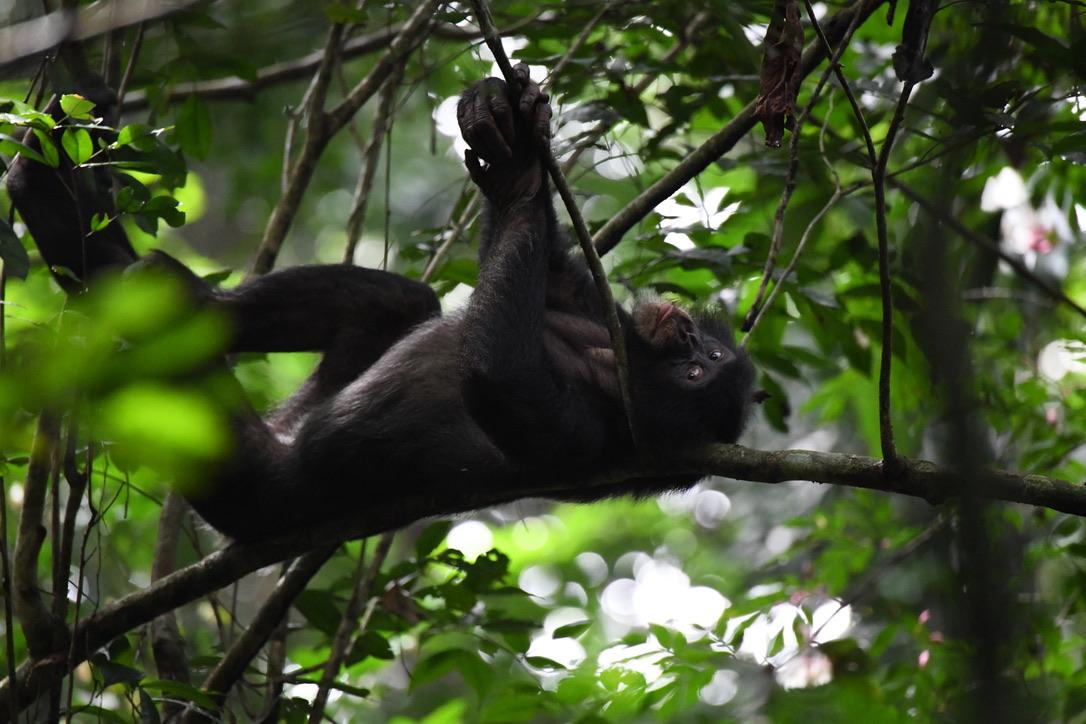


**Figure SI2:** **AD61 – Eyes Turn Left**, with sclera exposure on the right corner of the eyes. Left and centre: still frames from video by PK, right: picture by FW/Kokolopori Bonobo Research Project.

**Gross behaviour codes**

Gross behaviour codes are more general categories of behaviour that are not based on facial actions and may or may not involve the face. These codes are useful to score alongside AUs and other ADs as they can impact on or modify appearance changes.

**AD40 - Sniff:** This AD refers to the act of smelling, where global movement with varied intensity is observed around the nose and nostrils. It can be accompanied by nostril AUs, although these movements can also occur independently.

**AD50 - Vocalisations:** Bonobos display a range of vocalisations and most of them change the movement of the mouth and nose. This should be accounted for when coding facial behaviour.

**AD80 - Swallow:** Passing food, water or saliva from the mouth to the oesophagus through the action of a range of non-mimetic muscles. In bonobos, swallowing can produce movement on the hair on the anterior side of the neck and just below the neck.

**AD81 - Chewing:** Feeding-related behaviour when food is introduced into the mouth and the jaws are moved repeatedly to break down the food, involves several AUs and ADs described above and in the main text (e.g., AU25+AU26+AU30). However, AD81 should be coded instead of these mastication AUs and ADs whenever mouth movements are primarily due to feeding-related behaviours, as these movements are not considered related to communication or emotion.

**Visibility codes**

**AD 70 - Frontal Region Not Visible:** When the browridge and frontal region cannot be clearly seen and coded.

**AD 71 - Eyes Not Visible:** When the eyes cannot be clearly seen and coded.

**AD 72 - Lower Face Not Visible:** When the lower face cannot be clearly seen and coded.

**AD 73 - Entire Face Not Visible:** When the entire face is out of view and cannot be clearly seen and coded.

**SI7: Application of ChimpFACS to bonobos: inter-rater coding reliability**

We tested inter-rater reliability between three coders (CCC, PK, and LO) by coding 28 clips of bonobo videos (not used for AUs examples described in the results section). CCC is certified in HumanFACS (Ekman, Friesen & Hager, 2002a) and in all the AnimalFACS developed to date (Parr et al., 2010; Waller et al., 2012, 2013; Correia-Caeiro et al., 2013, 2022; Wathan et al., 2015; Correia-Caeiro, Burrows & Waller, 2017; Correia-Caeiro, Holmes & Miyabe-Nishiwaki, 2021), including ChimpFACS (Vick et al., 2007); PK is certified in ChimpFACS (Vick et al., 2007); LO is certified in HumanFACS (Ekman, Friesen & Hager, 2002a), GibbonFACS (Waller et al., 2012), and DogFACS (Waller et al., 2013).

The inter-rater reliability aimed to: 1) verify that the AUs from ChimpFACS could be coded in bonobos; 2) assess whether any movements in bonobos, can be reliably coded; and 3) ensure that all coders can reliably identify ChimpFACS movements in bonobos, accounting for morphological differences between chimpanzees and bonobos. The low agreement for particular AUs was then addressed by revising appearance changes descriptions to enhance AU identification and conducting a subsequent round of coding. If agreement remained low after a second round, the challenging AUs were discussed regarding the underlying issues affecting their coding. From one coding round to another, two of the coders (PK and LO) were blind to each other’s and the third coder’s (CCC) scores.

The overall reliability between coders (Wexler’s index (Wexler, 1972), see Eq. (1)) and the AUs independent coding agreement (calculated through the average of each AU agreement) in a first round of coding (**Table S3**) indicated a good overall agreement between coders of 77% (CCC-PK) and 74% (CCC-LO) (Ekman, Friesen & Hager, 2002b; Parr et al., 2007). However, some AUs still had low independent agreement, which were discussed between the coders to flag what led to such differences. A second coding round by both pairs of coders helped clarify the description of appearance changes for some movements and improved reliability. In this round of coding, we obtained a mean of 81% overall agreement on Wexler’s index (1) (Wexler, 1972) for both coder pairs (CCC-PK and CCC-LO), and also a good agreement on most AUs (**Table S3**). Further discussion of the few AUs with lower final average agreement (below 70%, i.e., AU41, AU6, AU18, AU28, EAD2) can be found in each respective AU section. These AUs are likely the most challenging to detect and be reliably coded, by what caution is recommended when including these AUs in the coding of bonobos. Additional video examples were also added to SI to aid in the coding of these AUs.

$${\left( 1 \right)\mathrm{Wexler}}^{'}s index= \frac{\left( Number of AUs on which coder 1 and Coder 2 agreed \right)\times2}{The total number of AUs scored by the two coders}$$

(1)

**SI8: AnimalFACS and species**

**Table SI4:** Overview of the AnimalFACS developed till date, the respective extensions, and which and how many species each system has been developed for or tested for application.

| **FACS** | **Targeted species** | **Extensions** | **Number of species** |
| --- | --- | --- | --- |
| ChimpFACS | Chimpanzees  (*Pan troglodytes*)  (Vick et al., 2007) | Bonobos  (*P. paniscus*)  (current work) | 2 |
| OrangFACS^1^ | Bornean orangutans  (*Pongo pygmaeus*)  Sumatran orangutan  (*P. abelii*)  (Correia-Caeiro et al., 2013) |  | 2 |
| GorillaFACS | Eastern gorilla  (*Gorilla beringei*)  Western gorilla  (*G. gorilla*)  (Correia-Caeiro et al., 2025)‬‬‬‬‬‬ |  | 2 |
| GibbonFACS | Siamangs  (*Symphalangus syndactylus*)  Pileated gibbon  (*Hylobates pileatus*)  Silvery gibbon  *(H. moloch)*  Muelleri gibbon  *(H. muelleri)*  Yellow-cheeked gibbon  (*Nomascus gabriellae*)  Northern white-cheeked gibbon  (*N. leucogenys*)  Southern white-cheeked gibbon  (*N. siki*) (Waller et al., 2012) |  | 7 |
| MaqFACS | Rhesus macaques  (*Macaca mulatta*)  (Parr et al., 2010) | Barbary macaques  (*M. sylvanus*)  (Julle-Danière et al., 2015)  Japanese macaques  (*M. fuscata*)  (Correia-Caeiro, Holmes & Miyabe-Nishiwaki, 2021)  Crested macaques  (*M. nigra*)  (Clark et al., 2020) | 4 |
| CalliFACS | Common marmosets  (*Callithrix jacchus*)  (Correia-Caeiro et al., 2022) |  | 1 |
| DogFACS | Domestic dogs  (*Canis familiaris*)  (Waller et al., 2013) |  | 1 |
| CatFACS | Domestic cats  (*Felis catus*)  (Correia-Caeiro, Burrows & Waller, 2017) |  | 1 |
| EquiFACS | Domestic horses  (*Equus ferus caballus*)  (Wathan et al., 2015) |  | 1 |
| Total | | | 21 |

^1^Tapanuli orangutans are not included in OrangFACS as they were only identified as a new species in 2017 (Nater et al., 2017).

**References**

Boughner JC, Dean MC. 2008. Mandibular Shape, Ontogeny and Dental Development in Bonobos (Pan paniscus) and Chimpanzees (Pan troglodytes). *Evolutionary Biology* 35:296–308. DOI: 10.1007/s11692-008-9043-6.

Caldecott J. 2005. Chimpanzee and bonobo overview. In: Caldecott J, Miles L eds. *World Atlas of Great Apes and their Conservation*. Berkeley, USA: University of California Press, 43–51.

Clark IR, Lee KC, Poux T, Langergraber KE, Mitani JC, Watts D, Reed J, Sandel AA. 2023. White sclera is present in chimpanzees and other mammals. *Journal of human evolution* 176:103322. DOI: 10.1016/j.jhevol.2022.103322.

Clark PR, Waller BM, Burrows AM, Julle-Danière E, Agil M, Engelhardt A, Micheletta J. 2020. Morphological variants of silent bared-teeth displays have different social interaction outcomes in crested macaques (Macaca nigra). *American Journal of Physical Anthropology* 173:411–422. DOI: 10.1002/ajpa.24129.

Correia-Caeiro C, Burrows AM, Waller BM. 2017. Development and application of CatFACS: Are human cat adopters influenced by cat facial expressions? *Applied Animal Behaviour Science* 189:66–78. DOI: 10.1016/j.applanim.2017.01.005.

Correia-Caeiro C, Burrows A, Wilson DA, Abdelrahman A, Miyabe-Nishiwaki T. 2022. CalliFACS: The common marmoset Facial Action Coding System. *PLOS ONE* 17:e0266442. DOI: 10.1371/journal.pone.0266442.

Correia-Caeiro C, Costa R, Hayashi M, Burrows A, Pater J, Miyabe-Nishiwaki T, Richardson JL, Robbins MM, Waller B, Liebal K. 2025. GorillaFACS: The Facial Action Coding System for the Gorilla spp. *PLOS ONE* 20:e0308790. DOI: 10.1371/journal.pone.0308790.

Correia-Caeiro C, Holmes K, Miyabe-Nishiwaki T. 2021. Extending the MaqFACS to measure facial movement in Japanese macaques (Macaca fuscata) reveals a wide repertoire potential. *PLOS ONE* 16:e0245117. DOI: 10.1371/journal.pone.0245117.

Correia-Caeiro C, Waller BM, Zimmermann E, Burrows AM, Davila-Ross M. 2013. OrangFACS: A muscle-based facial movement coding system for orangutans (Pongo spp.). *International Journal of Primatology* 34:115–129. DOI: 10.1007/s10764-012-9652-x.

Diogo R, Shearer B, Potau JM, Pastor JF, de Paz FJ, Arias-Martorell J, Turcotte C, Hammond A, Vereecke E, Vanhoof M, Nauwelaerts S, Wood B. 2017. Head and Neck Musculature. In: Diogo R, Shearer B, Potau JM, Pastor JF, de Paz FJ, Arias-Martorell J, Turcotte C, Hammond A, Vereecke E, Vanhoof M, Nauwelaerts S, Wood B eds. *Photographic and Descriptive Musculoskeletal Atlas of Bonobos: With Notes on the Weight, Attachments, Variations, and Innervation of the Muscles and Comparisons with Common Chimpanzees and Humans*. Cham: Springer International Publishing, 5–47. DOI: 10.1007/978-3-319-54106-8_2.

Ekman P, Friesen WV, Hager JC. 2002a. *Facial Action Coding System (FACS): manual*. Salt Lake City: Research Nexus.

Ekman P, Friesen WV, Hager JC. 2002b. *FACS investigator’s guide*. Salt Lake City: Research Nexus.

Gilardi KV, Gillespie TR, Leendertz FH, Macfie EJ, Travis DA, Whittier CA, Williamson EA. 2015. *Best Practice Guidelines for Health Monitoring and Disease Control in Great Ape Populations*. Gland, Switzerland: IUCN SSC Primate Specialist Group.

Horn AD. 1979. The taxonomic status of the bonobo chimpanzee. *American Journal of Physical Anthropology* 51:273–281. DOI: 10.1002/ajpa.1330510213.

Julle-Danière É, Micheletta J, Whitehouse J, Joly M, Gass C, Burrows AM, Waller BM. 2015. MaqFACS (Macaque Facial Action Coding System) can be used to document facial movements in Barbary macaques (Macaca sylvanus). *PeerJ* 3:e1248. DOI: 10.7717/peerj.1248.

Kano F. 2023. Evolution of the uniformly white sclera in humans: critical updates. *Trends in Cognitive Sciences* 27:10–12. DOI: 10.1016/j.tics.2022.09.011.

Kuchenbuch P. 2010. Facial Behaviour in a Group of Captive Bonobos (Pan paniscus) - Application of a New Methodological Approach (ChimpFACS). Freien Universität Berlin.

Lieberman DE, Carlo J, Ponce de León M, Zollikofer CPE. 2007. A geometric morphometric analysis of heterochrony in the cranium of chimpanzees and bonobos. *Journal of Human Evolution* 52:647–662. DOI: 10.1016/j.jhevol.2006.12.005.

Martin JS, Staes N, Weiss A, Stevens JMG, Jaeggi AV. 2019. Facial width-to-height ratio is associated with agonistic and affiliative dominance in bonobos (Pan paniscus). *Biology Letters* 15:20190232. DOI: 10.1098/rsbl.2019.0232.

Mearing AS, Burkart JM, Dunn J, Street SE, Koops K. 2022. The evolutionary drivers of primate scleral coloration. *Scientific Reports* 12:14119. DOI: 10.1038/s41598-022-18275-9.

Mearing AS, Koops K. 2021. Quantifying gaze conspicuousness: Are humans distinct from chimpanzees and bonobos? *Journal of Human Evolution* 157:103043. DOI: 10.1016/j.jhevol.2021.103043.

Nater A, Mattle-Greminger MP, Nurcahyo A, Nowak MG, Manuel M de, Desai T, Groves C, Pybus M, Sonay TB, Roos C, Lameira AR, Wich SA, Askew J, Davila-Ross M, Fredriksson G, Valles G de, Casals F, Prado-Martinez J, Goossens B, Verschoor EJ, Warren KS, Singleton I, Marques DA, Pamungkas J, Perwitasari-Farajallah D, Rianti P, Tuuga A, Gut IG, Gut M, Orozco-terWengel P, Schaik CP van, Bertranpetit J, Anisimova M, Scally A, Marques-Bonet T, Meijaard E, Krützen M. 2017. Morphometric, Behavioral, and Genomic Evidence for a New Orangutan Species. *Current Biology* 27:3487-3498.e10. DOI: 10.1016/j.cub.2017.09.047.

Otsuka Y, Clifford CWG. 2018. Influence of head orientation on perceived gaze direction and eye-region information. *Journal of Vision* 18:15–15. DOI: 10.1167/18.12.15.

Parr LA, Waller BM, Burrows AM, Gothard KM, Vick SJ. 2010. Brief communication: MaqFACS: A muscle-based facial movement coding system for the rhesus macaque. *American Journal of Physical Anthropology* 143:625–630. DOI: 10.1002/ajpa.21401.

Parr LA, Waller BM, Vick SJ, Bard KA. 2007. Classifying chimpanzee facial expressions using muscle action. *Emotion* 7:172–181. [DOI: 10.1037/1528-3542.7.1.172](https://psycnet.apa.org/doi/10.1037/1528-3542.7.1.172)

Reighard J. 1900. Scientific Books: Text-Book of Vertebrate Zoology. *Science* 11:344–346. DOI: 10.1126/science.11.270.344.

Schaefer K, Mitteroecker P, Gunz P, Bernhard M, Bookstein FL. 2004. Craniofacial sexual dimorphism patterns and allometry among extant hominids. *Annals of Anatomy - Anatomischer Anzeiger* 186:471–478. DOI: 10.1016/S0940-9602(04)80086-4.

Vick SJ, Waller BM, Parr LA, Pasqualini MCS, Bard KA. 2007. A cross-species comparison of facial morphology and movement in humans and chimpanzees using the Facial Action Coding System (FACS). *Journal of Nonverbal Behavior* 31:1–20. DOI: 10.1007/s10919-006-0017-z.

de Waal FBMD. 1988. The Communicative Repertoire of Captive Bonobos (Pan Paniscus), Compared To That of Chimpanzees. *Behaviour* 106:183–251. DOI: 10.1163/156853988X00269.

Waller BM, Lembeck M, Kuchenbuch P, Burrows AM, Liebal K. 2012. GibbonFACS: A muscle-based facial movement coding system for hylobatids. *International Journal of Primatology* 33:809–821. DOI: 10.1007/s10764-012-9611-6.

Waller BM, Peirce K, Correia-Caeiro C, Scheider L, Burrows AM, McCune S, Kaminski J. 2013. Paedomorphic facial expressions give dogs a selective advantage. *PLOS ONE* 8:e82686. DOI: 10.1371/journal.pone.0082686.

Wathan J, Burrows AM, Waller BM, McComb K. 2015. EquiFACS: The Equine Facial Action Coding System. *PLOS ONE* 10:e0131738. DOI: 10.1371/journal.pone.0131738.

Wexler DA. 1972. Method for unitizing protocols of descriptions of emotional states. Journal of Supplemental Abstracts Service, Catalogue of Selected Documents in Psychology, American Psychological Association 2:116.
